# Supplementary material for: Bayesian analyses indicate bivalves did not drive the downfall of brachiopods following the Permian-Triassic mass extinction
Source: Nat Commun. 2023 Sep 9;14:5566. doi: 10.1038/s41467-023-41358-8 (PMC10492784; doi:10.1038/s41467-023-41358-8)
Supplement: Supplementary file 1 — Supplementary Info [file 41467_2023_41358_MOESM1_ESM.pdf]

Supplementary Figures and Tables of 'Bayesian analyses indicate bivalves did not drive the downfall of brachiopods following the Permian-Triassic mass extinction'

Supplementary Figs. 1–2. Bayesian estimates of diversification rates and diversity of post-Cambrian brachiopods and bivalves. The plots of rate shift frequencies indicate the significance of the rate shift (based on Bayes Factor). Shaded areas in rate plots indicate 95% high posterior density intervals. Shaded areas in diversity plot contain values of different replications that incorporate age uncertainties of fossil occurrences.

Supplementary Figs. 3–4. Bayesian estimates of diversification rates and diversity of Permian–Jurassic brachiopods and bivalves. The plots of rate shift frequencies indicate the significance of the rate shift (based on Bayes Factor). Shaded areas in rate plots indicate 95% high posterior density intervals. Shaded areas in diversity plot contain values of different replications that incorporate age uncertainties of fossil occurrences.

Supplementary Figs. 5–9. Bayesian estimates of diversification rates and diversity of Permian–Jurassic brachiopods with different lifestyles (including epifaunal, infaunal, pedicle-attached, cemented, and reclining). The plots of rate shift frequencies indicate the significance of the rate shift (based on Bayes Factor). Shaded areas in rate plots indicate 95% high posterior density intervals. Shaded areas in diversity plot contain values of different replications that incorporate age uncertainties of fossil occurrences.

Supplementary Figs. 10–11. Bayesian estimates of diversification rates and diversity of brachiopods that extinct in the PTME (PTe) and survived or originated after the PTME (PTs). The plots of rate shift frequencies indicate the significance of the rate shift (based on Bayes Factor). Shaded areas in rate plots indicate 95% high posterior density intervals. Shaded areas in diversity plot contain values of different replications that incorporate age uncertainties of fossil occurrences.

Supplementary Figs. 12–18. Bayesian estimates of diversification rates and diversity of Permian–Jurassic bivalves with different lifestyles (including epifaunal, infaunal, epibyssate, cemented, reclining, shallow infaunal, and deep infaunal). The plots of rate shift frequencies indicate the significance of the rate shift (based on Bayes Factor). Shaded areas in rate plots indicate 95% high posterior density intervals. Shaded areas in diversity plot contain values of different replications that incorporate age uncertainties of fossil occurrences.

Supplementary Figs. 19–26. Bayesian estimates of diversification rates and diversity of brachiopods and bivalves in the four regions (NW Tethys north-western Tethys, SW Tethys south-western Tethys, E Tethys, eastern Tethys, and N Panthalassa north Panthalassa). The plots of rate shift frequencies indicate the significance of the rate shift (based on Bayes Factor). Shaded areas in rate plots indicate 95% high posterior density intervals. Shaded areas in diversity plot contain values of different replications that incorporate age uncertainties of fossil occurrences.

Supplementary Fig. 27. Proportion of diversity of bivalves belonging to different ecological groups. The proportion of infaunal group gradually increased in the Triassic–Jurassic.

Supplementary Fig. 28. Trace plots of PyRate analyses with or without the Gamma model. The analysed datasets were the Permian–Jurassic brachiopods and Permian–Jurassic reclining bivalves. The estimated preservation rate in the Wuchiapingian is selected to show their difference. Every plot includes ten lines representing ten age-randomized replications of a dataset. If the Gamma model (incorporating

the heterogenous preservation rate among lineages) is used, it is possible for the estimated rate to jump to another value even with a high number of iterations. Besides, values from different replicates showed large variability. When the Gamma model is not used, the analysis reaches convergent quickly and values from replications are close. (The number of iterations in the ten replications may vary, but these plots already show that an analysis with the Gamma model is difficult to converge.)

Supplementary Fig. 29. Comparison of parameters estimated by PyRate using different algorithms (M-H vs. Gibbs, Gamma vs. non-Gamma). All correlations are significant ($p < 0.01$). The dataset analysed is the reclining bivalve dataset. Ts and Te, time of origination and extinction; q, preservation rate.

Supplementary Fig. 30. Comparison of diversities estimated by mcmcDivE and ltt. The left panel shows raw result; the right panel shows diversities rescaled to 0-1. The curves estimated by mcmcDivE show more ups and downs that do not correspond to the estimated diversification dynamics.

Supplementary Fig. 31. Factors included in the MBD analyses (except the brachiopod and bivalve diversities). All factors are rescaled to 0–1.

Supplementary Figs. 32–35. Palaeobiogeography of brachiopods and bivalves indicated by network analysis and partition around medoids clustering. Each colour represents a group. Every interval was analysed individually. Cells with the same colour in two intervals does not mean they belong to one group.

Supplementary Fig. 36. Spatial windows and their movement over time. The blue, red, green, and orange windows represent the NW Tethys, SW Tethys, E Tethys, and N Panthalassa, respectively. Filled dots represent cells retained after minimum spanning tree (MST) standardisation. Open dots represent cells discarded in the process.

Supplementary Fig. 37. Minimum spanning tree (MST) lengths of occurrences in the four regions before and after spatial standardisation. The variance of MST lengths over time are declined after standardisation.

Supplementary Fig. 38. Minimum spanning tree (MST) lengths post-Cambrian brachiopods and bivalves. Mesozoic and Cenozoic bivalves had longer MST lengths than brachiopods, but their difference is much less prominent than difference in diversity (e.g., MST lengths of Triassic–Cretaceous brachiopods were not quite lower than those of Cenozoic bivalves, but the brachiopod diversity was very low compared to bivalve diversity), suggesting that the decline of brachiopod diversity and increase of bivalves were real and less likely to be biased by spatial coverage.

Supplementary Fig. 39. Diversities and diversification rates of post-Cambrian brachiopods generated by the divDyn package. Diversities are calculated using the range-through and corrected sampled-in-bin methods. Per-capita rate and second-for-third rate are shown. The left panel shows the results on the raw data. The right panel shows the results on SQS subsampled ($q = 0.5$) data. The shaded areas in the right panel represent quantiles of rates calculated for 1000 replications, and the solid lines represent mean values.

Supplementary Fig. 40. Diversities and diversification rates of post-Cambrian bivalves generated by the divDyn package. Diversities are calculated using the range-through and corrected sampled-in-bin methods. Per-capita rate and second-for-third rate are shown. The left panel shows the results on the raw data. The right panel shows the results on SQS subsampled ($q = 0.5$) data. The shaded areas in the right panel represent quantiles of rates calculated for 1000 replications, and the solid lines represent mean values.

Supplementary Fig. 41. Diversities and diversification rates of Permian-Jurassic brachiopods generated by the divDyn package. Diversities are calculated using the range-through and corrected sampled-in-bin methods. Per-capita rate and second-for-third rate are shown. The left panel shows the results on the raw data. The right panel shows the results on SQS subsampled ($q = 0.5$) data. The shaded areas in the right panel represent quantiles of rates calculated for 1000 replications, and the solid lines represent mean values.

Supplementary Fig. 42. Diversities and diversification rates of Permian-Jurassic bivalves generated by the divDyn package. Diversities are calculated using the range-through and corrected sampled-in-bin methods. Per-capita rate and second-for-third rate are shown. The left panel shows the results on the raw data. The right panel shows the results on SQS subsampled (q = 0.5) data. The shaded areas in the right panel represent quantiles of rates calculated for 1000 replications, and the solid lines represent mean values.

Supplementary Fig. 43. Correlations (Spearman's rho) between per-capita rates of post-Cambrian brachiopods and bivalves. The rates are based on raw data and are standardised. p values are Bonferroni-adjusted.

Supplementary Fig. 44. Correlations (Spearman's rho) between per-capita rates of Ordovician–Triassic brachiopods and bivalves. The rates are based on raw data and are standardised. p values are Bonferroni-adjusted.

Supplementary Fig. 45. Correlations (Spearman's rho) between per-capita rates of post-Triassic brachiopods and bivalves. The rates are based on raw data and are standardised. p values are Bonferroni-adjusted.

Supplementary Fig. 46. Correlations (Spearman's rho) between per-capita rates of post-Cambrian brachiopods and bivalves. The rates are based on SQS subsampled data and are standardised. p values are Bonferroni-adjusted.

Supplementary Fig. 47. Correlations (Spearman's rho) between per-capita rates of Ordovician–Triassic brachiopods and bivalves. The rates are based on SQS subsampled data and are standardised. p values are Bonferroni-adjusted.

Supplementary Fig. 48. Correlations (Spearman's rho) between per-capita rates of post-Triassic brachiopods and bivalves. The rates are based on SQS subsampled data and are standardised. p values are Bonferroni-adjusted.

Supplementary Fig. 49. Correlations between per-capita rates of brachiopods and bivalves and the resulted residuals. The rates based on the raw data.

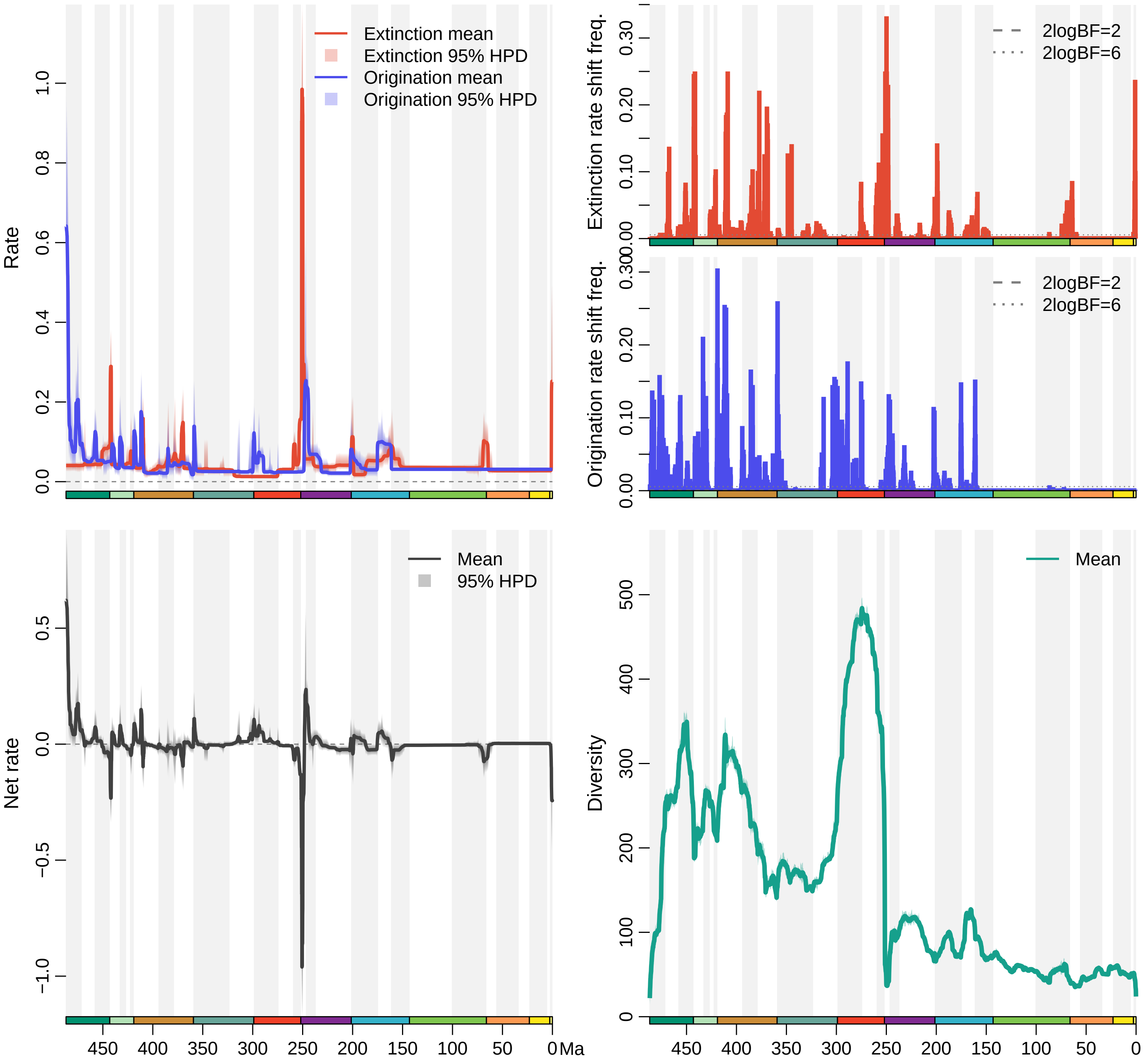

Supplementary Fig. 1. Diversitification rates, rate shift frequencies, and diversity of post−Cambrian brachiopods.

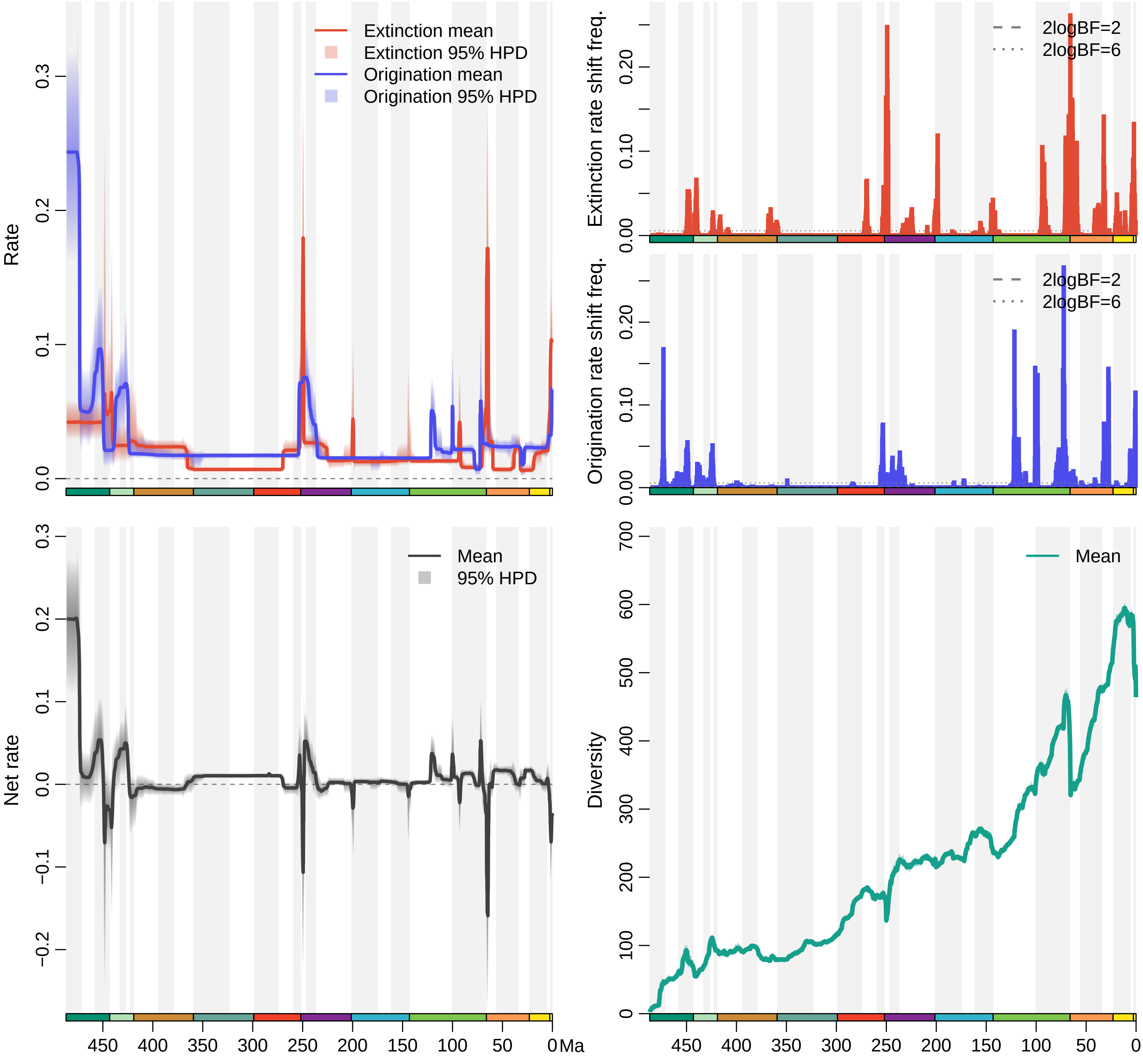

Supplementary Fig. 2. Diversitification rates, rate shift frequencies, and diversity of post−Cambrian bivalves.

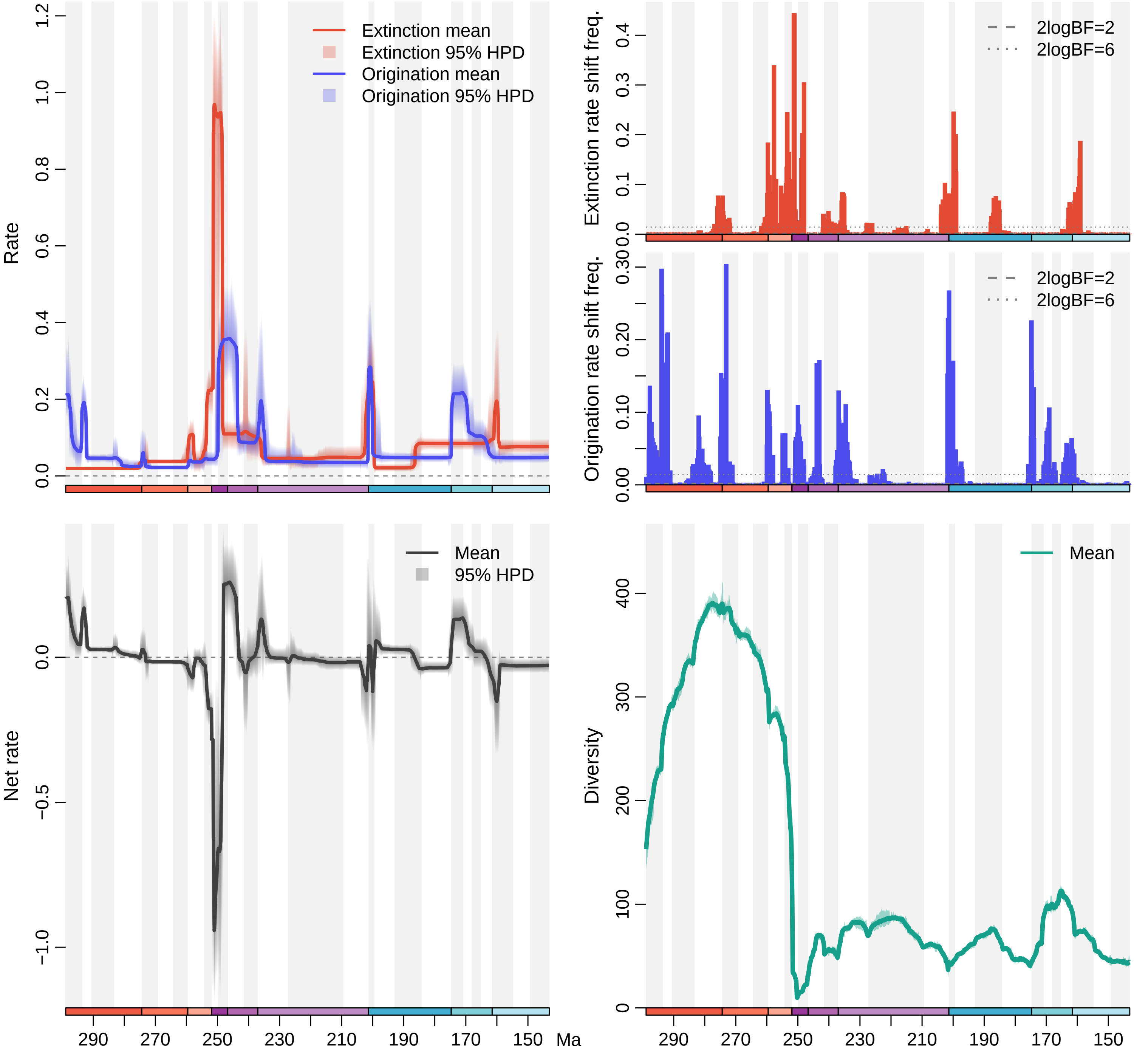

Supplementary Fig. 3. Estimated diversitification rates, rate shift frequencies, and diversity of Permian−Jurassic brachiopods.

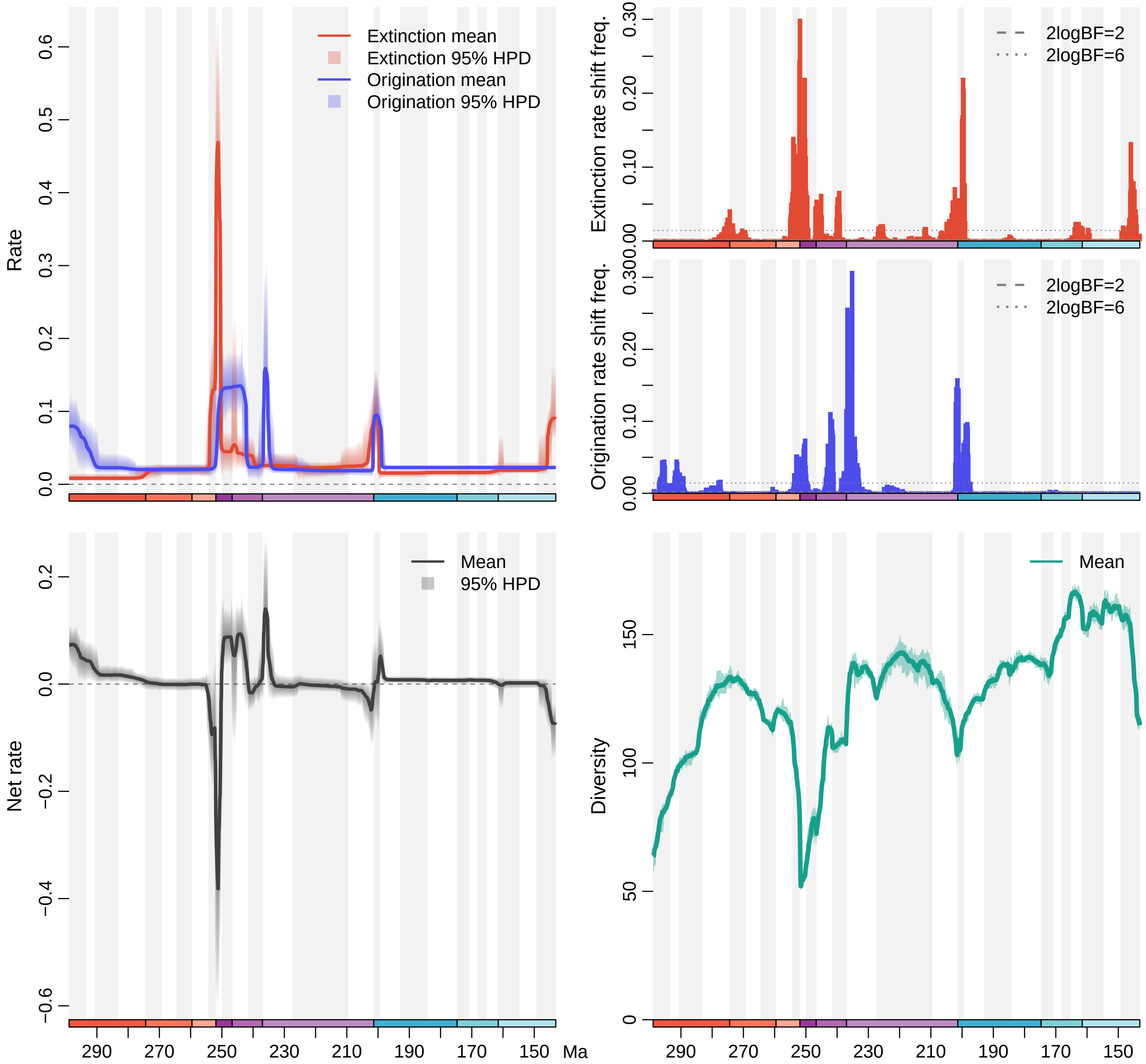

Supplementary Fig. 4. Estimated diversitification rates, rate shift frequencies, and diversity of Permian−Jurassic bivalves.

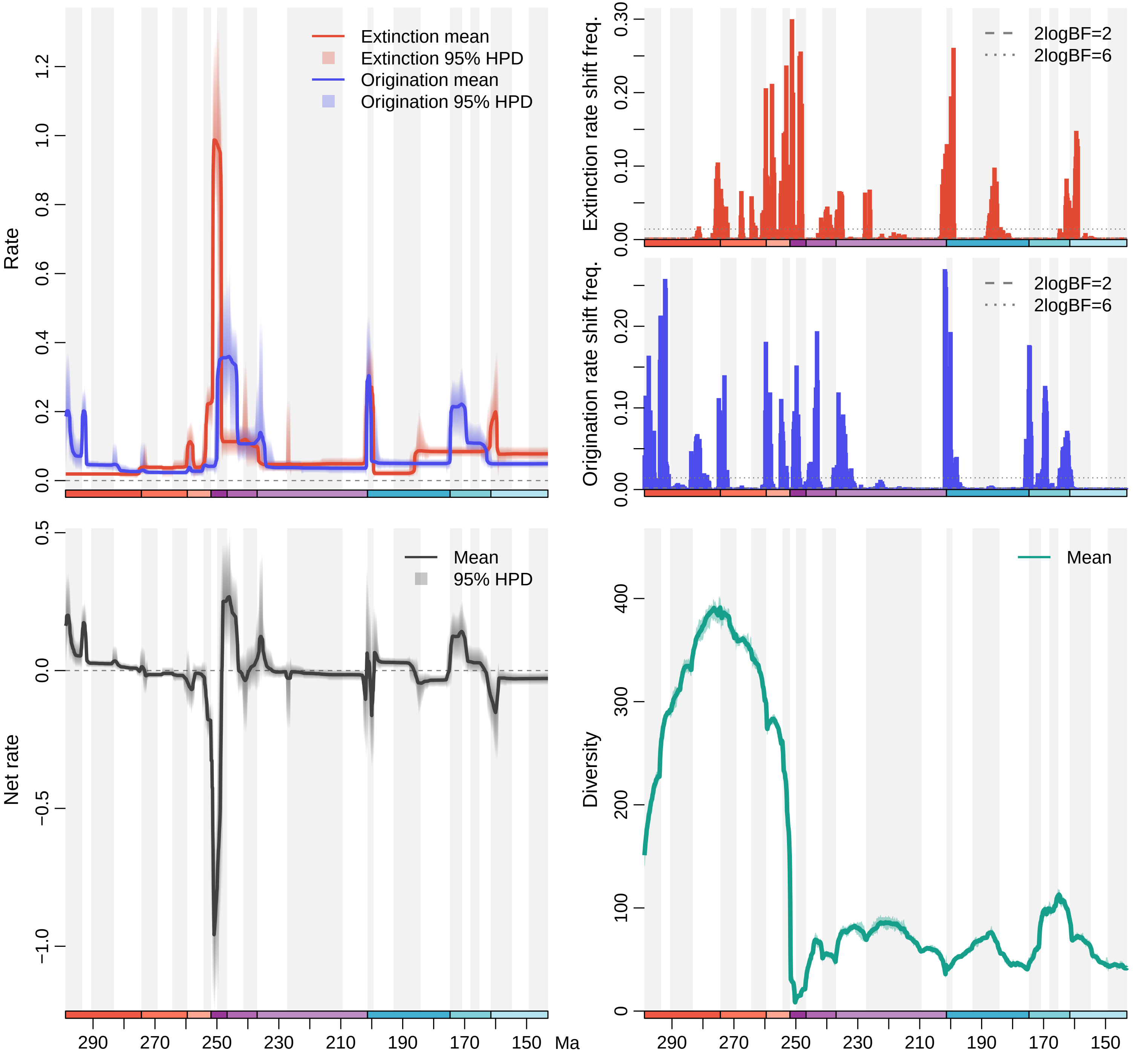

Supplementary Fig. 5. Estimated diversitification rates, rate shift frequencies, and diversity of epifaunal brachiopods.

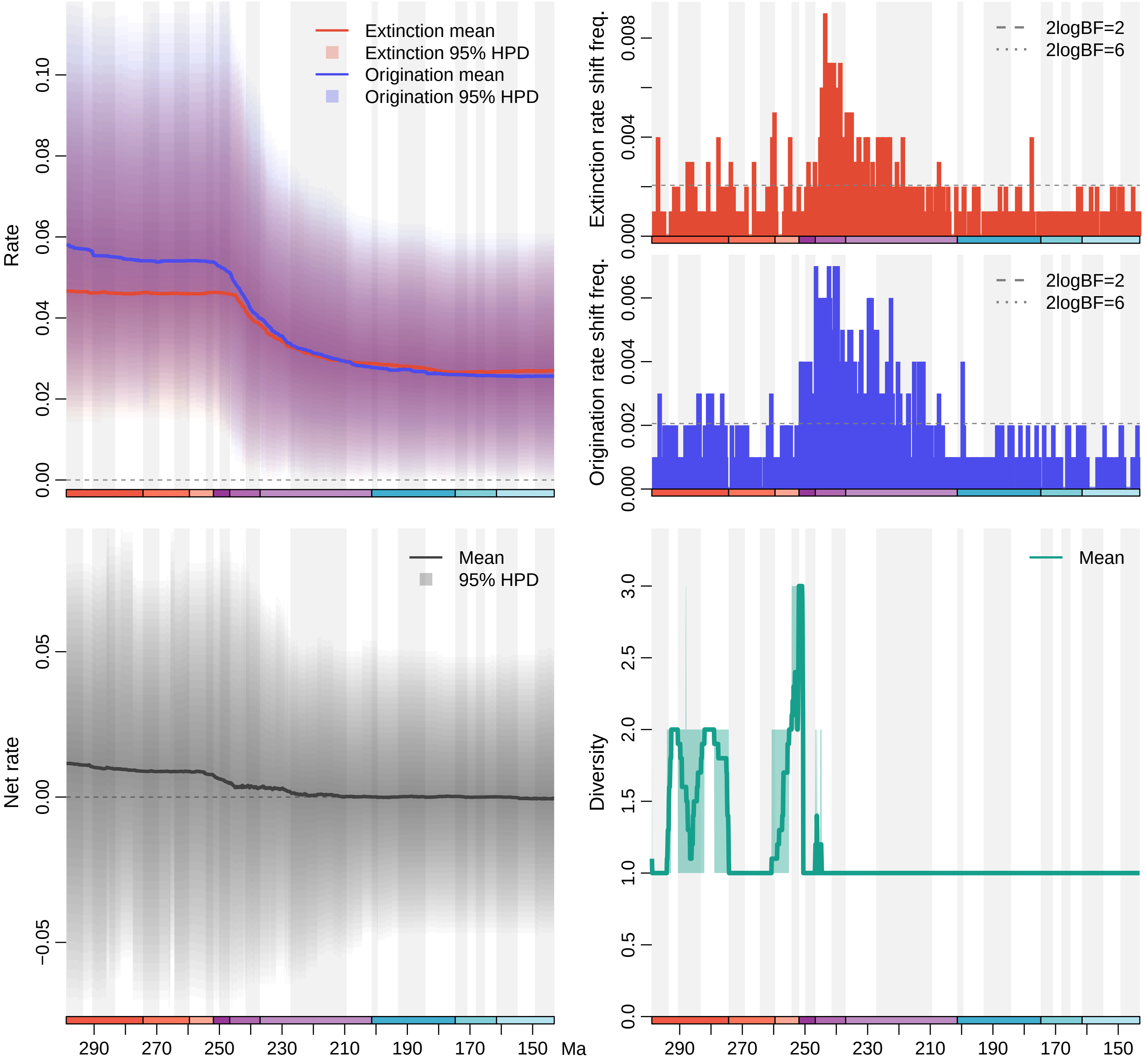

Supplementary Fig. 6. Estimated diversitification rates, rate shift frequencies, and diversity of infaunal brachiopods.

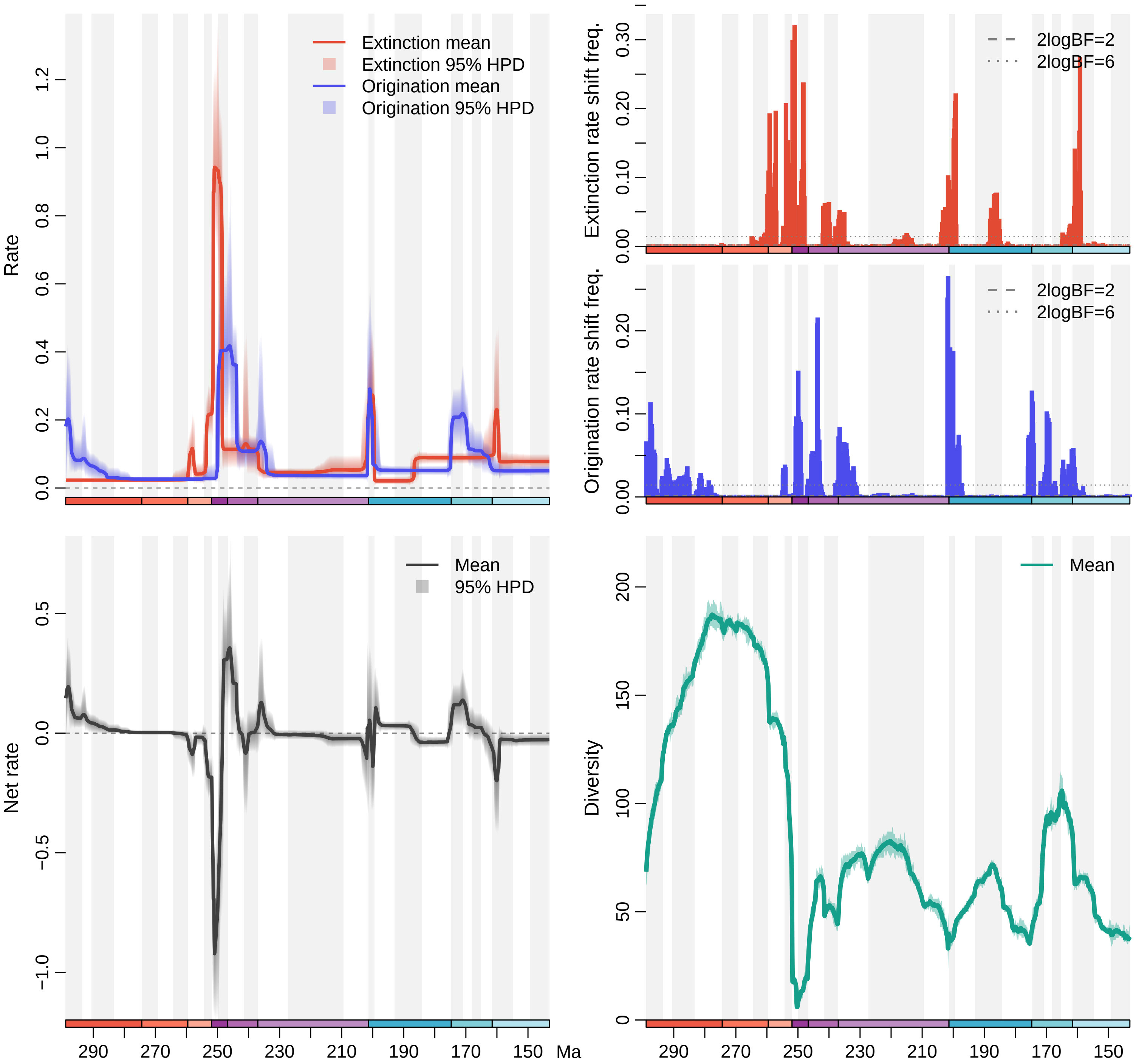

Supplementary Fig. 7. Estimated diversitification rates, rate shift frequencies, and diversity of pedicle−attached brachiopods.

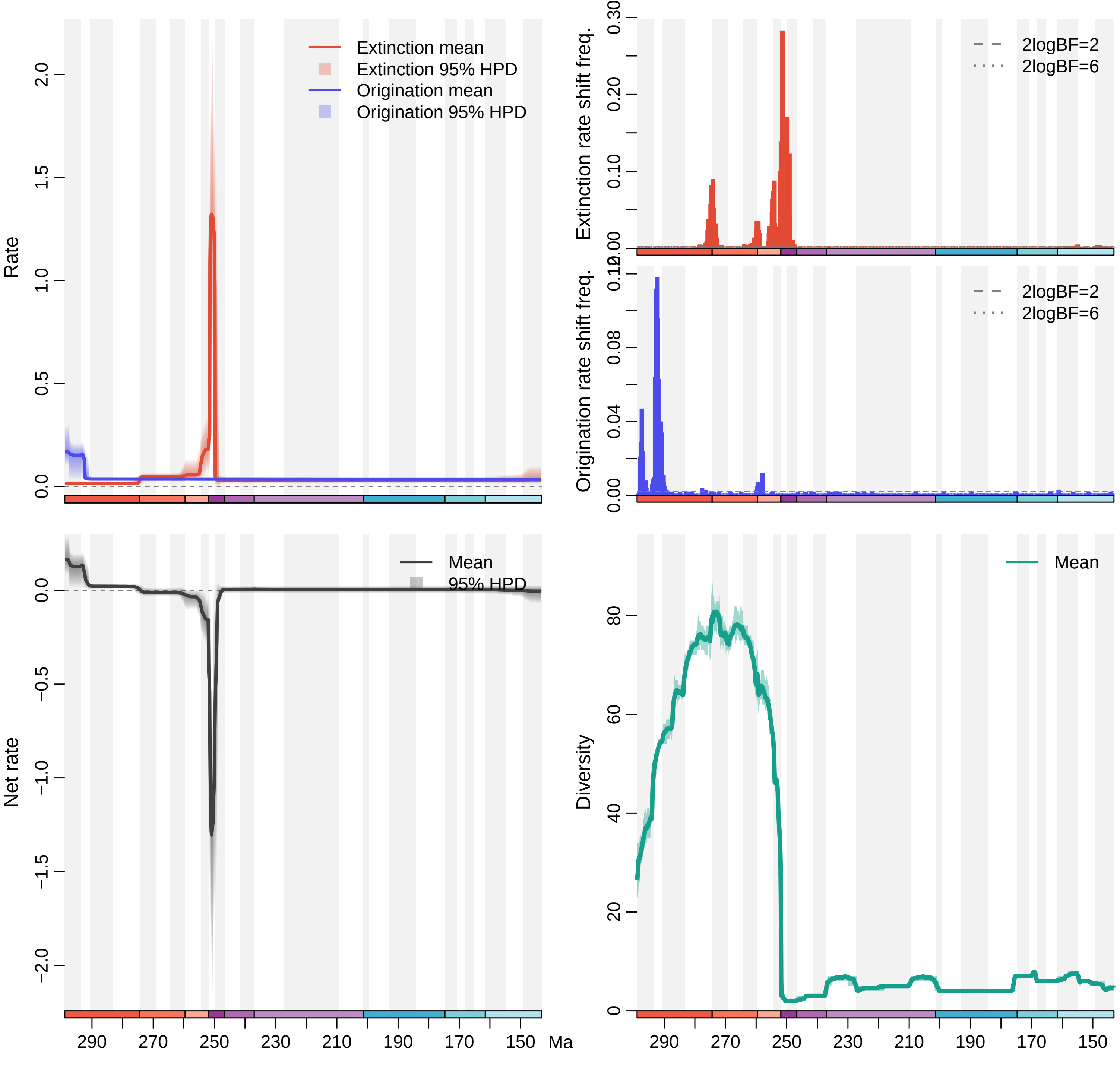

Supplementary Fig. 8. Estimated diversitification rates, rate shift frequencies, and diversity of cemented brachiopods.

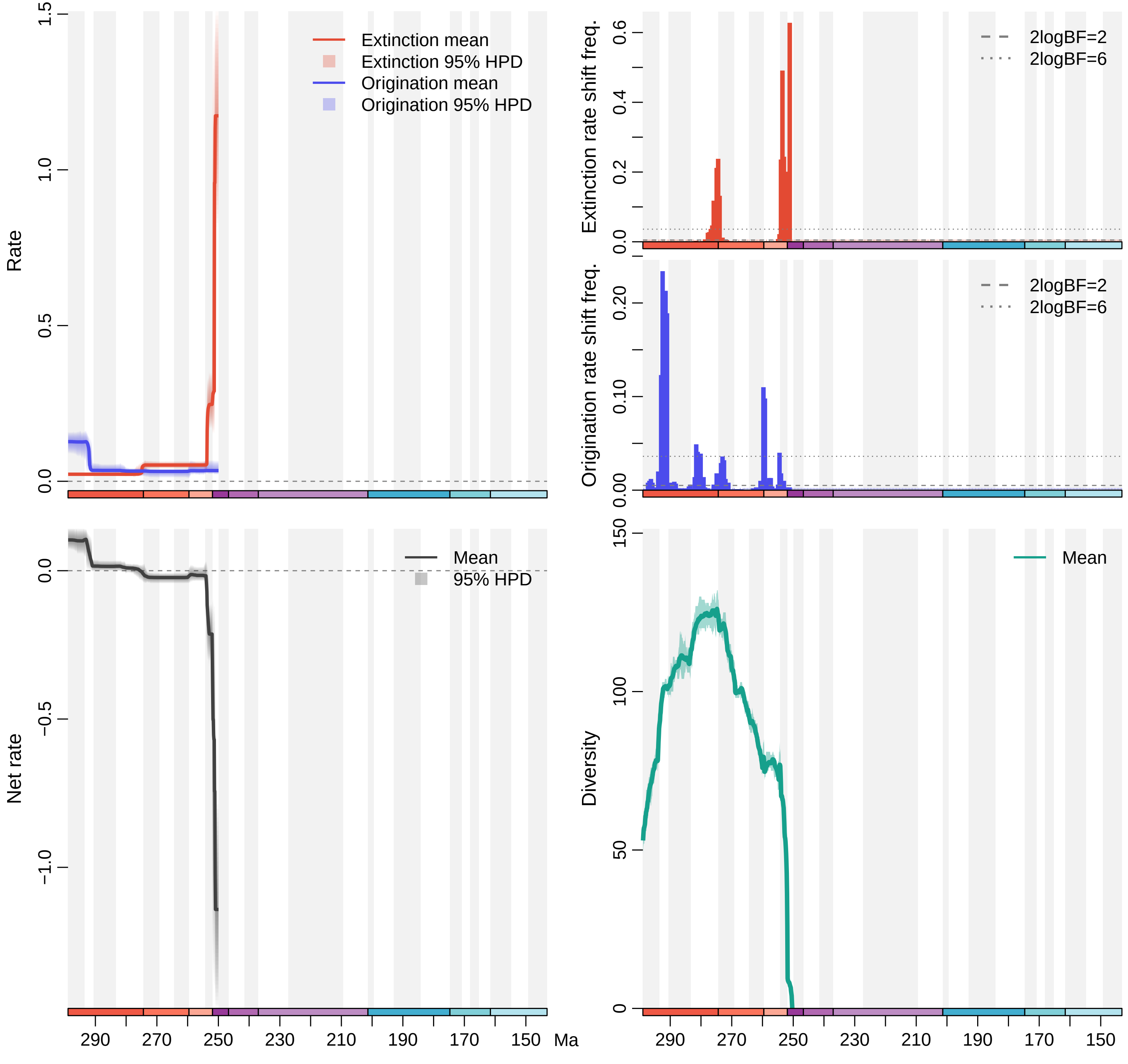

Supplementary Fig. 9. Estimated diversitification rates, rate shift frequencies, and diversity of reclining brachiopods.

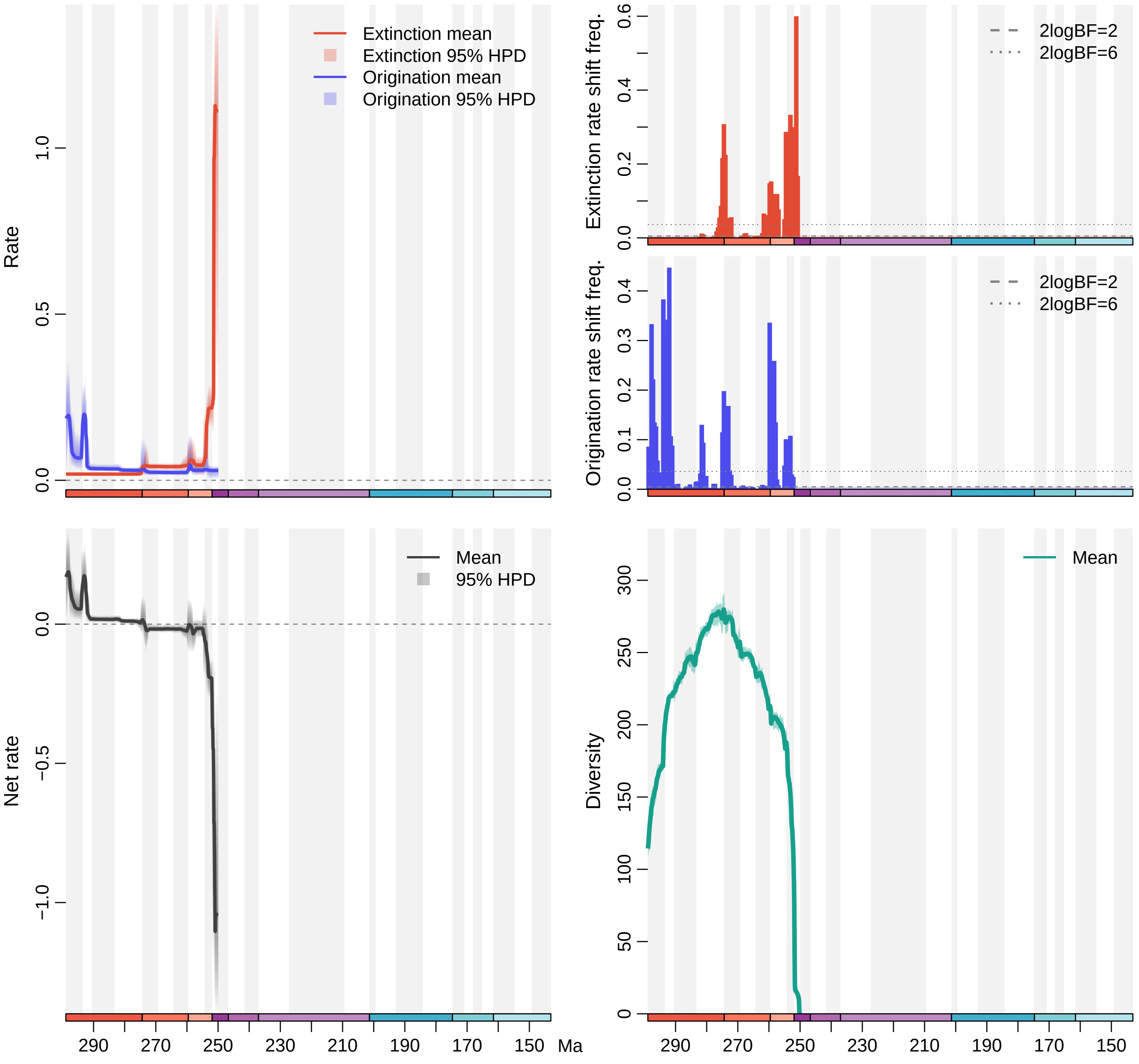

Supplementary Fig. 10. Estimated diversitification rates, rate shift frequencies, and diversity of brachiopod orders extinct in the P−Tr mass extinction.

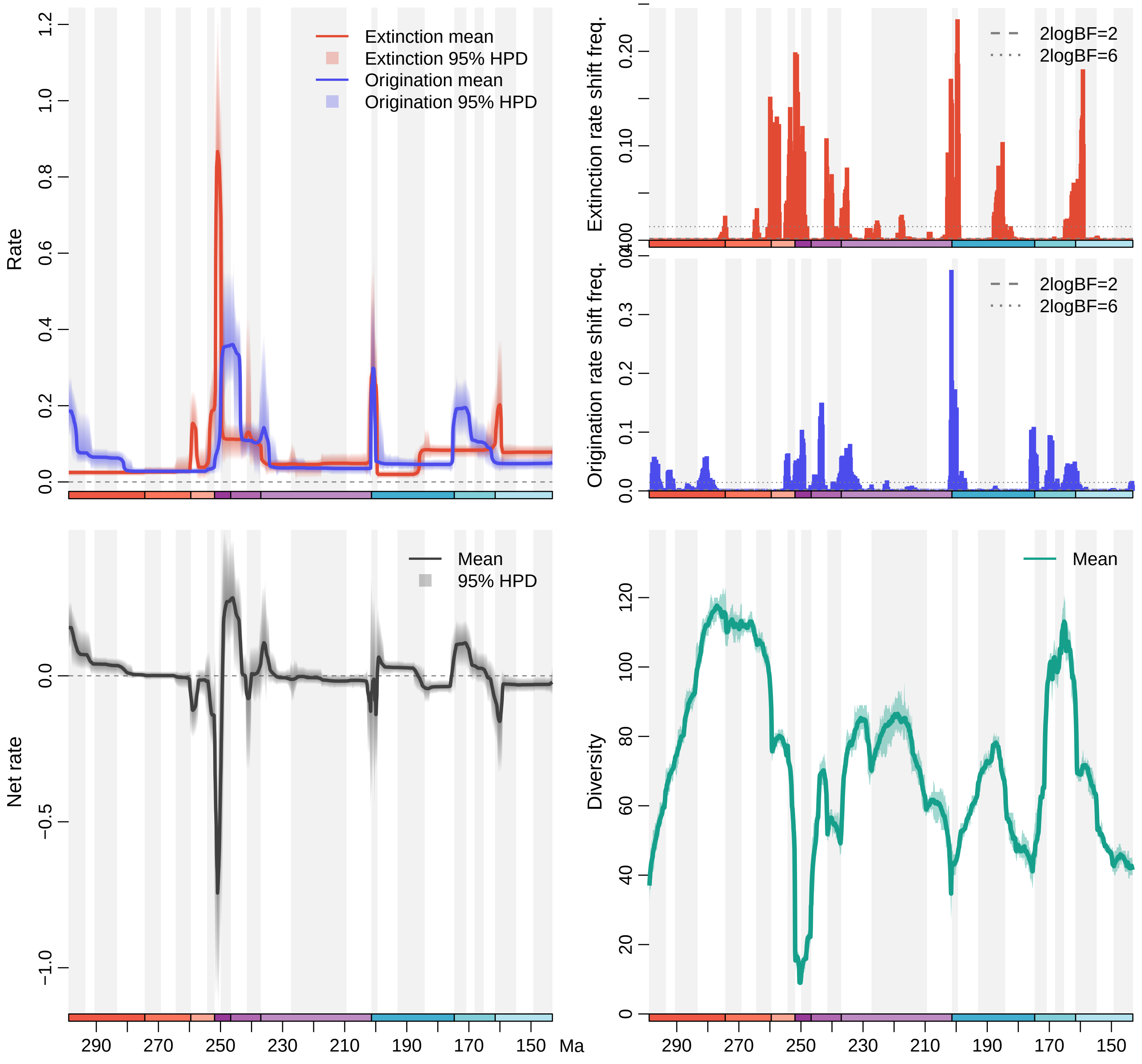

Supplementary Fig. 11. Estimated diversitification rates, rate shift frequencies, and diversity of brachiopod orders survived or originated after the P−Tr mass extinction.

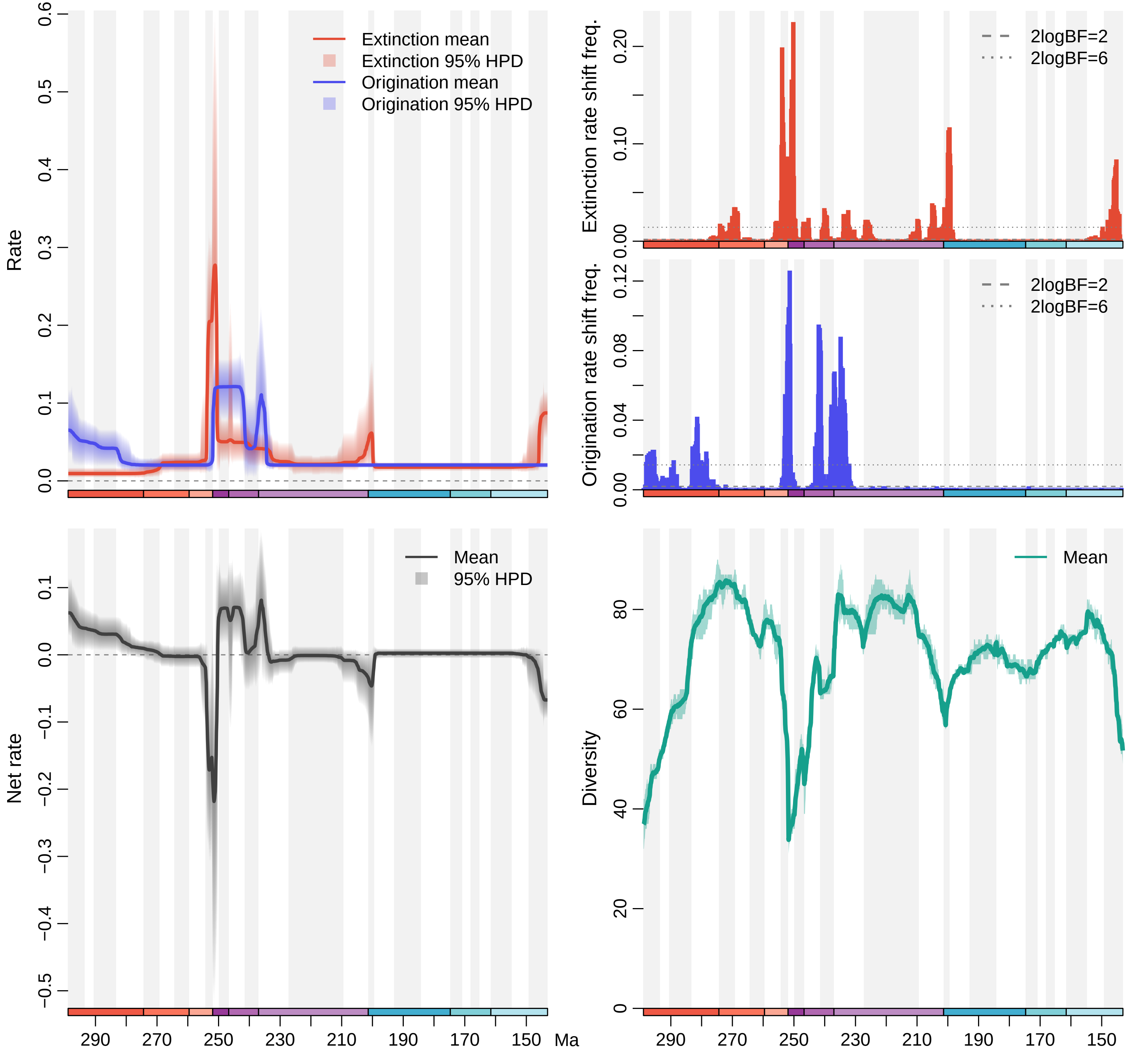

Supplementary Fig. 12. Estimated diversitification rates, rate shift frequencies, and diversity of epifaunal bivalves.

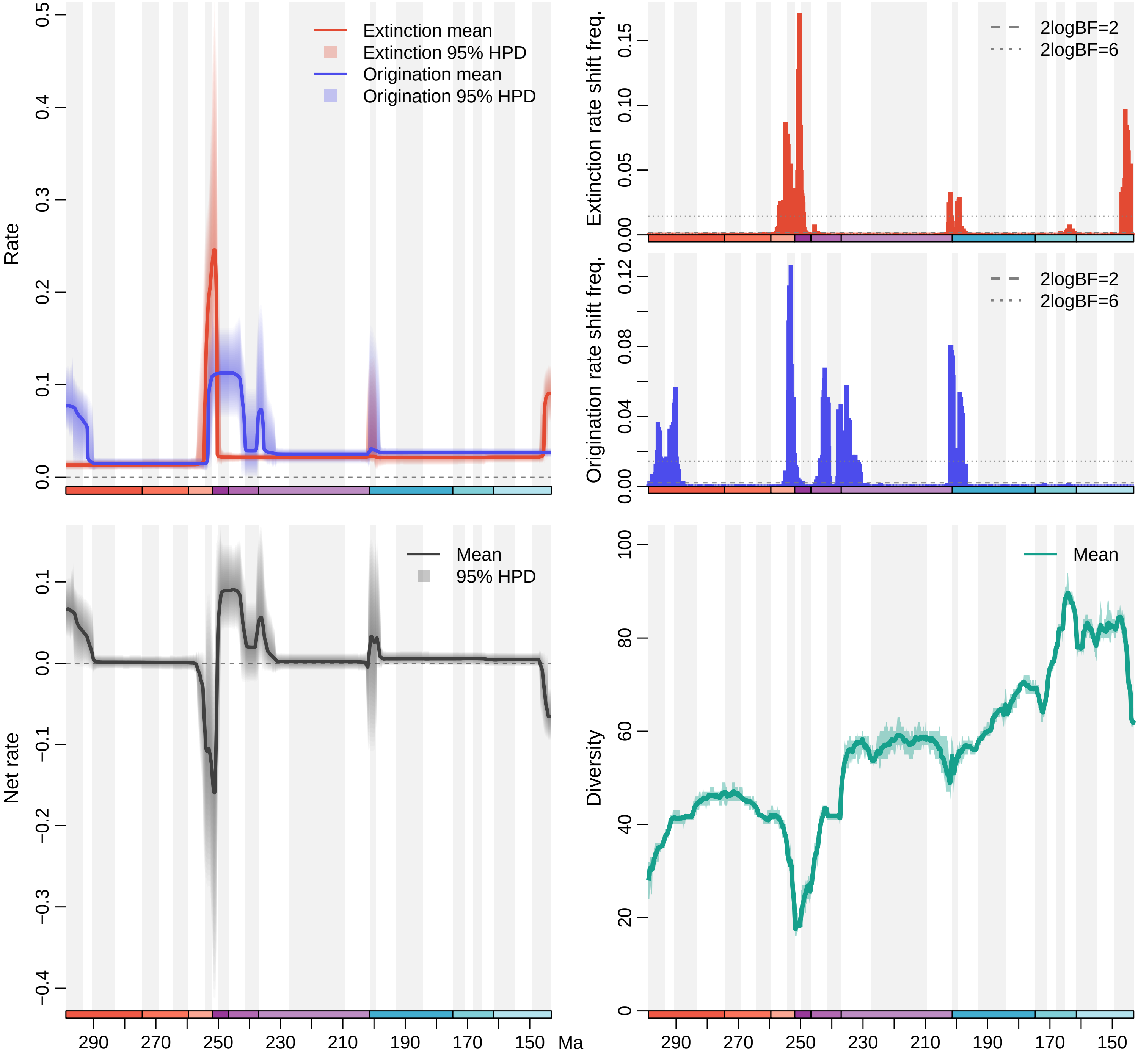

Supplementary Fig. 13. Estimated diversititification rates, rate shift frequencies, and diversity of infaunal bivalves.

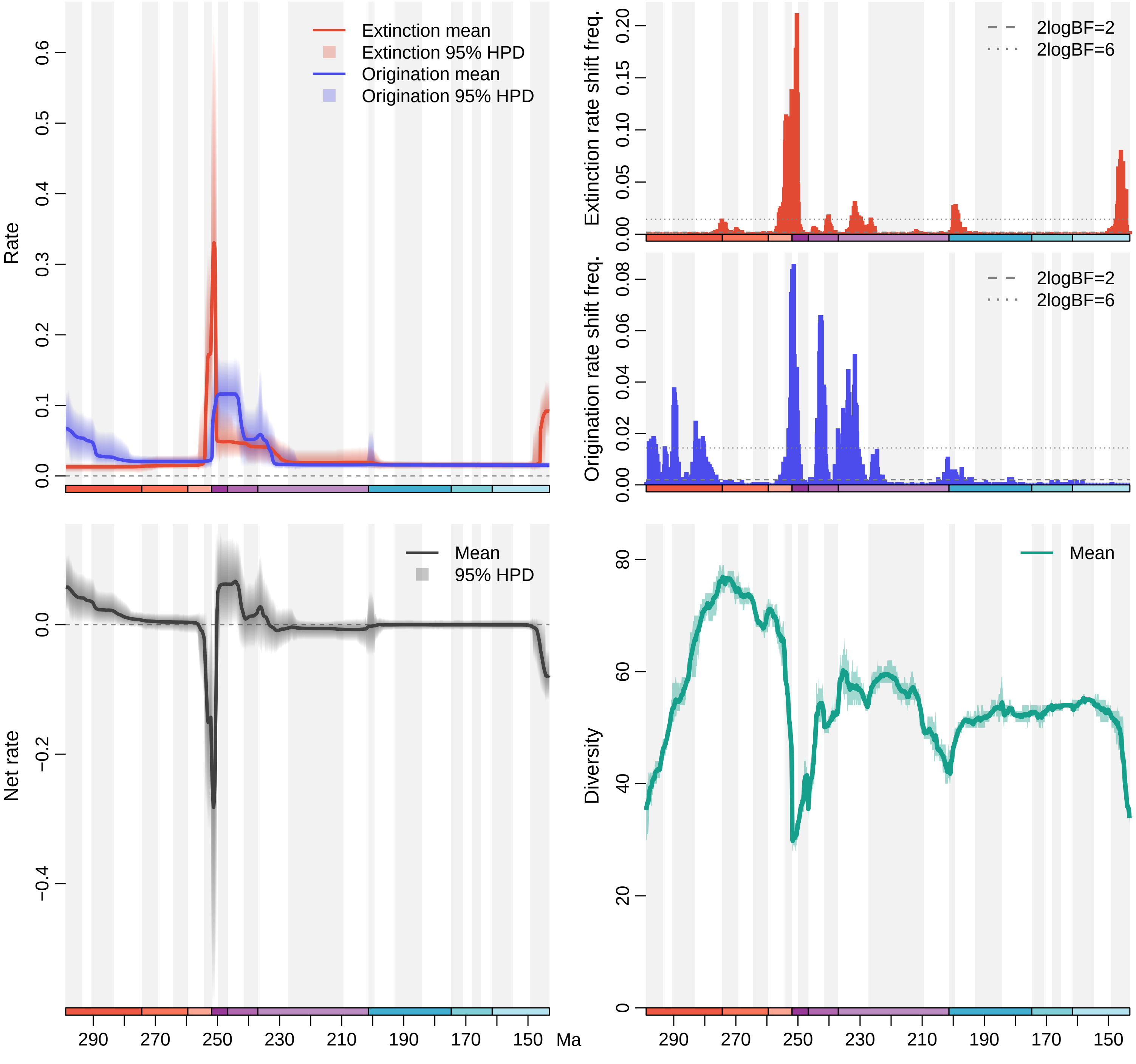

Supplementary Fig. 14. Estimated diversitification rates, rate shift frequencies, and diversity of epibyssate bivalves.

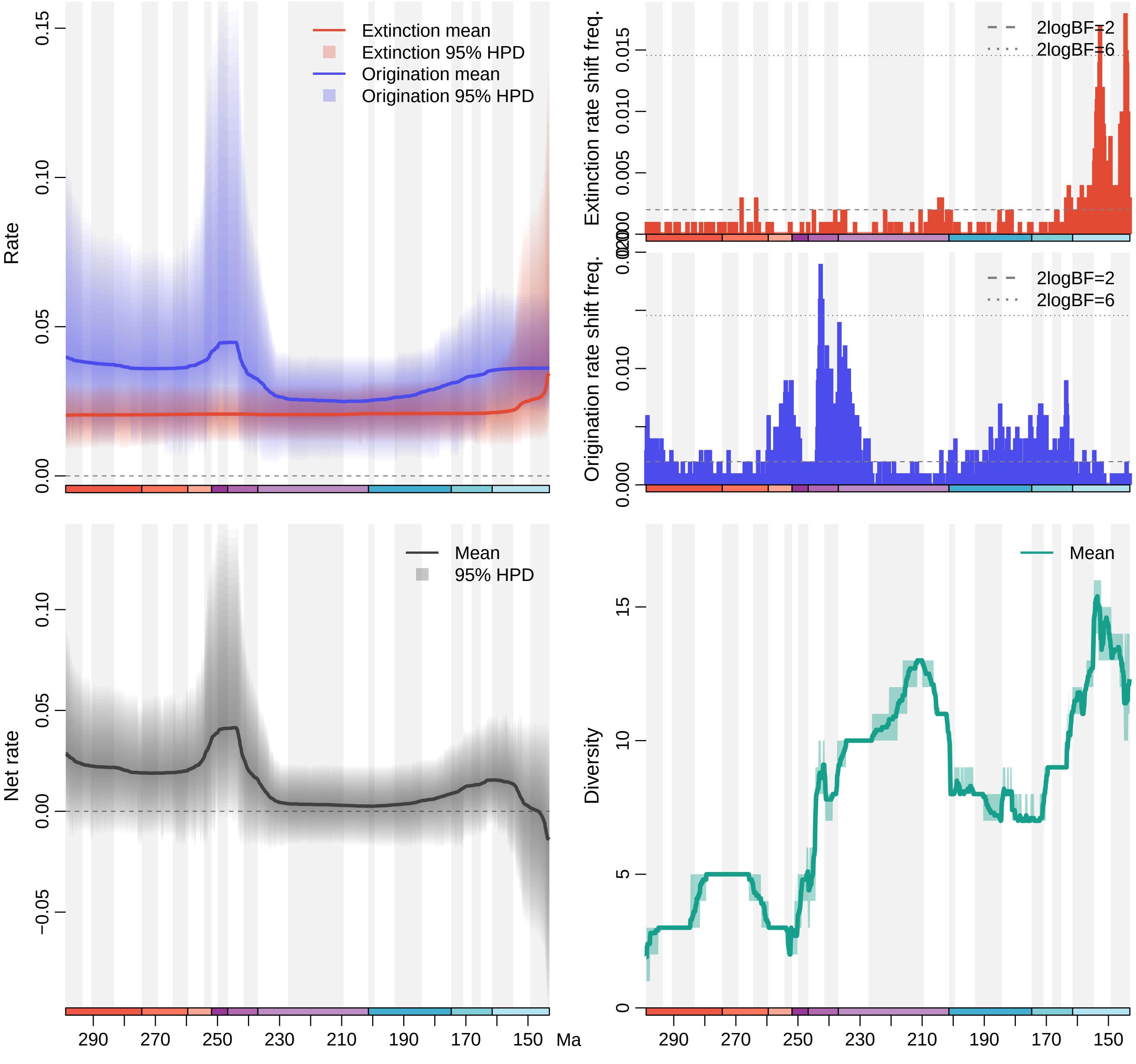

Supplementary Fig. 15. Estimated diversitification rates, rate shift frequencies, and diversity of cemented bivalves.

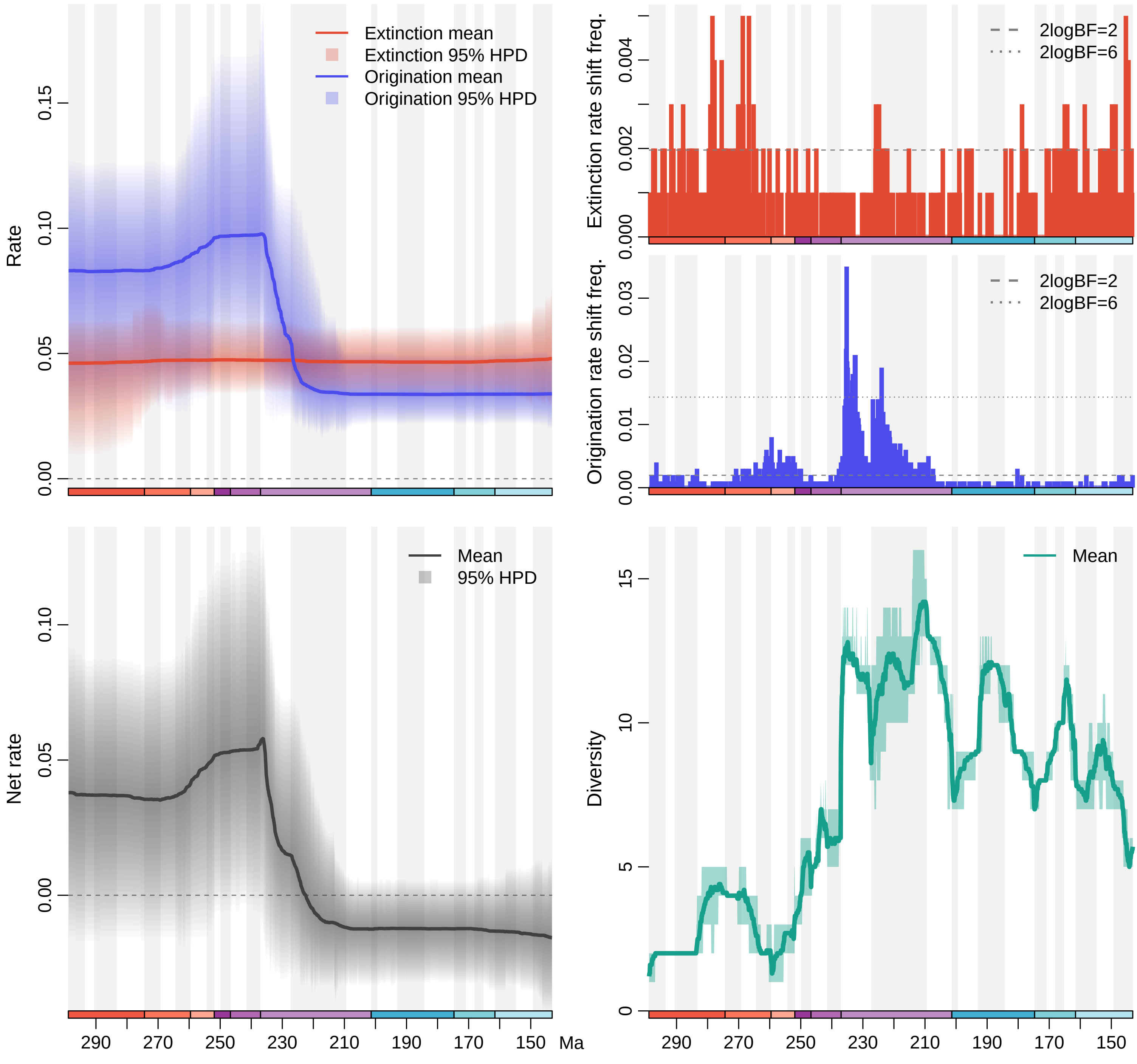

Supplementary Fig. 16. Estimated diversititification rates, rate shift frequencies, and diversity of reclining bivalves.

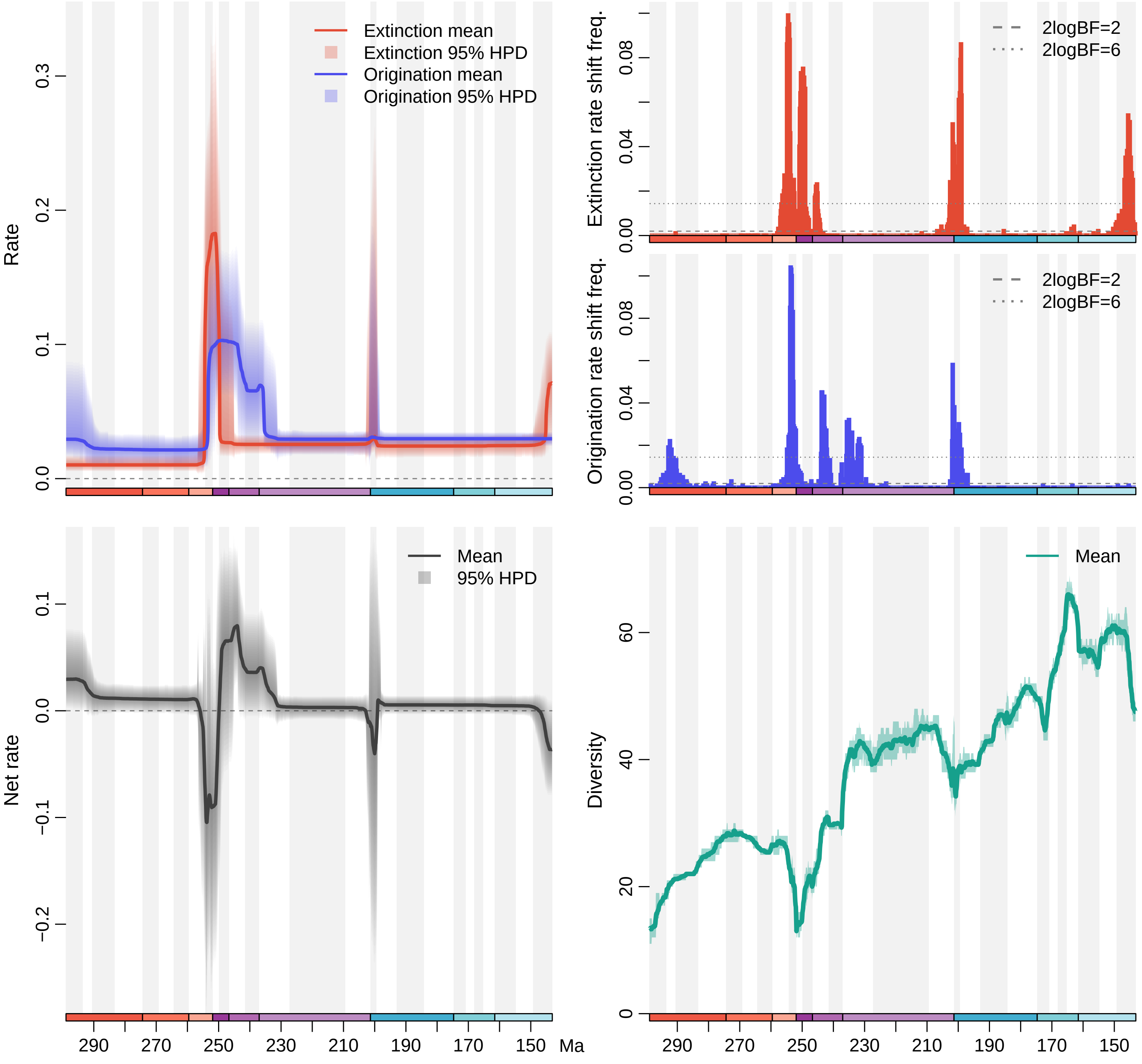

Supplementary Fig. 17. Estimated diversitification rates, rate shift frequencies, and diversity of shallow infaunal bivalves.

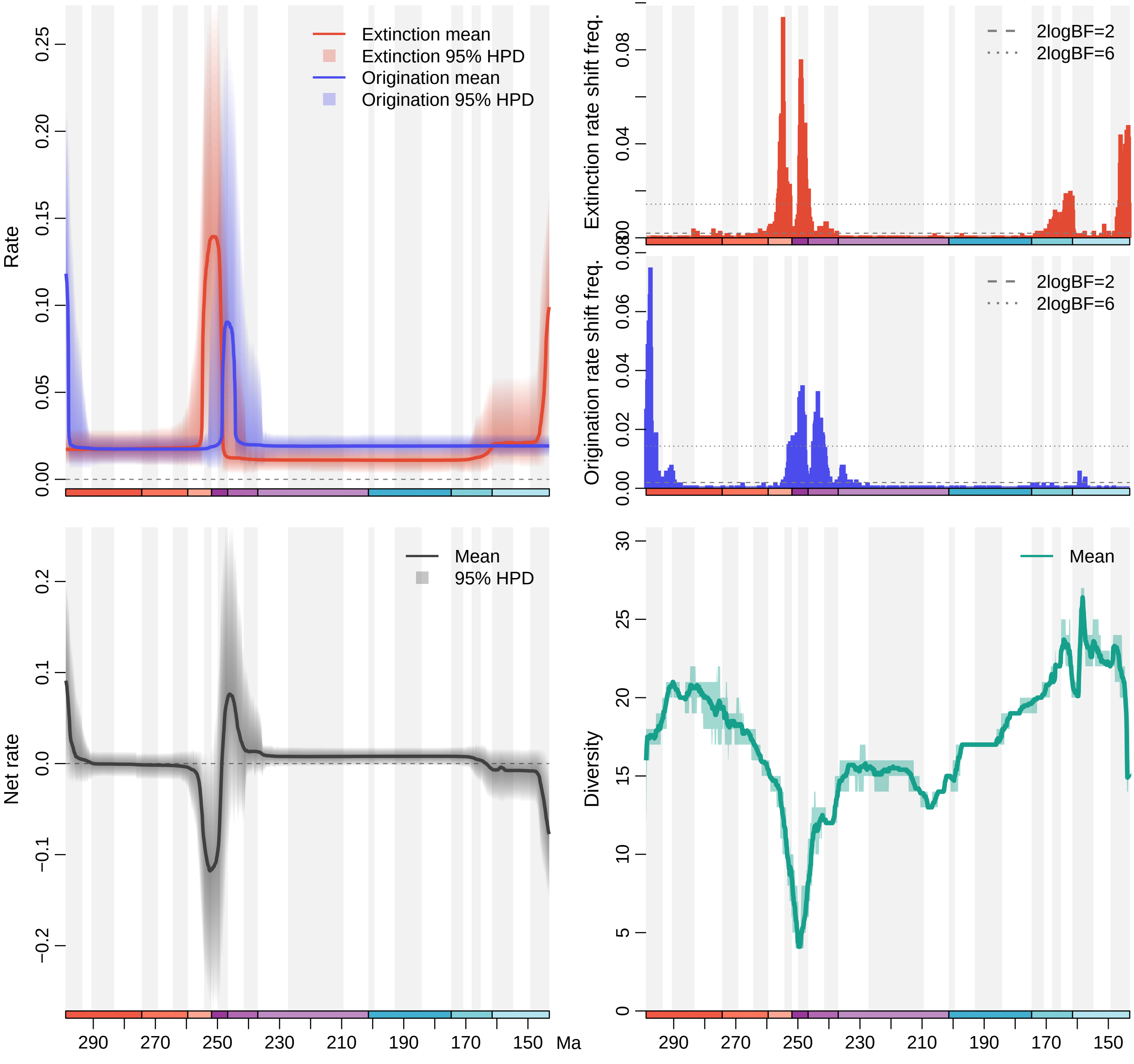

Supplementary Fig. 18. Estimated diversitification rates, rate shift frequencies, and diversity of deep infaunal bivalves.

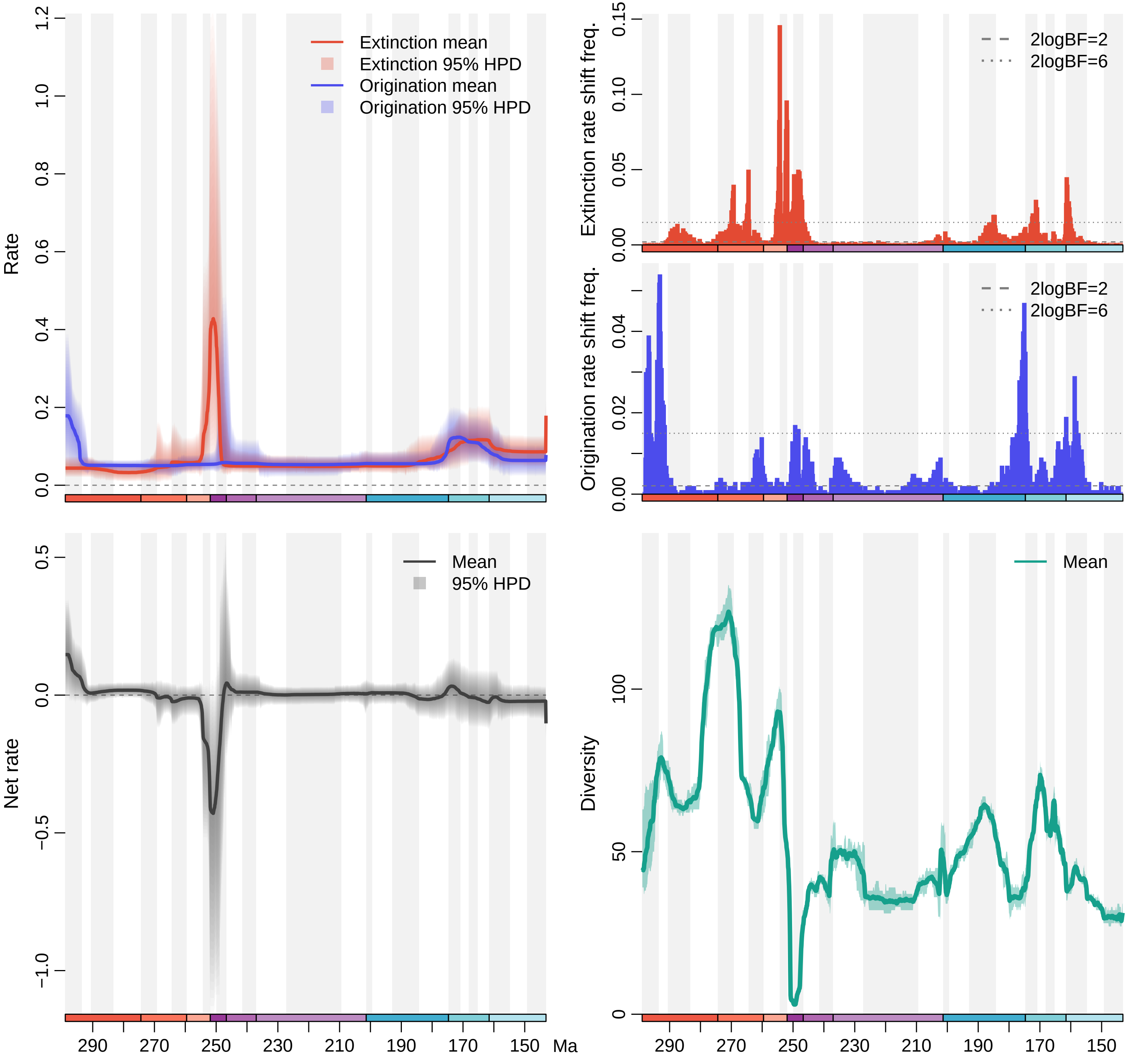

Supplementary Fig. 19. Estimated diversitification rates, rate shift frequencies, and diversity of north−western Tethyan brachiopods.

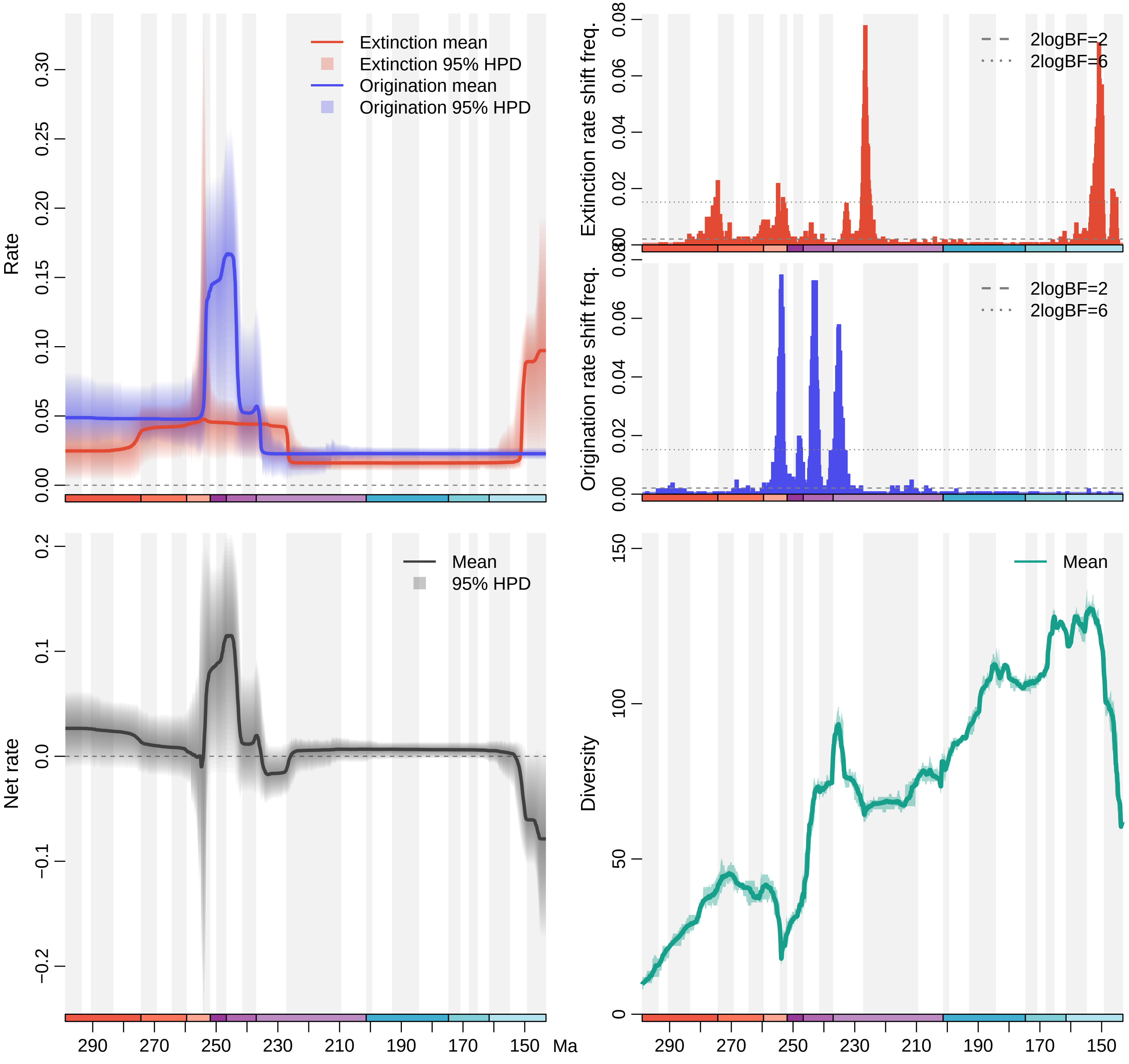

Supplementary Fig. 20. Estimated diversititification rates, rate shift frequencies, and diversity of north−western Tethyan bivalves.

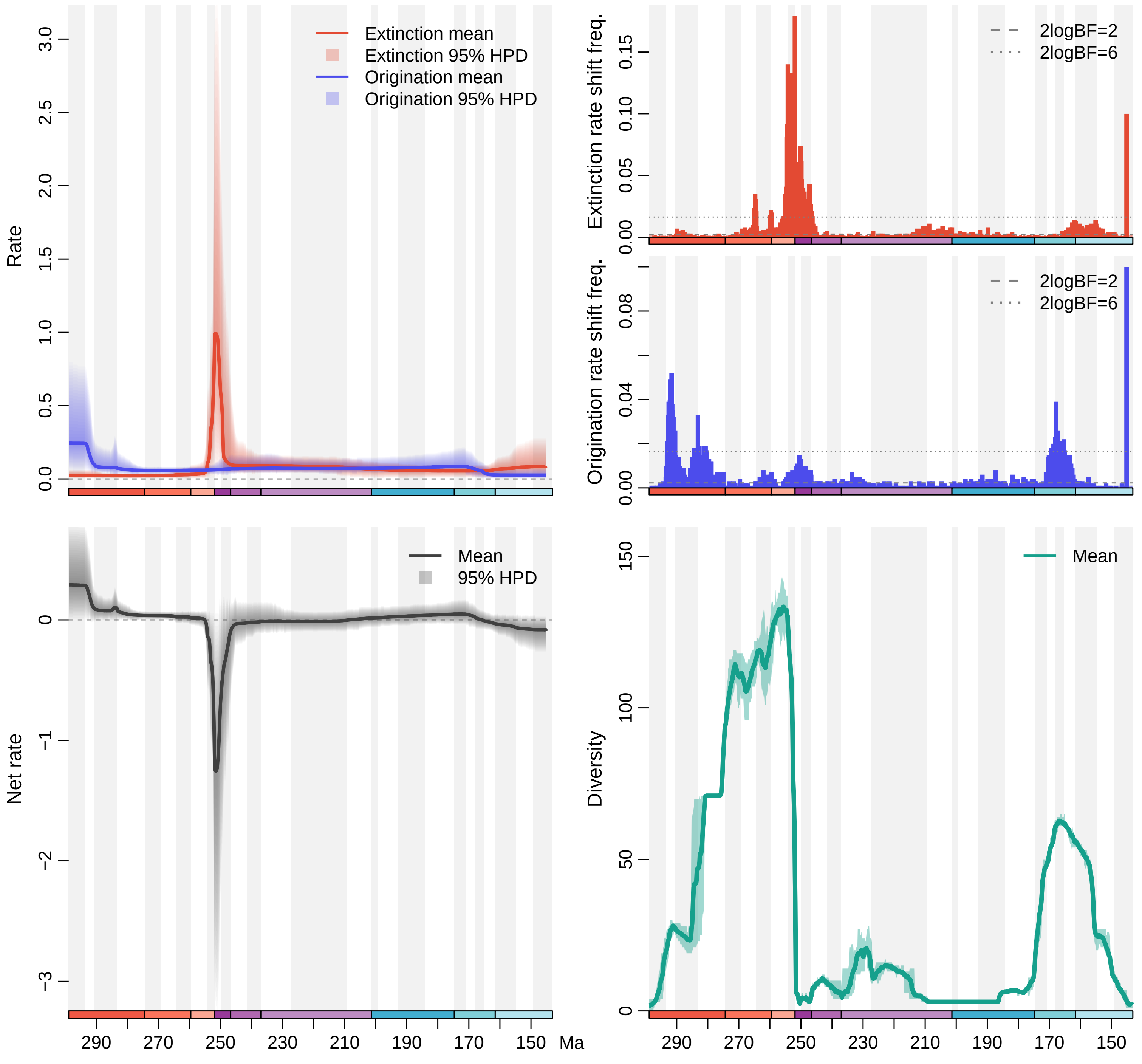

Supplementary Fig. 21. Estimated diversitification rates, rate shift frequencies, and diversity of south−western Tethyan brachiopods.

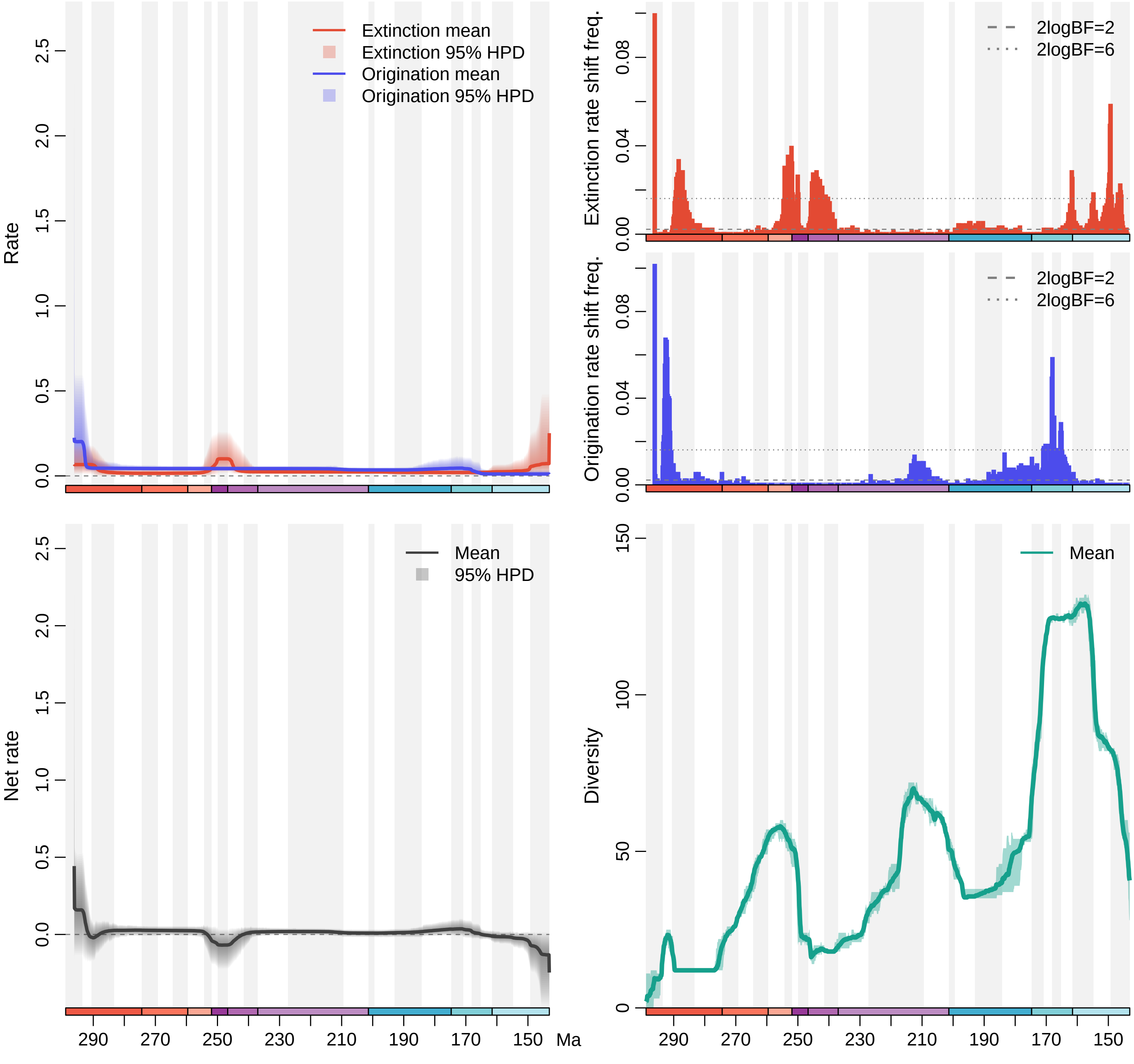

Supplementary Fig. 22. Estimated diversitification rates, rate shift frequencies, and diversity of south−western Tethyan bivalves.

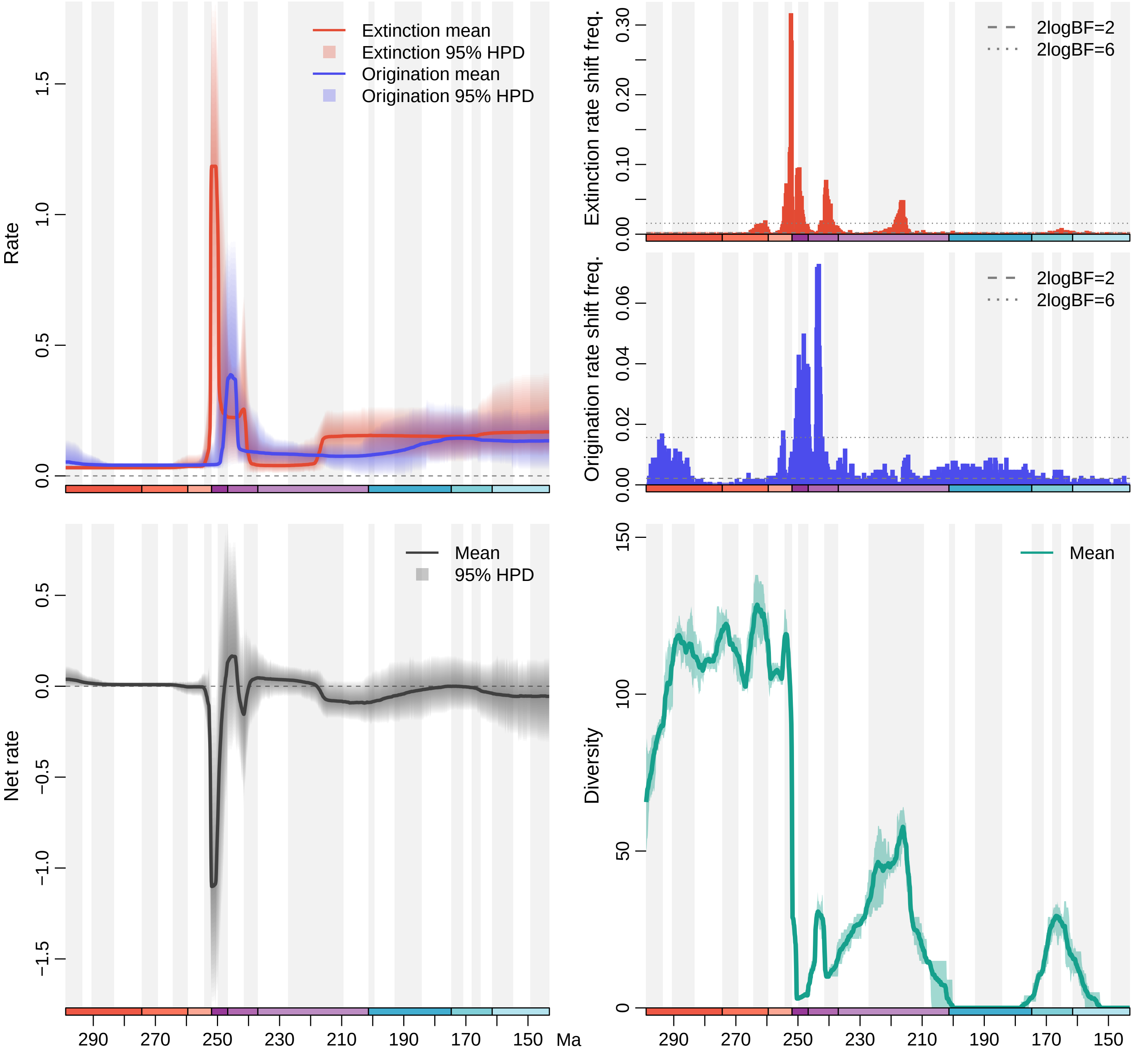

Supplementary Fig. 23. Estimated diversititification rates, rate shift frequencies, and diversity of eastern Tethyan brachiopods.

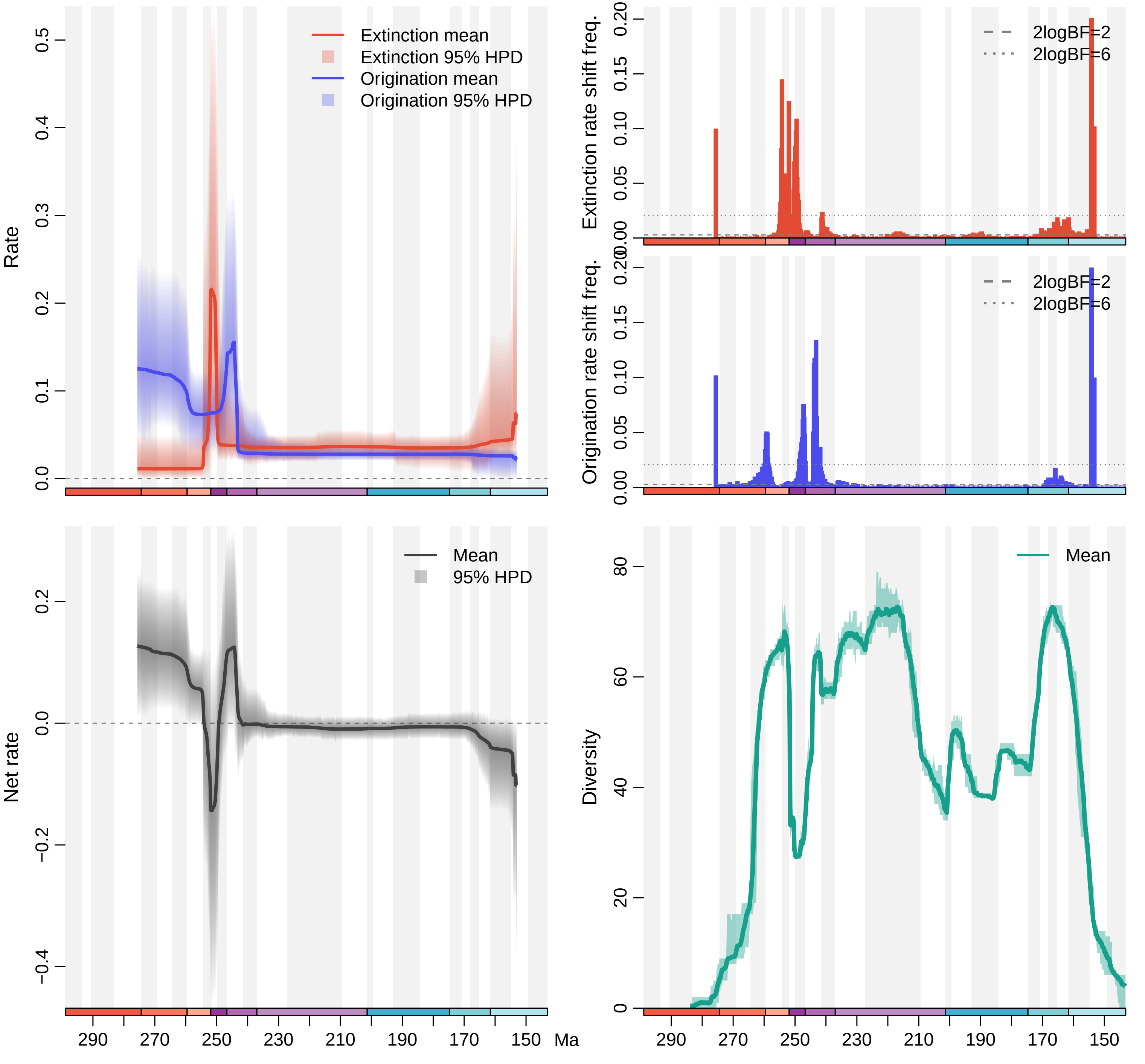

Supplementary Fig. 24. Estimated diversitification rates, rate shift frequencies, and diversity of eastern Tethyan bivalves.

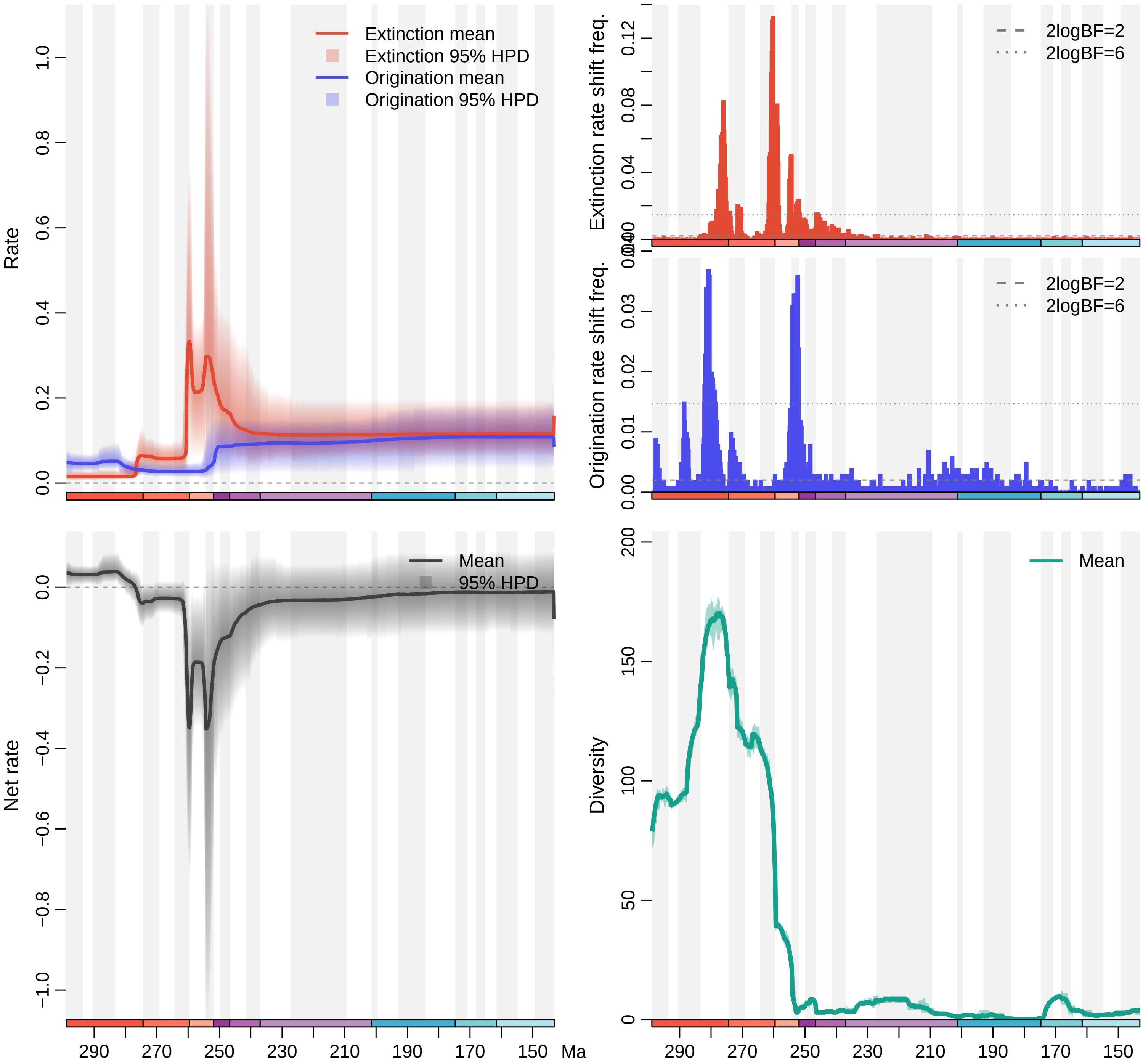

Supplementary Fig. 25. Estimated diversitification rates, rate shift frequencies, and diversity of northern Panthalassic brachiopods.

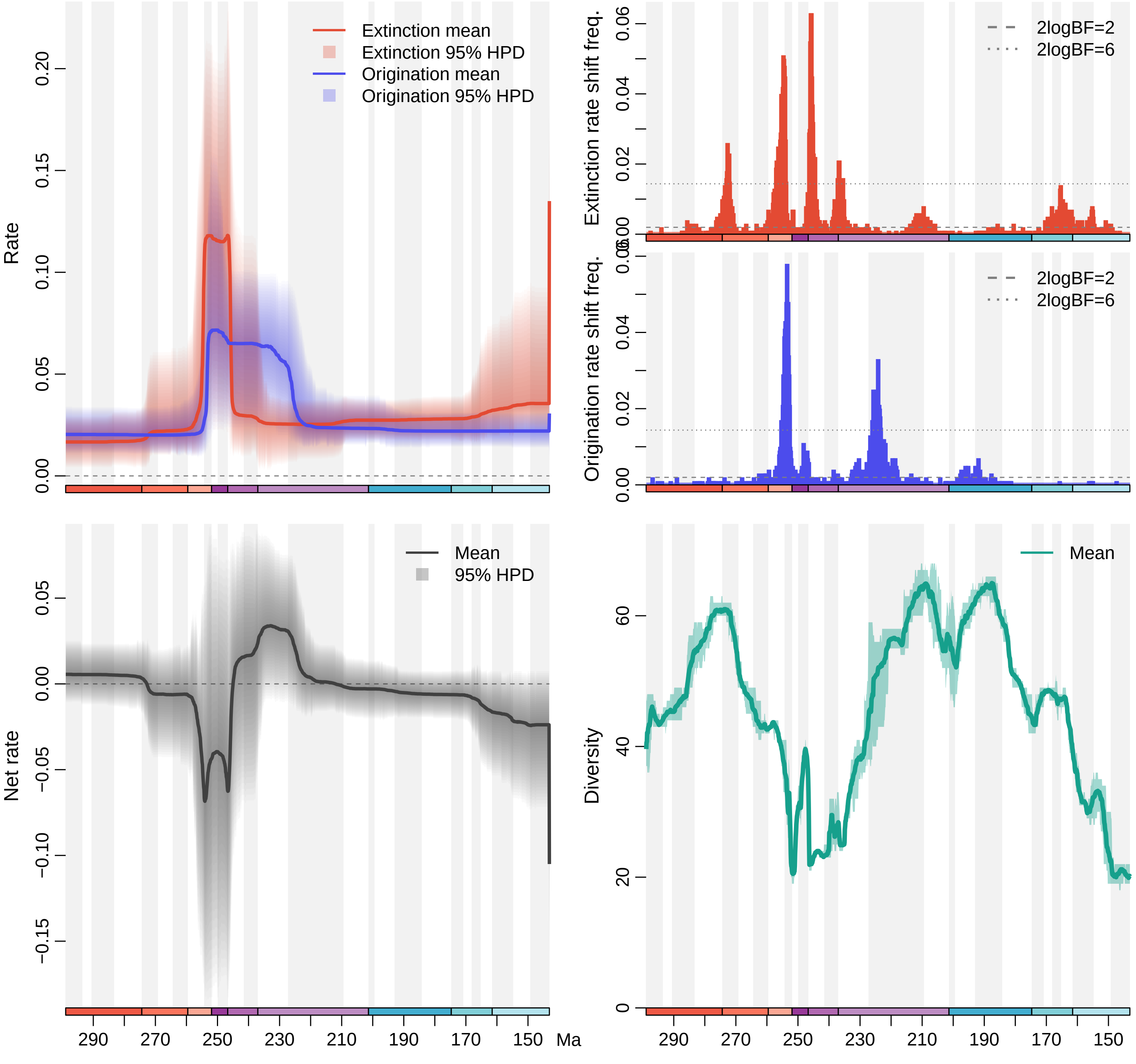

Supplementary Fig. 26. Estimated diversitification rates, rate shift frequencies, and diversity of northern Panthalassic bivalves.

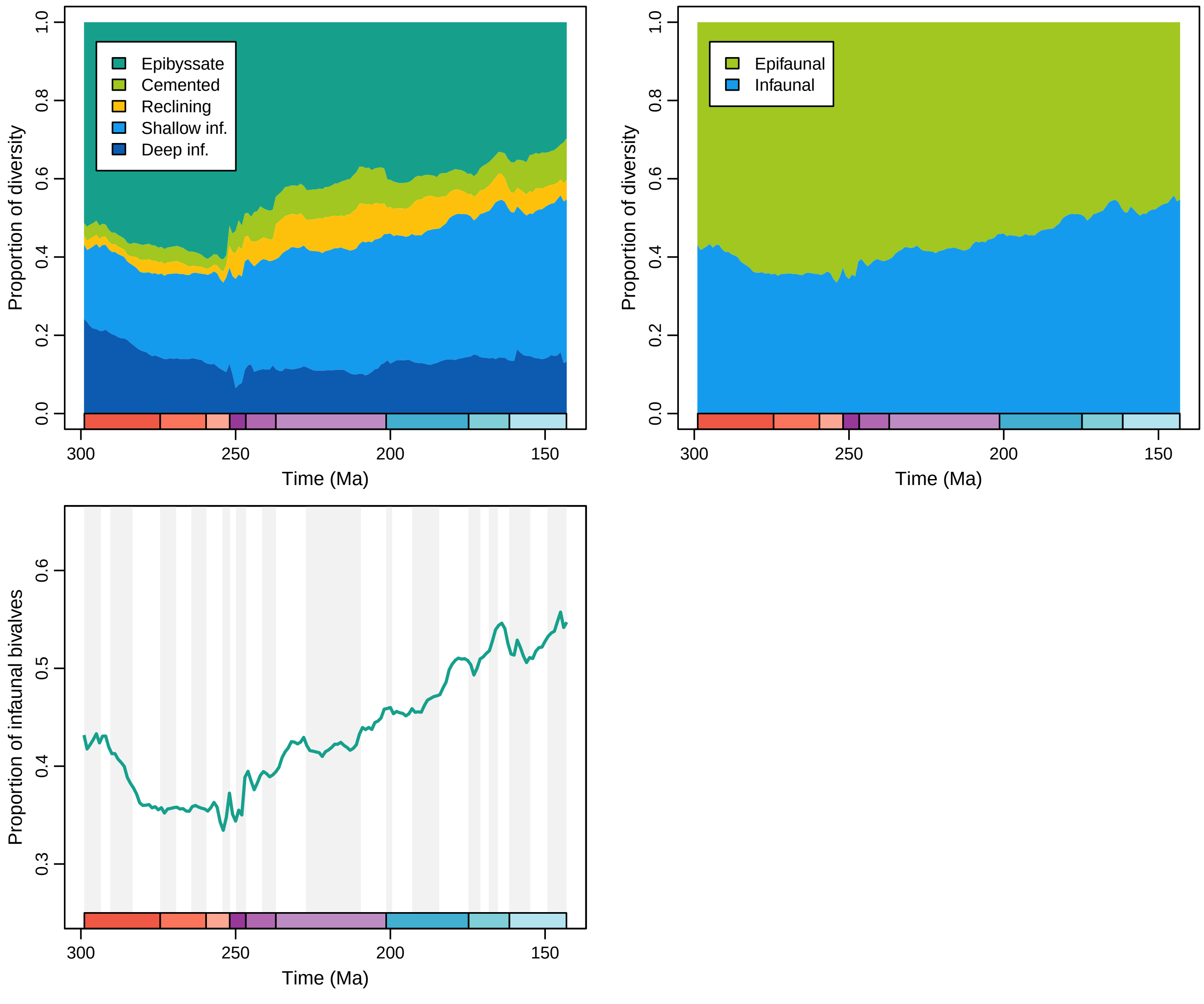

Supplementary Fig. 27. Proportion of bivalve diversity of various ecological types.

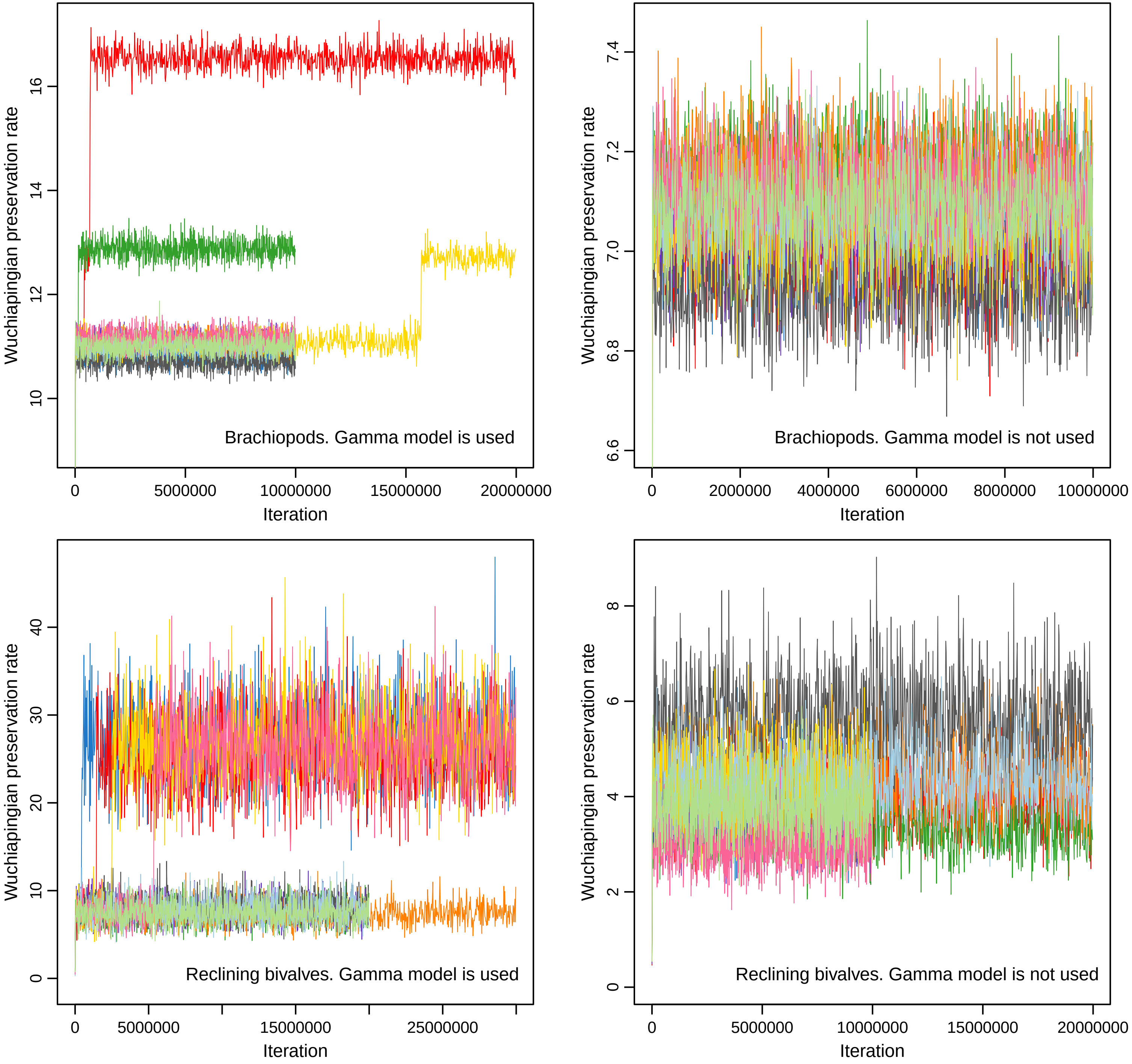

Supplementary Fig. 28. Trace plots of PyRate analyses with or without the Gamma model.
The Wuchiapingian preservation rate is selected to show the difference.

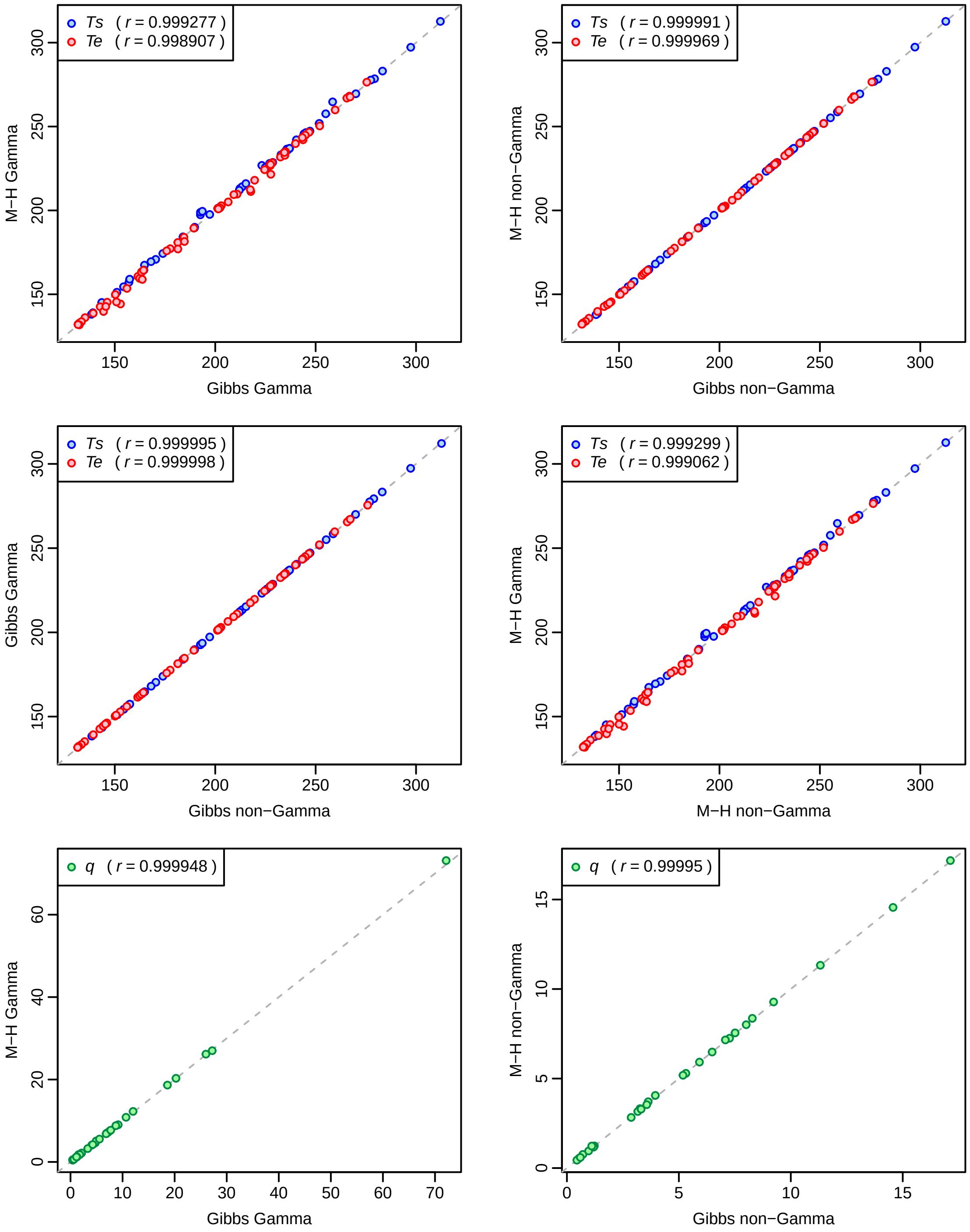

Supplementary Fig. 29. Comparison of parameters estimated by different algorithms (M−H vs. Gibbs, Gamma vs. non−Gamma).All correlations are significant ($p < 0.01$). The dataset analyzed is the reclining bivalve dataset. Ts and Te, time of origination and extinction; q, preservation rate.

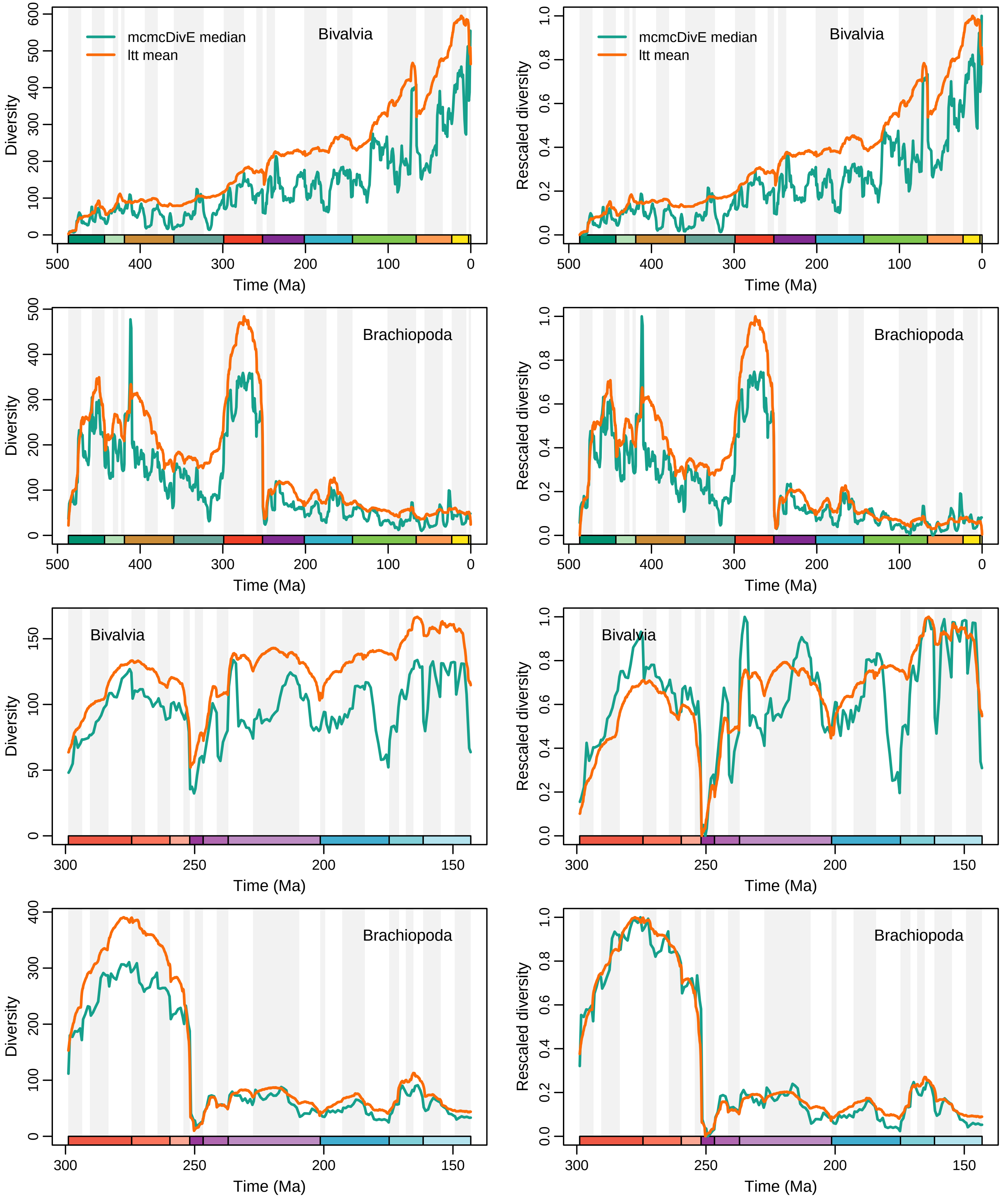

Supplementary Fig. 30. Comparison of diversity estimated by mcmcDivE and ltt.
The left column shows raw result; the right column shows diversity rescaled to 0−1.

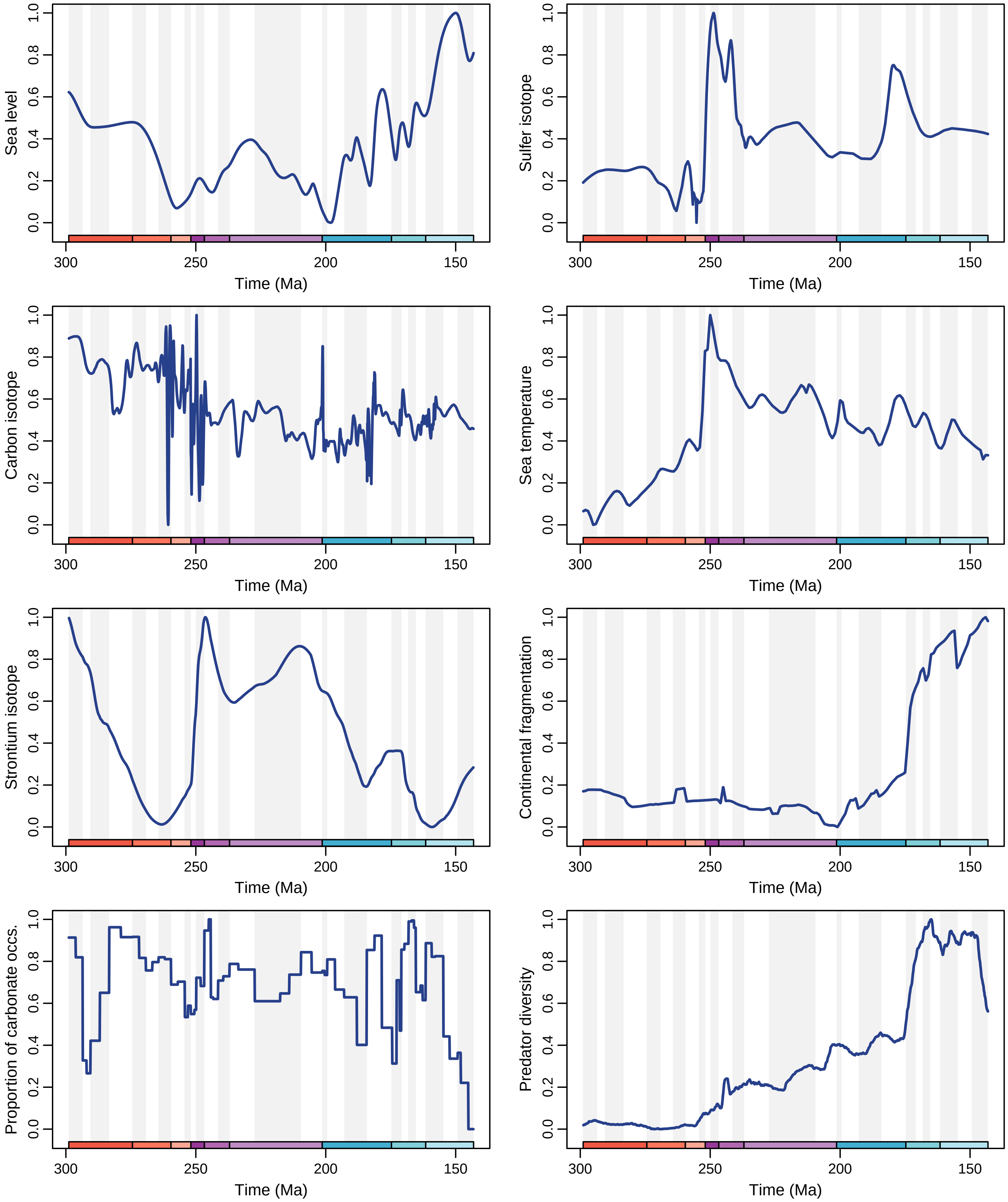

Supplementary Fig. 31. Factors included in the MBD analyses (except the brachiopod and bivalve diversities). All factors are rescaled to 0−1.

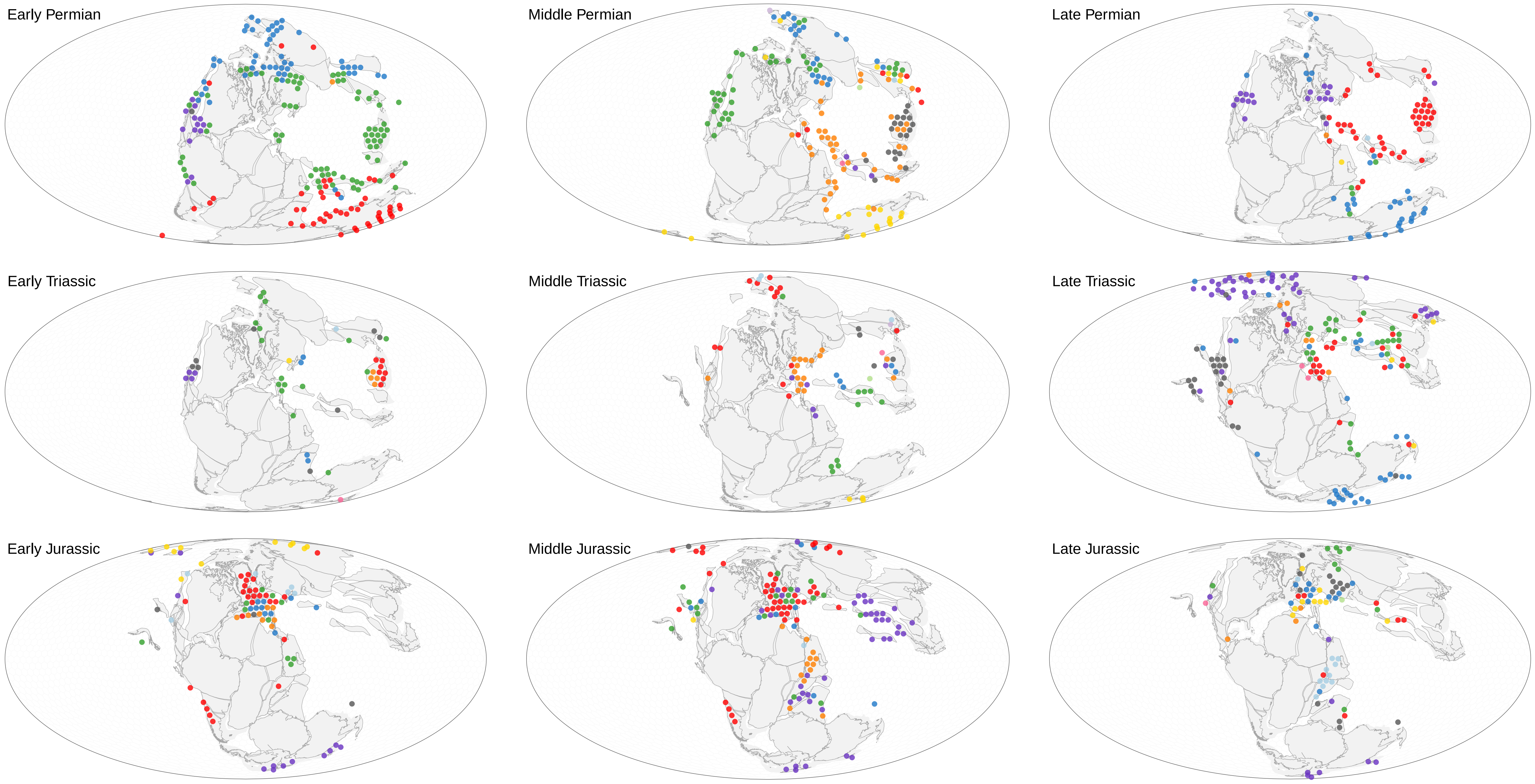

Supplementary Fig. 32. Palaeobiogeography of brachiopods indicated by network analysis.
Each color represents a group. Every interval was analysed individually.
Cells with the same color in two intervals does not mean they belong to one group.

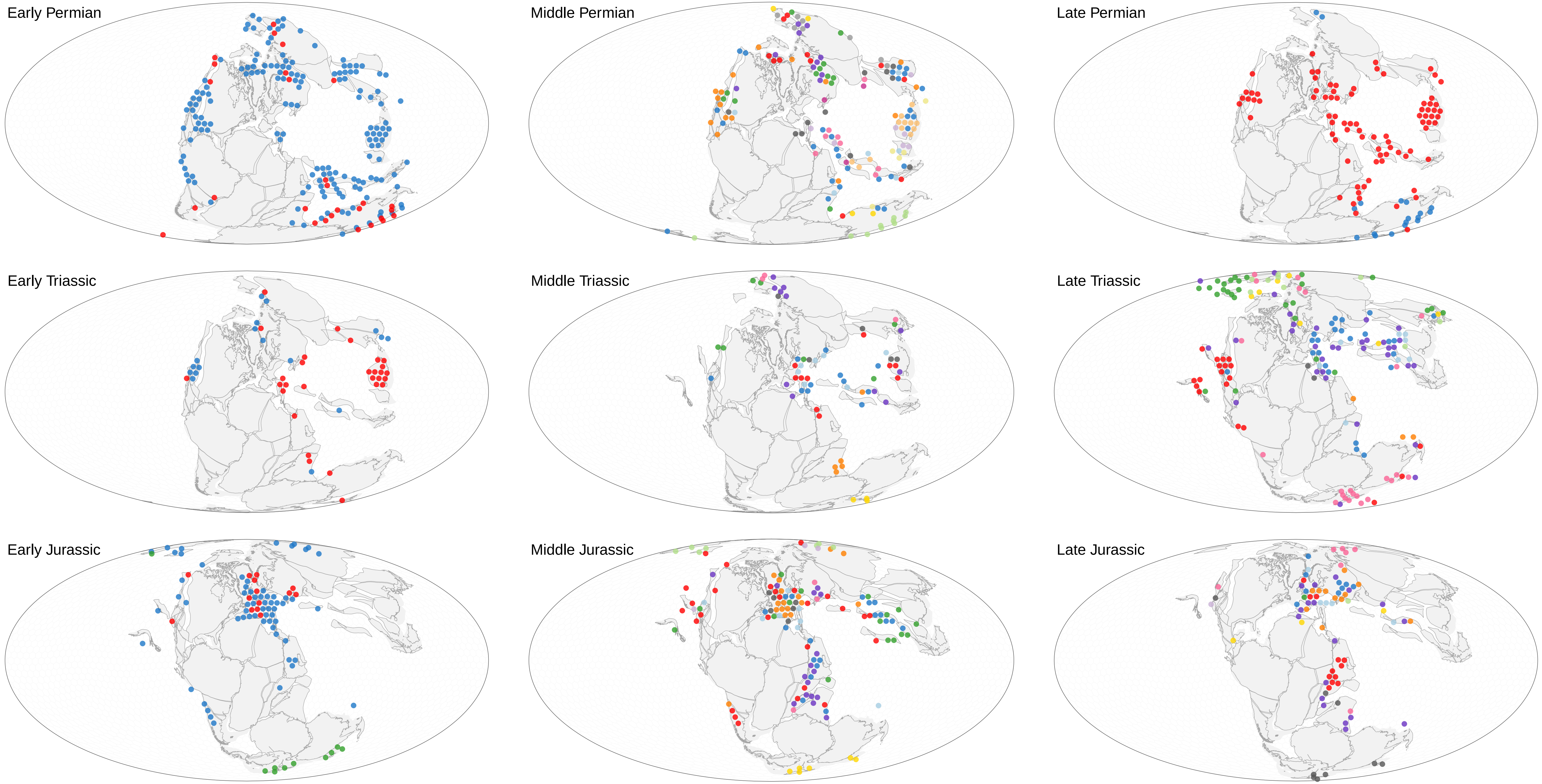

Supplementary Fig. 33. Palaeobiogeography of brachiopods indicated by PAM clustering.
Each color represents a group. Every interval was analysed individually.
Cells with the same color in two intervals does not mean they belong to one group.

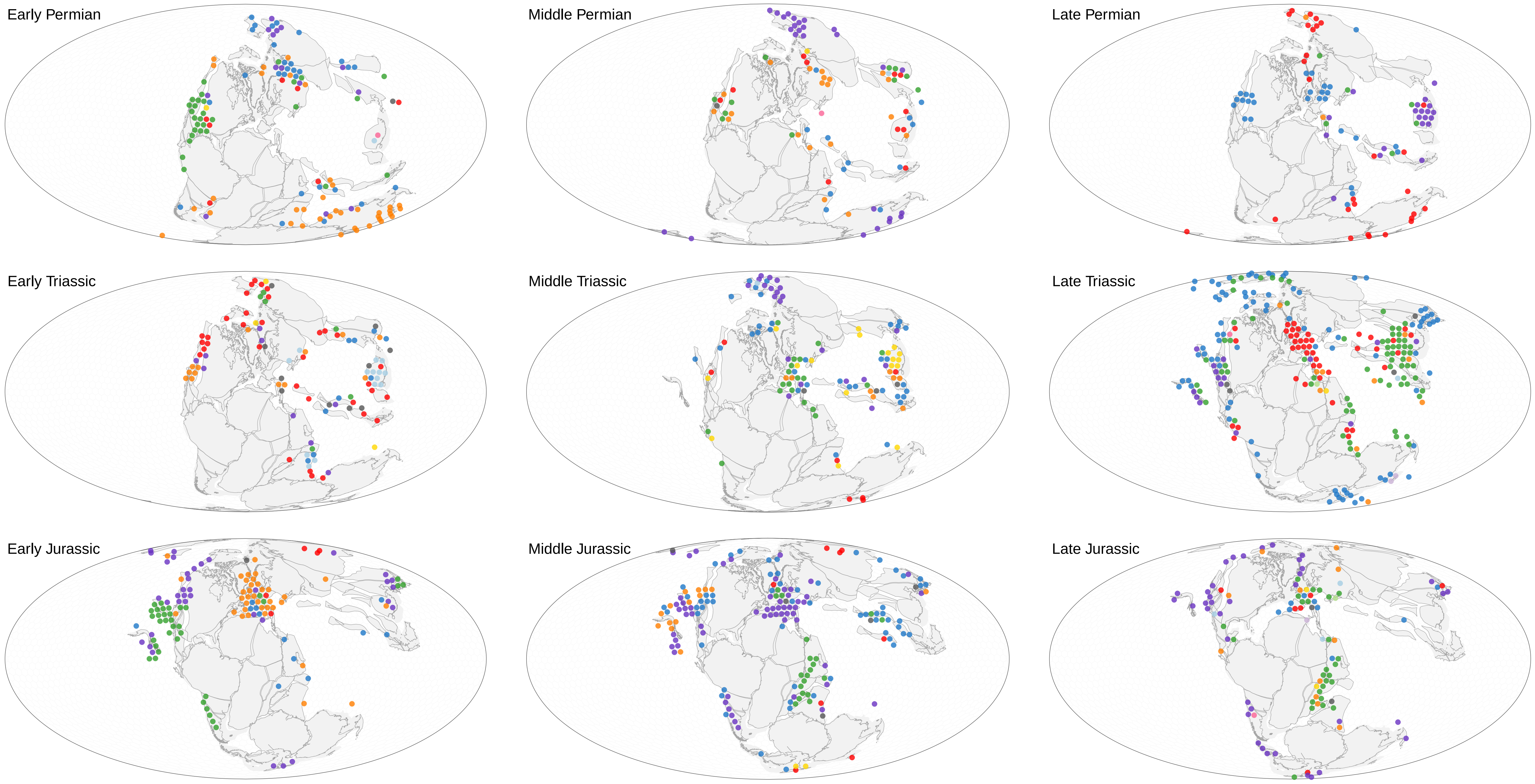

Supplementary Fig. 34. Palaeobiogeography of bivalves indicated by network analysis.
Each color represents a group. Every interval was analysed individually.
Cells with the same color in two intervals does not mean they belong to one group.

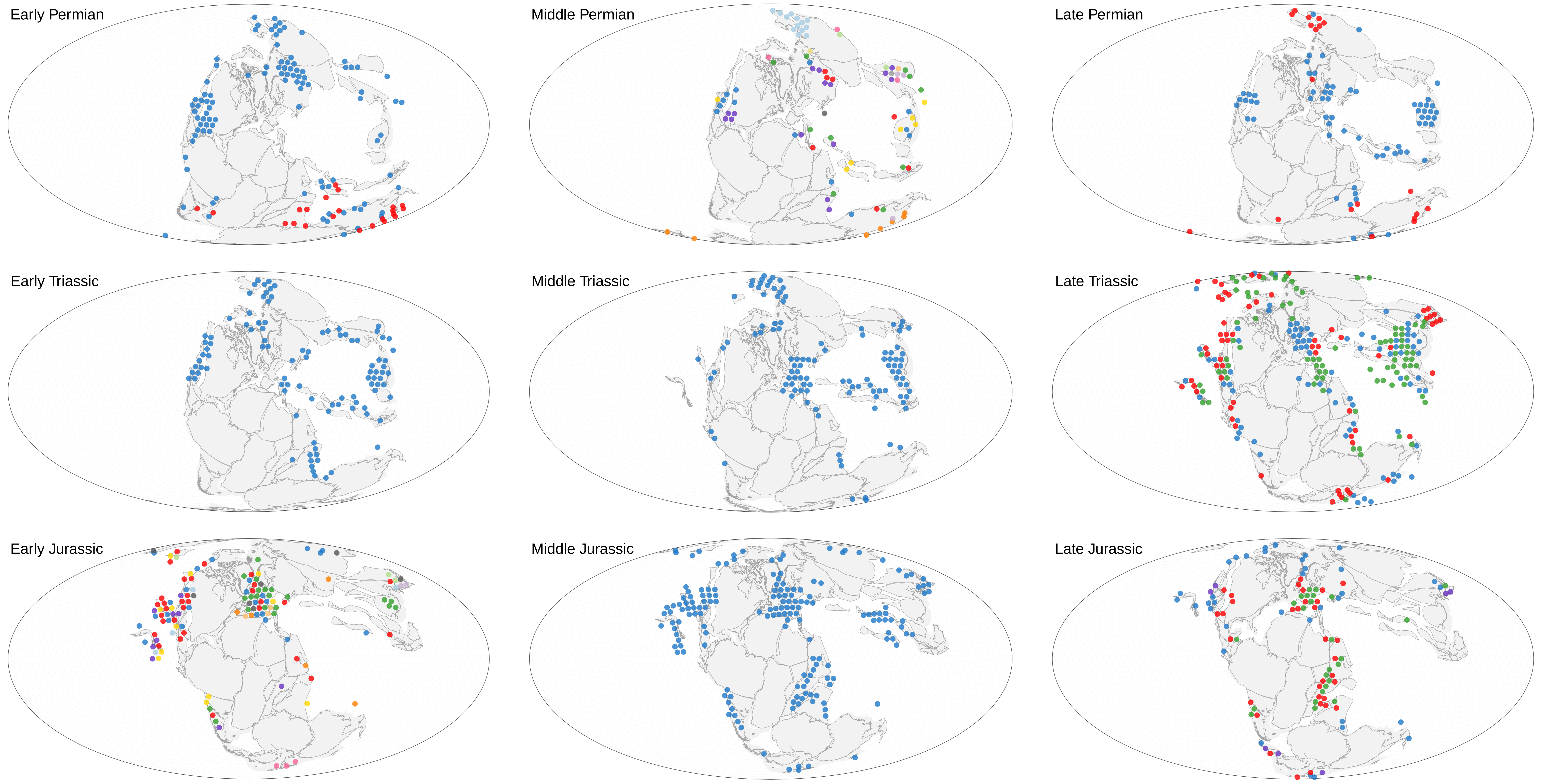

Supplementary Fig. 35. Palaeobiogeography of bivalves indicated by PAM clustering.
Each color represents a group. Every interval was analysed individually.
Cells with the same color in two intervals does not mean they belong to one group.

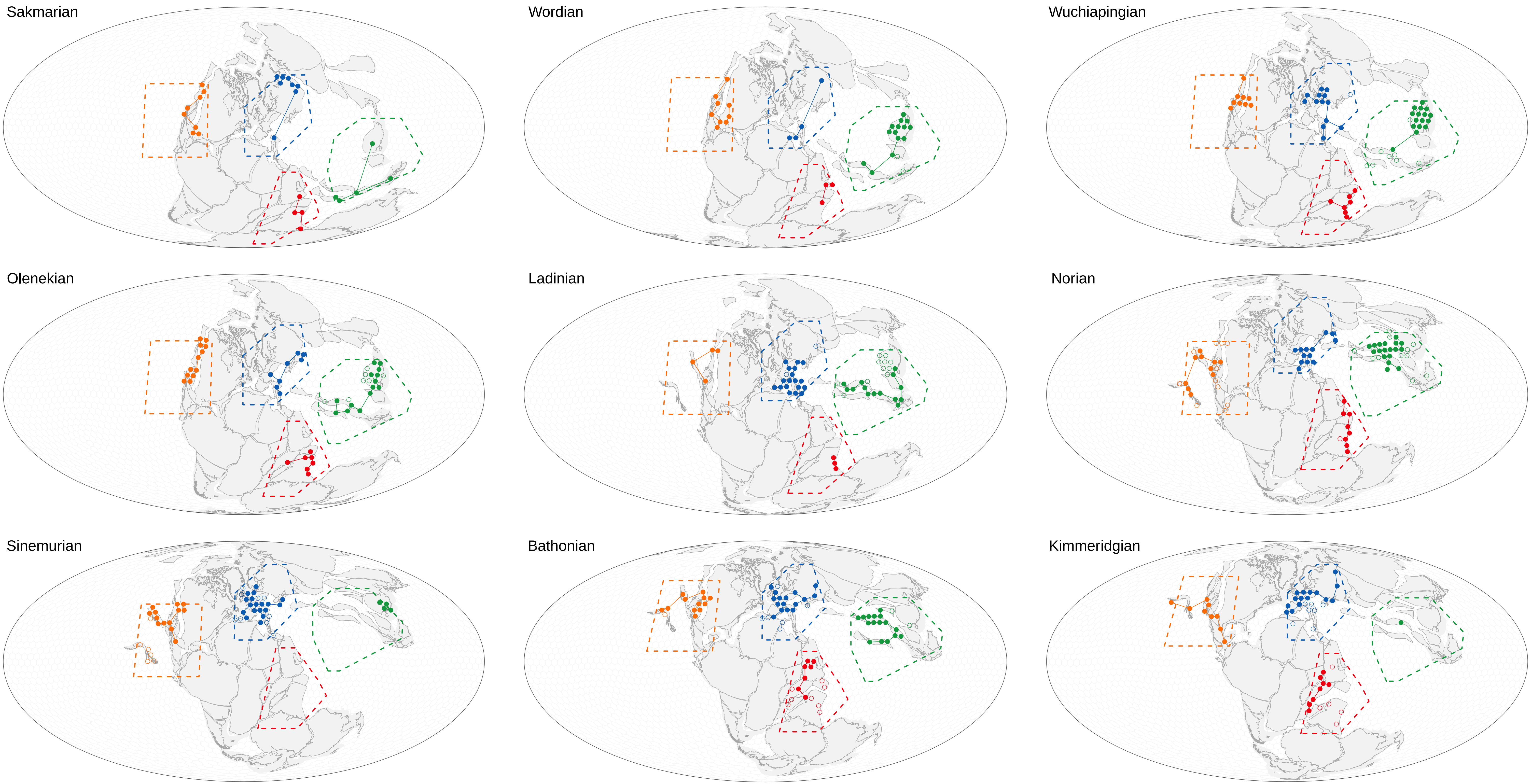

Supplementary Fig. 36. Spatial windows and their movement over time.
The blue, red, green, and orange windows represent the NW Tethys, SW Tethys, E Tethys, and N Panthalassa, respectively.
Filled dots represent cells retained after MST standardisation. Open dots represent cells discarded.

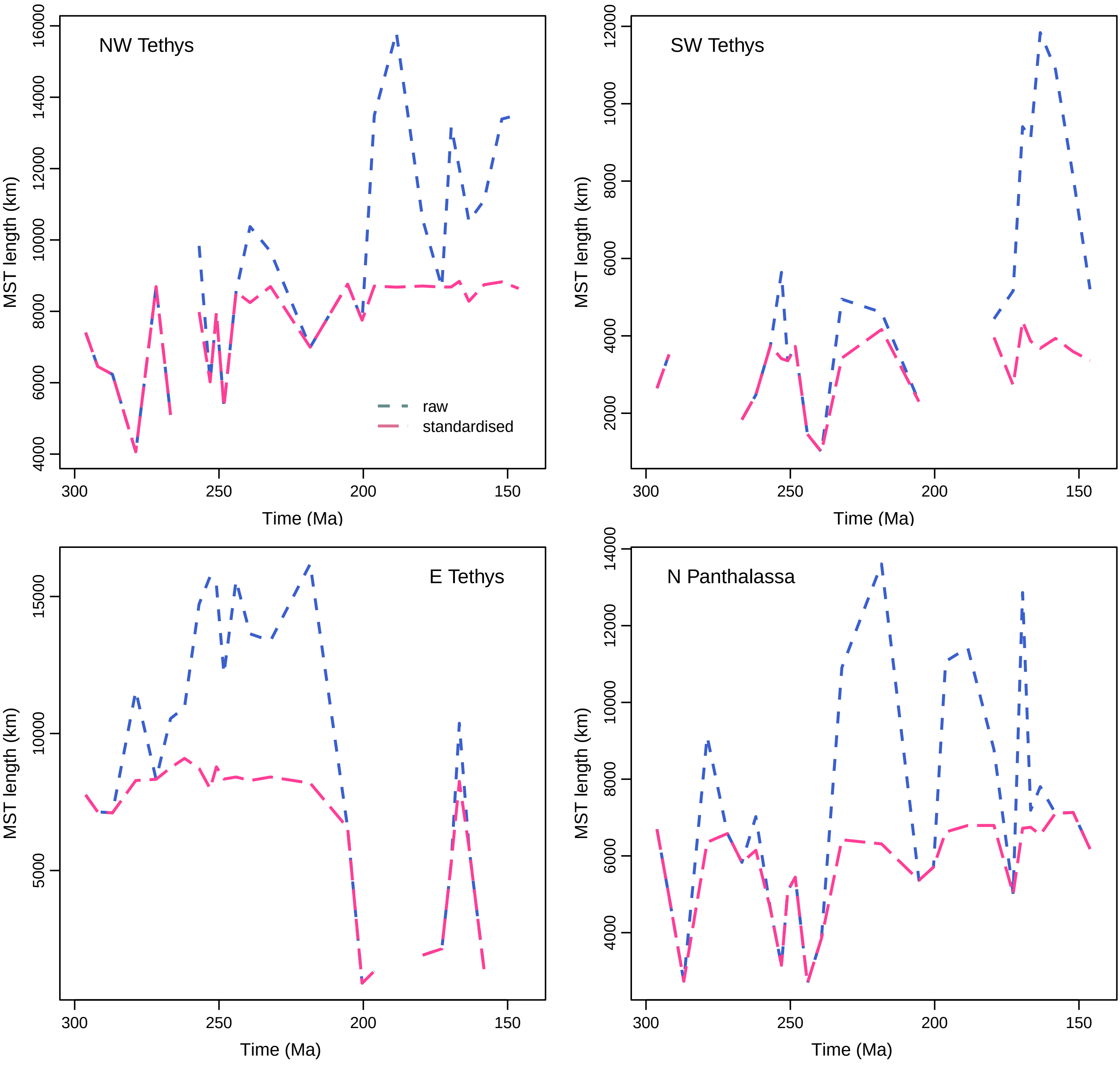

Supplementary Fig. 37. MST lengths of occurrences in the four regions before and after spatial standardisation.

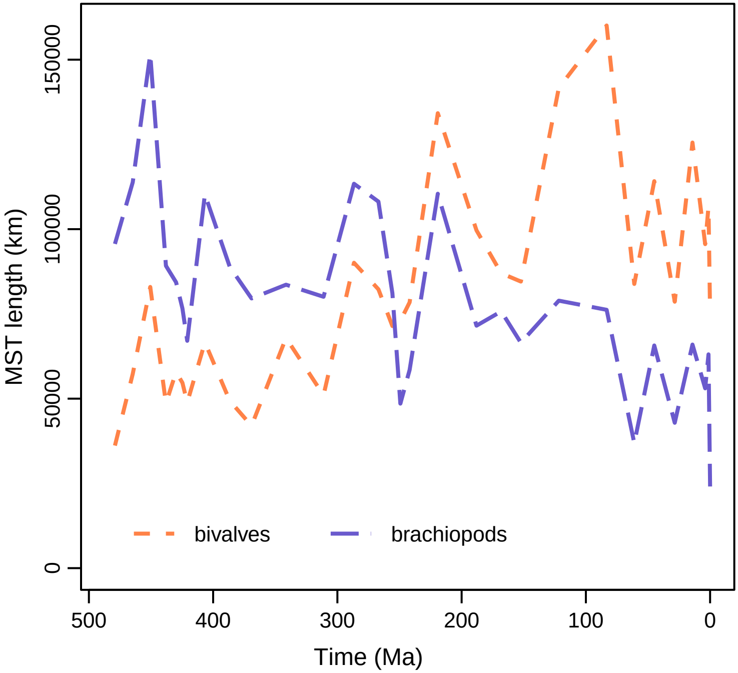

Sup. Fig. 38. MST lengths of post−Cambrian brachiopods and bivalves.

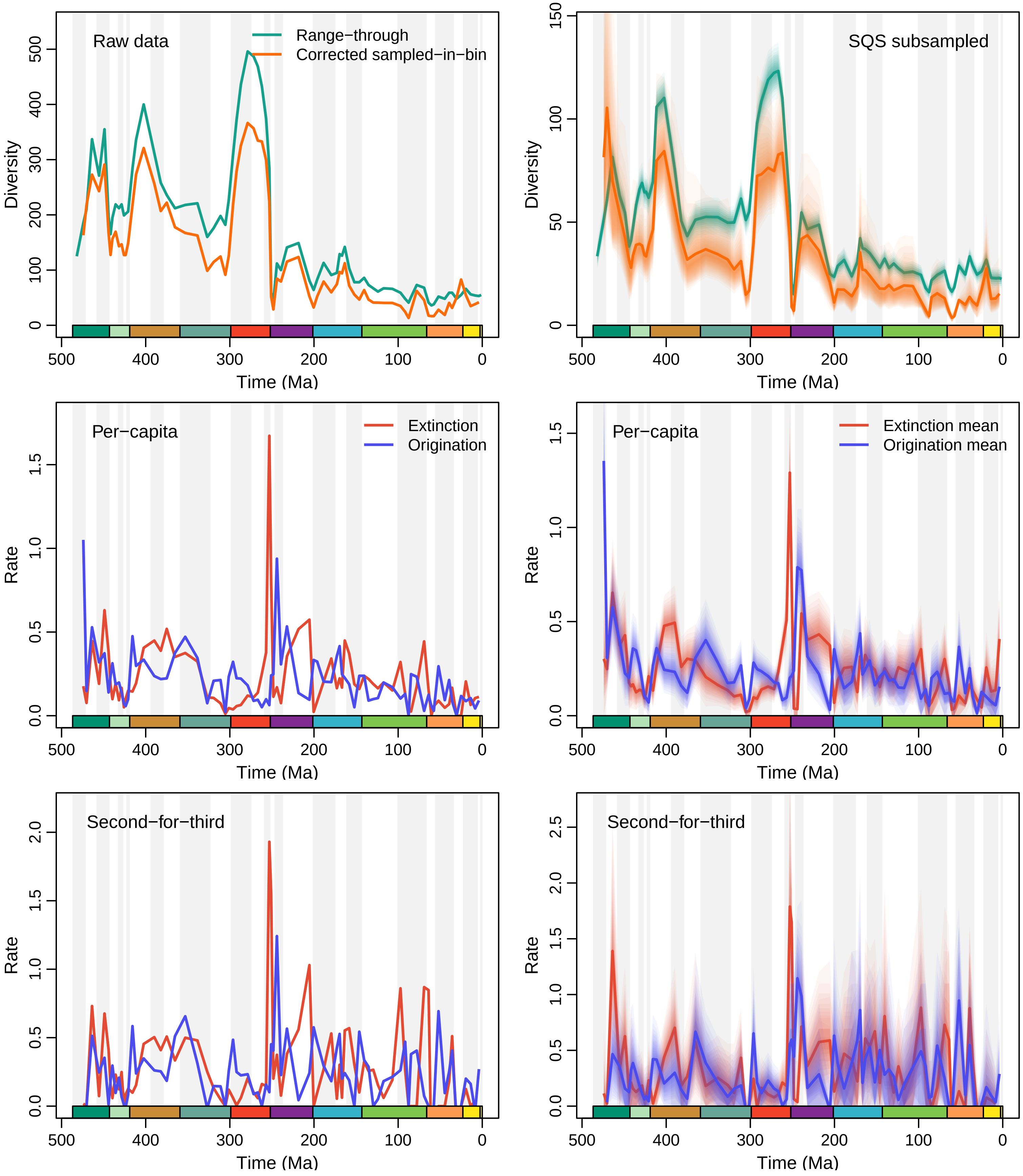

Supplementary Fig. 39. Diversities and diversification rates of post−Cambrian brachiopods generated by the divDyn package. Diversities are calculated using the range−through and corrected sampled−in−bin methods. Per−capita rate and second−for−third rate are shown. The left panel shows the results on the raw data. The right panel shows the results on SQS subsampled (q = 0.5) data.

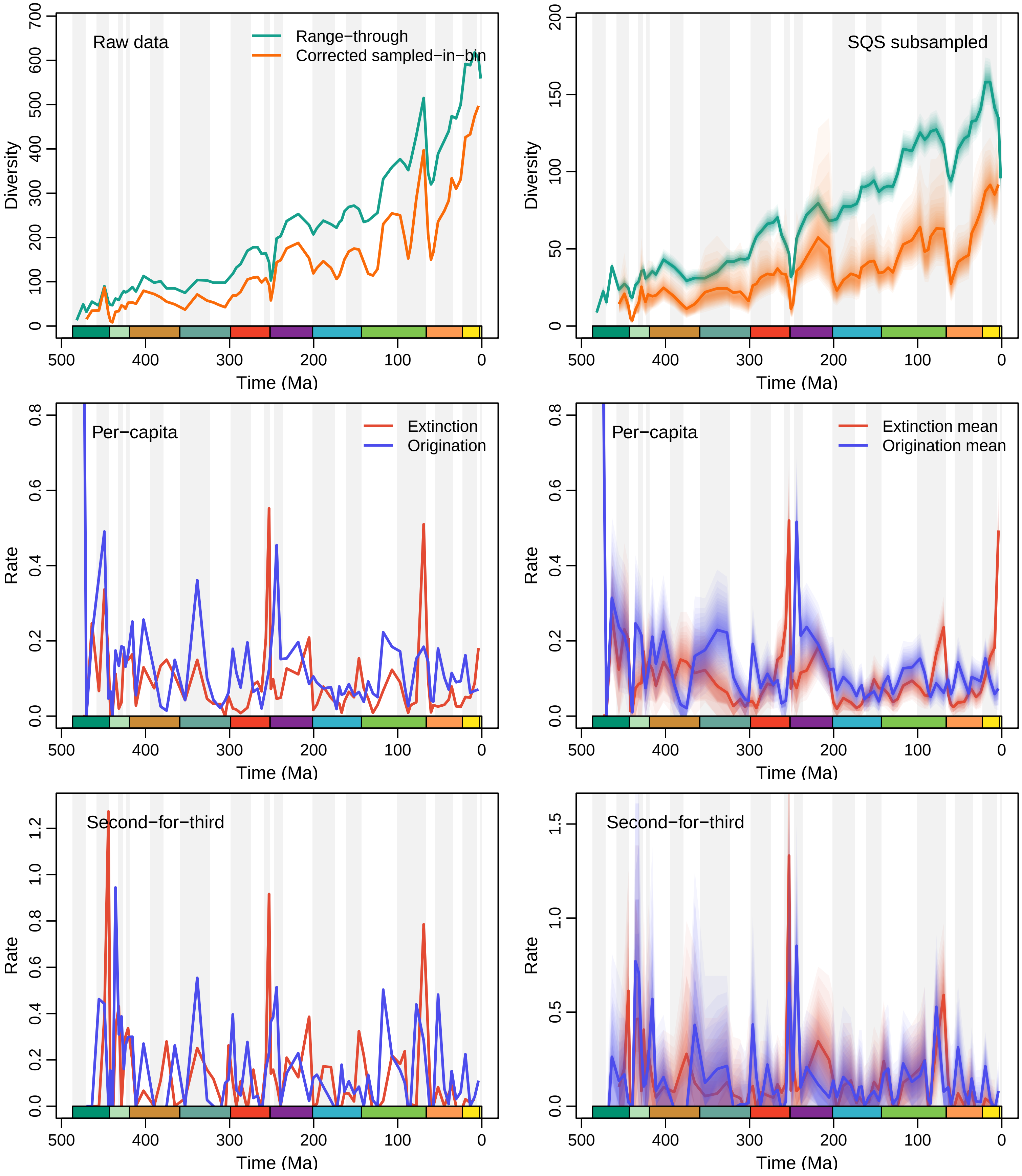

Supplementary Fig. 40. Diversities and diversification rates of post−Cambrian bivalves generated by the divDyn package. Diversities are calculated using the range−through and corrected sampled−in−bin methods. Per−capita rate and second−for−third rate are shown. The left panel shows the results on the raw data. The right panel shows the results on SQS subsampled (q = 0.5) data.

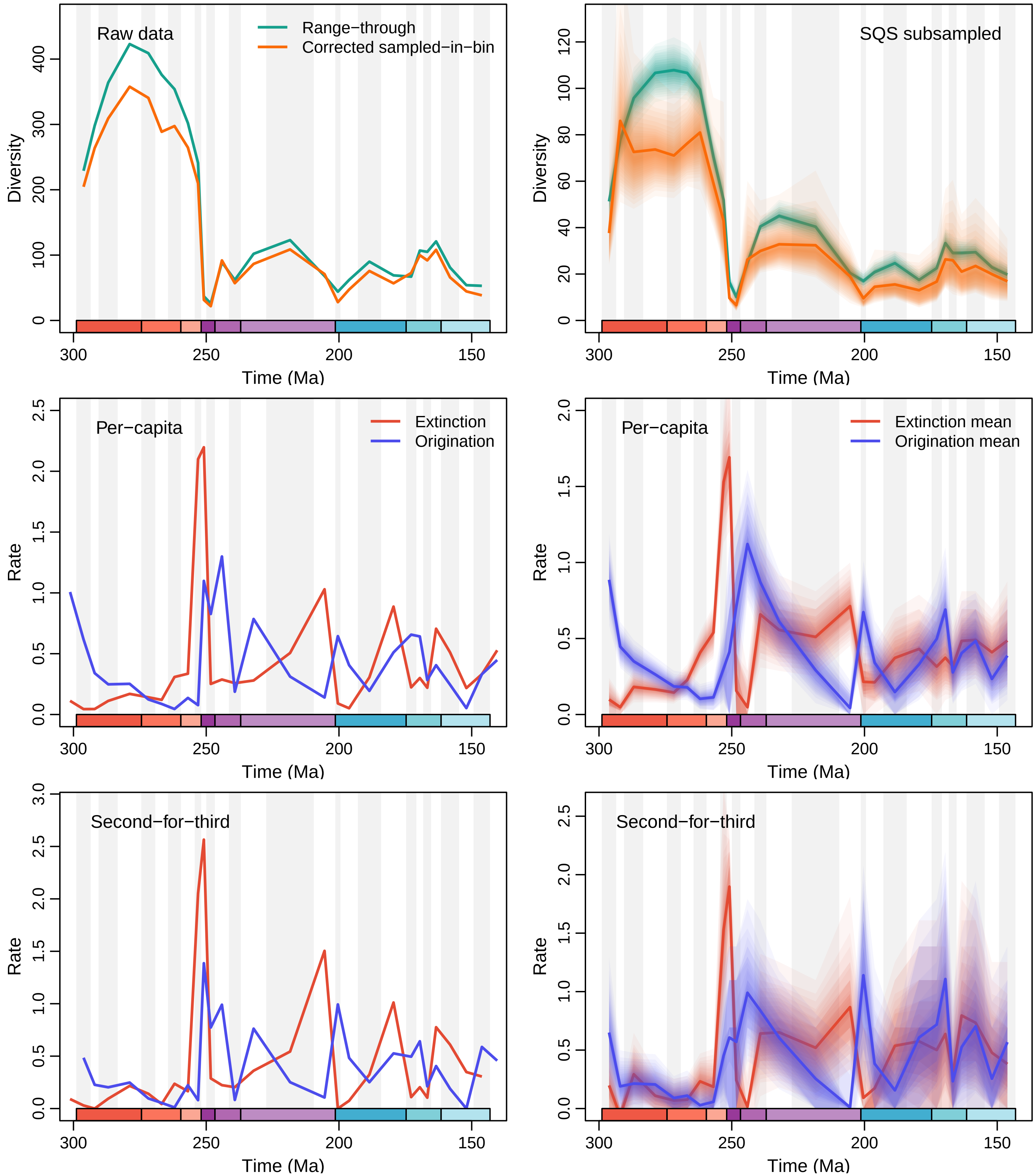

Supplementary Fig. 41. Diversities and diversification rates of Permian−Jurassic brachiopods generated by the divDyn package. Diversities are calculated using the range−through and corrected sampled−in−bin methods. Per−capita rate and second−for−third rate are shown. The left panel shows the results on the raw data. The right panel shows the results on SQS subsampled (q = 0.5) data.

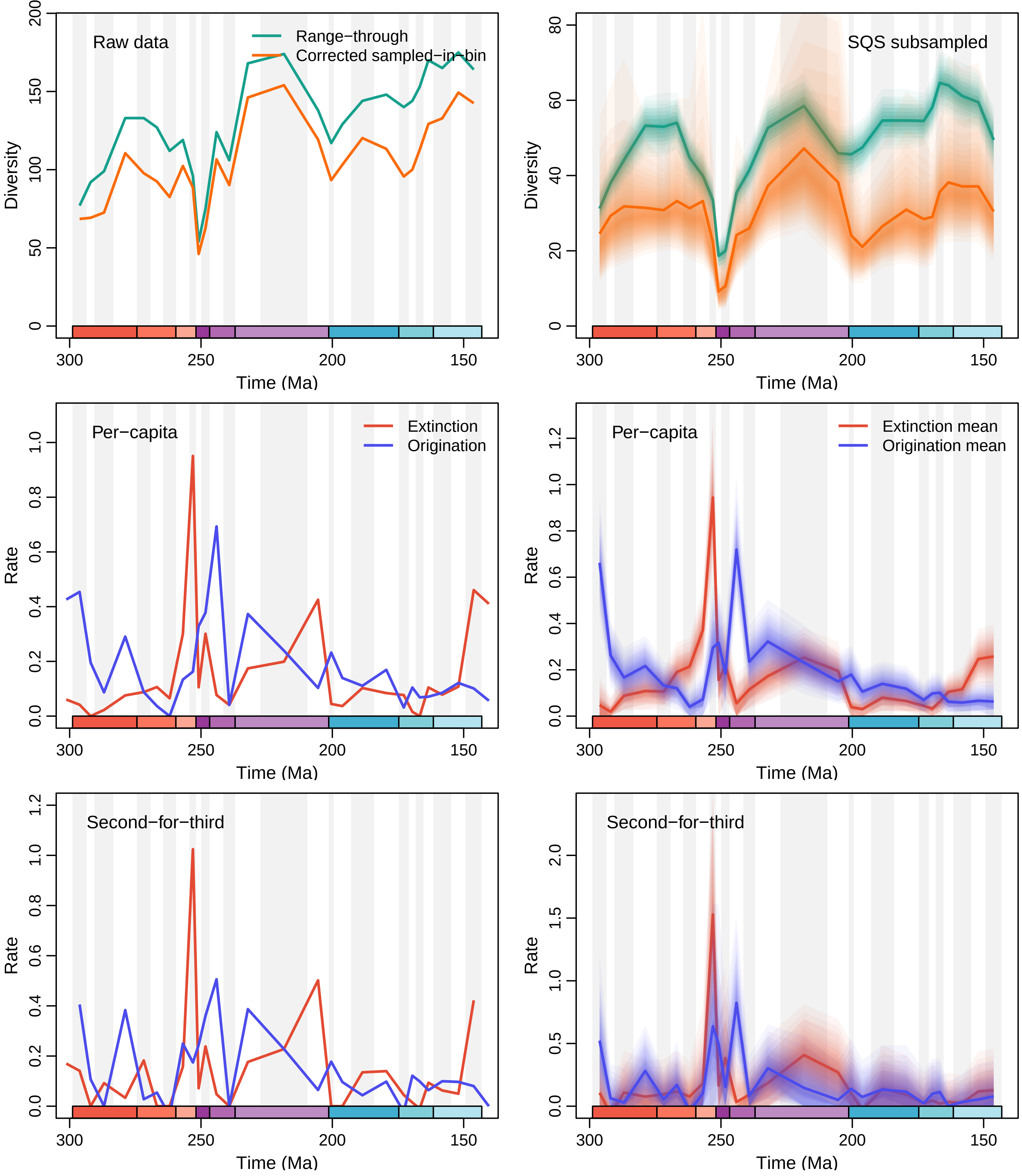

Supplementary Fig. 42. Diversities and diversification rates of Permian−Jurassic bivalves generated by the divDyn package. Diversities are calculated using the range−through and corrected sampled−in−bin methods. Per−capita rate and second−for−third rate are shown. The left panel shows the results on the raw data. The right panel shows the results on SQS subsampled (q = 0.5) data.

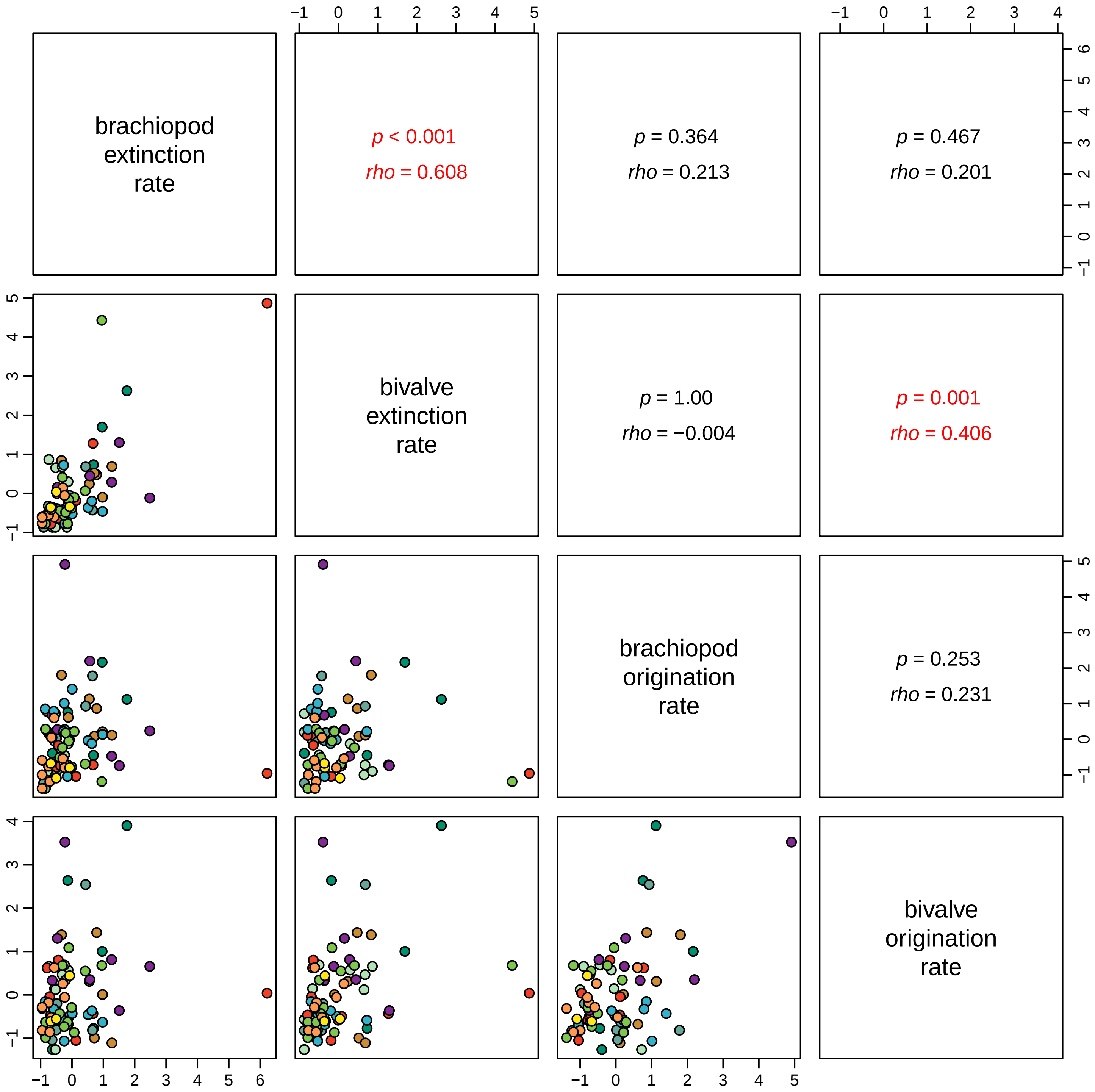

Supplementary Fig. 43. Correlations (Spearman's rho) between per−capita rates of post−Cambrian brachiopods and bivalves. The rates are based on raw data. p values are Bonferroni−adjusted.

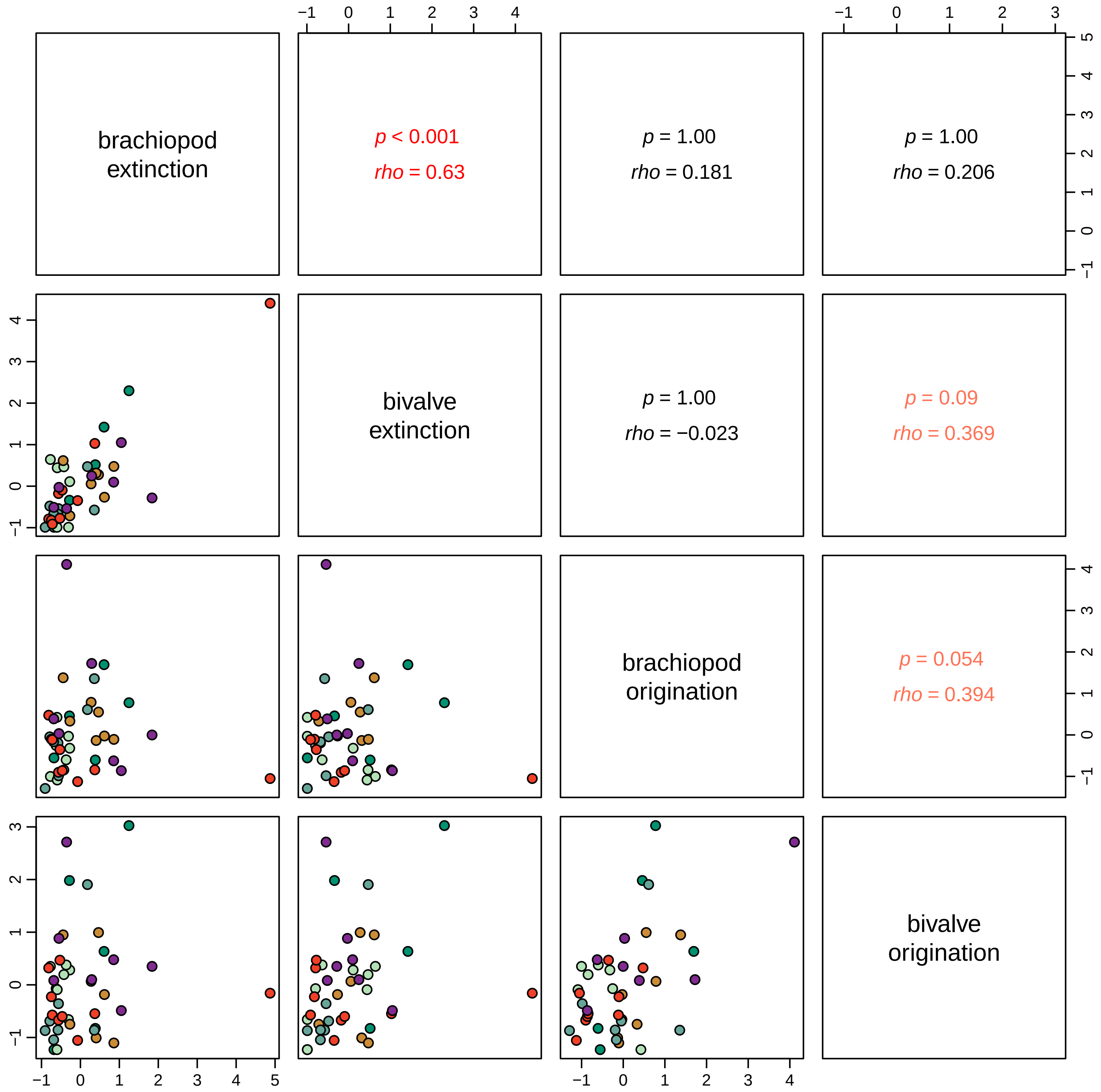

Supplementary Fig. 44. Correlations (Spearman's rho) between per−capita rates of Ordovician−Triassic brachiopods and bivalves. The rates are based on raw data. p values are Bonferroni−adjusted.

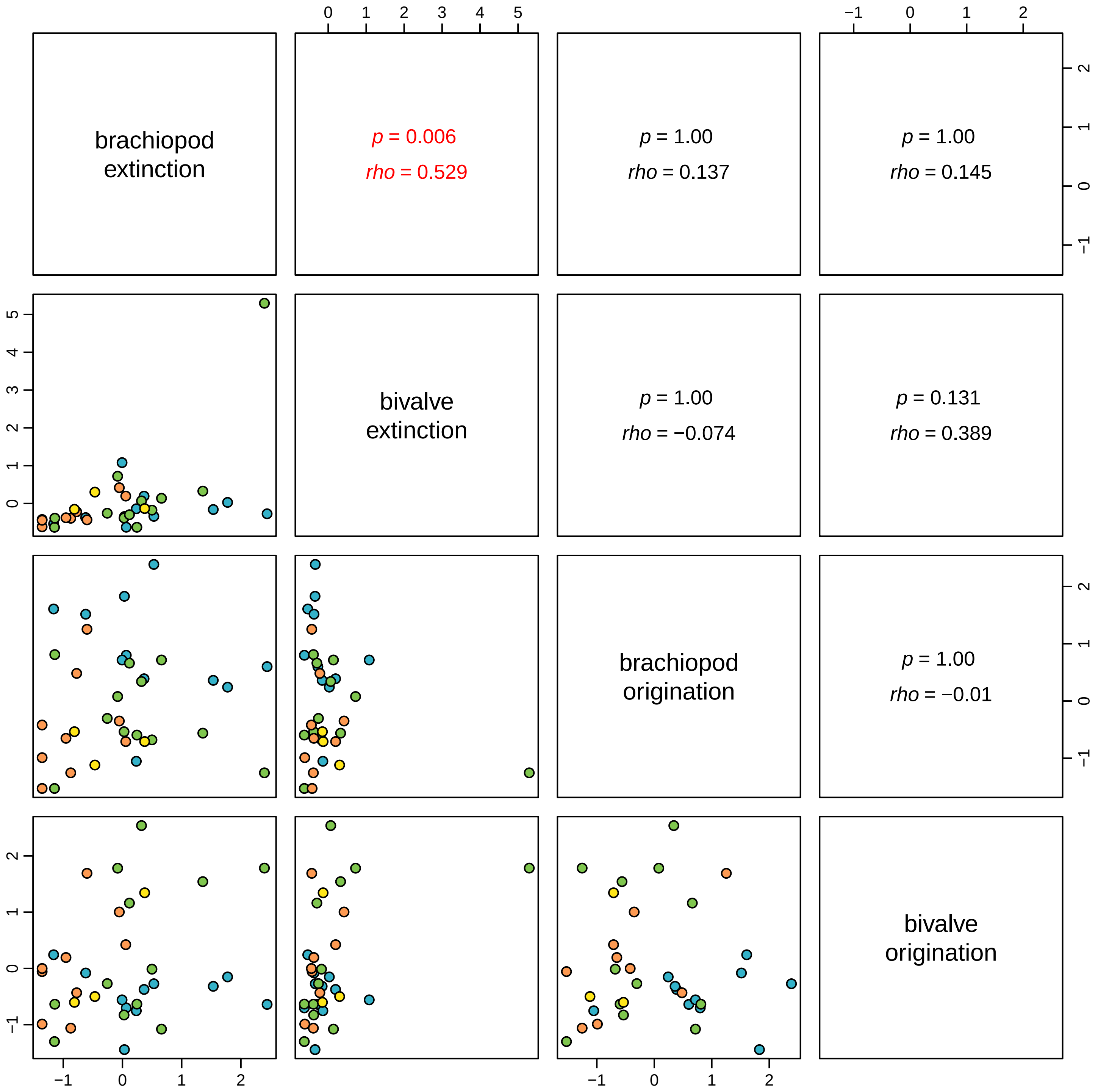

Supplementary Fig. 45. Correlations (Spearman's rho) between per−capita rates of post−Triassic brachiopods and bivalves. The rates are based on raw data. p values are Bonferroni−adjusted.

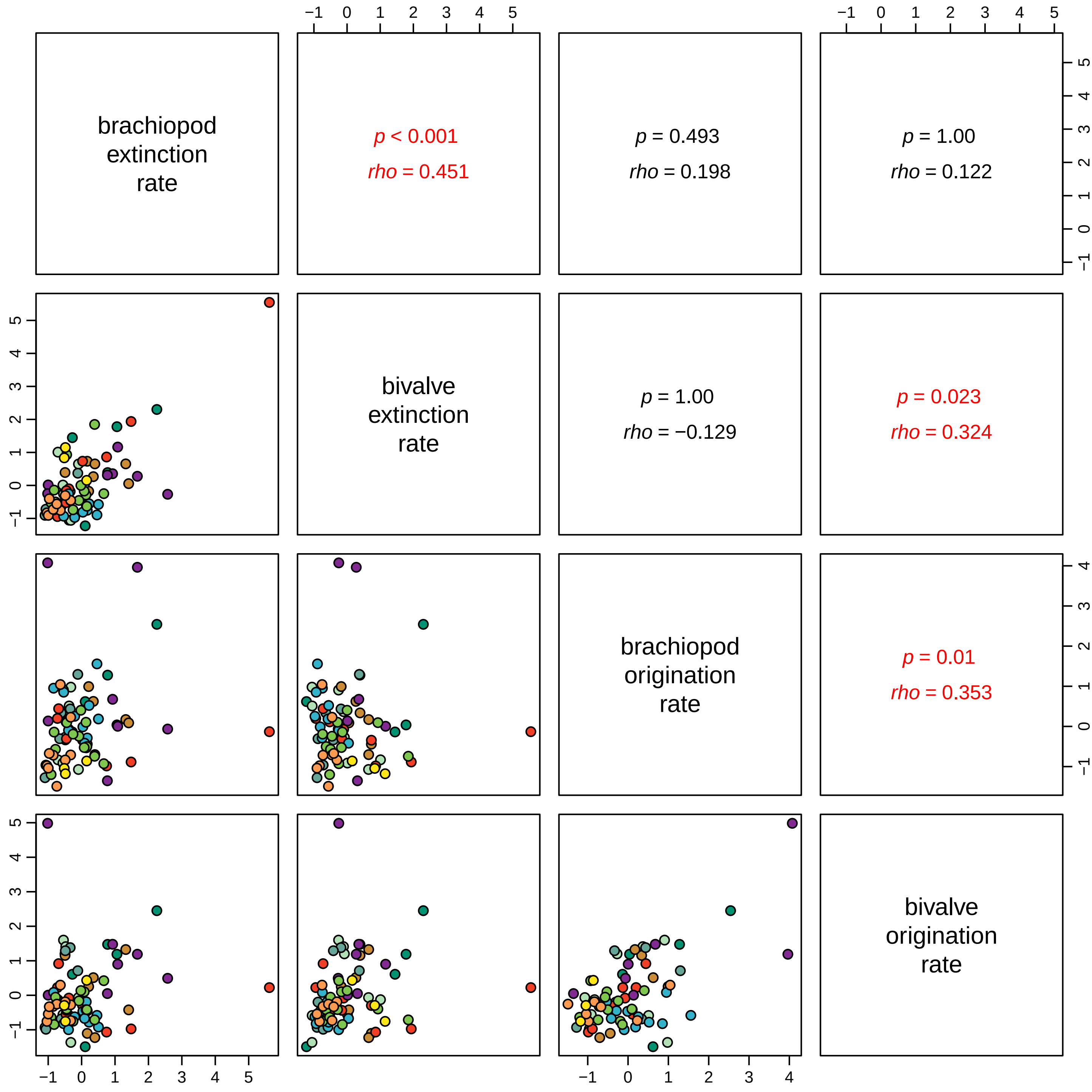

Supplementary Fig. 46. Correlations (Spearman's rho) between per−capita rates of post−Cambrian brachiopods and bivalves. The rates are based on subsampled data. p values are Bonferroni−adjusted.

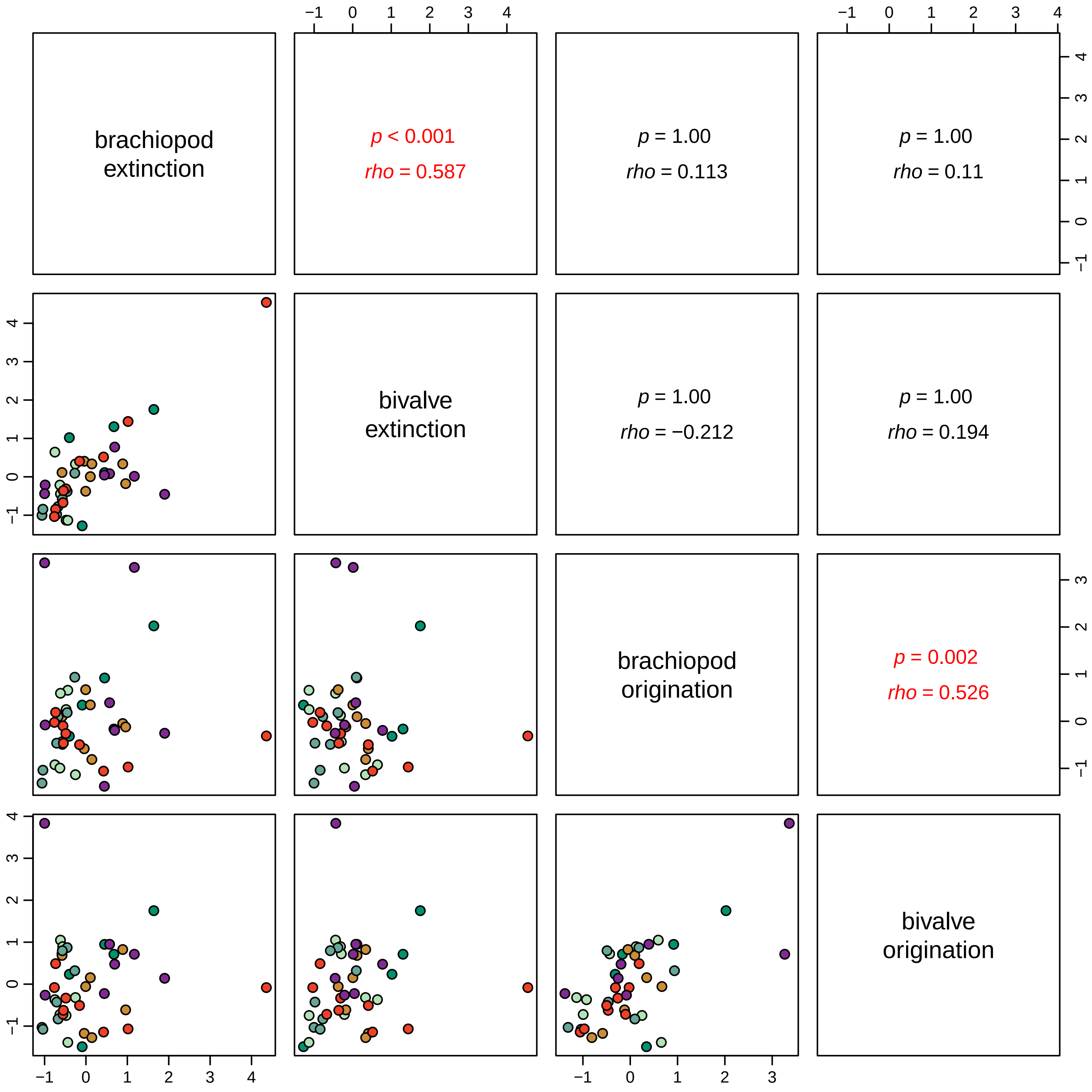

Supplementary Fig. 47. Correlations (Spearman's rho) between per−capita rates of Ordovician−Triassic brachiopods and bivalves. The rates are based on subsampled data. p values are Bonferroni−adjusted.

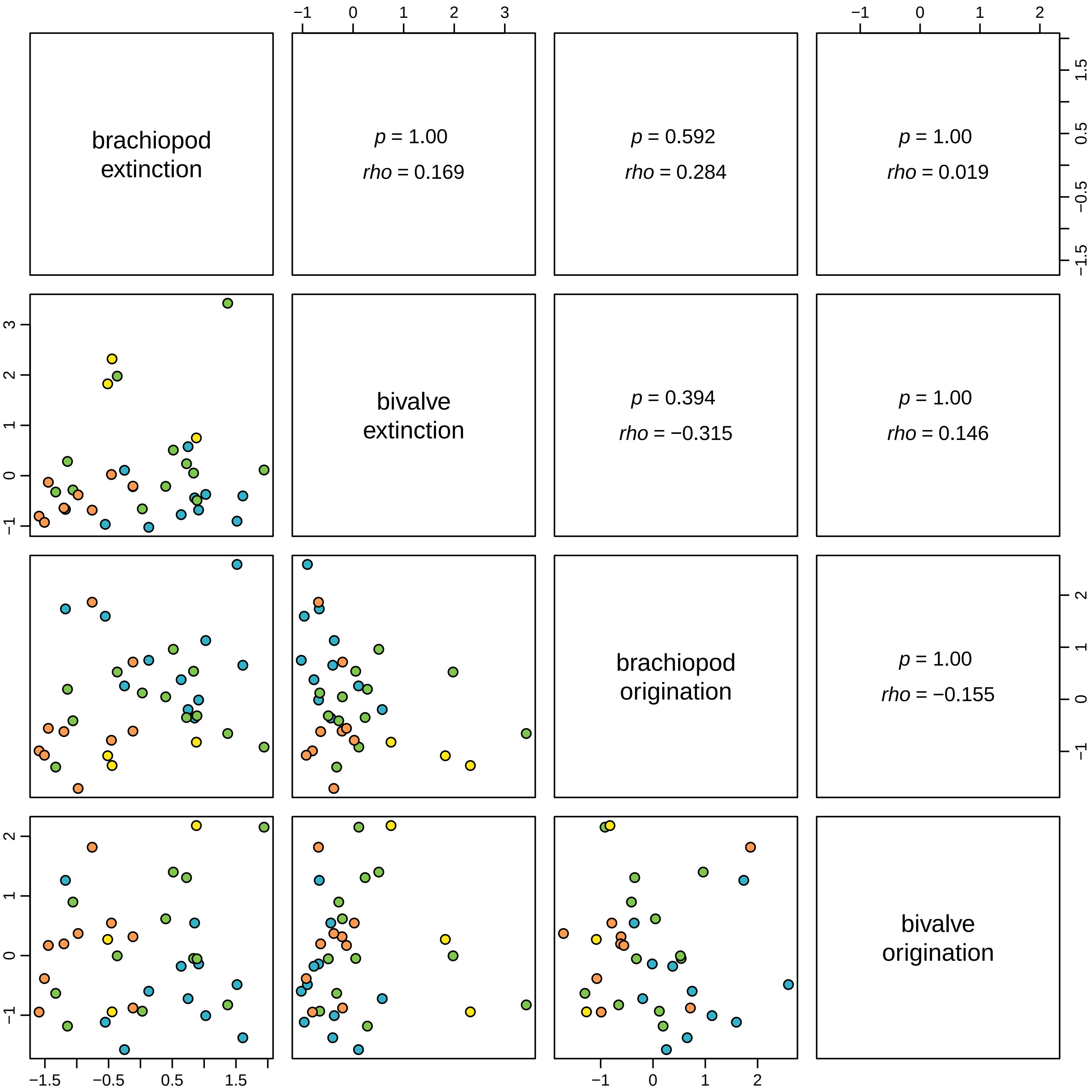

Supplementary Fig. 48. Correlations (Spearman's rho) between per−capita rates of post−Triassic brachiopods and bivalves. The rates are based on subsampled data. p values are Bonferroni−adjusted.

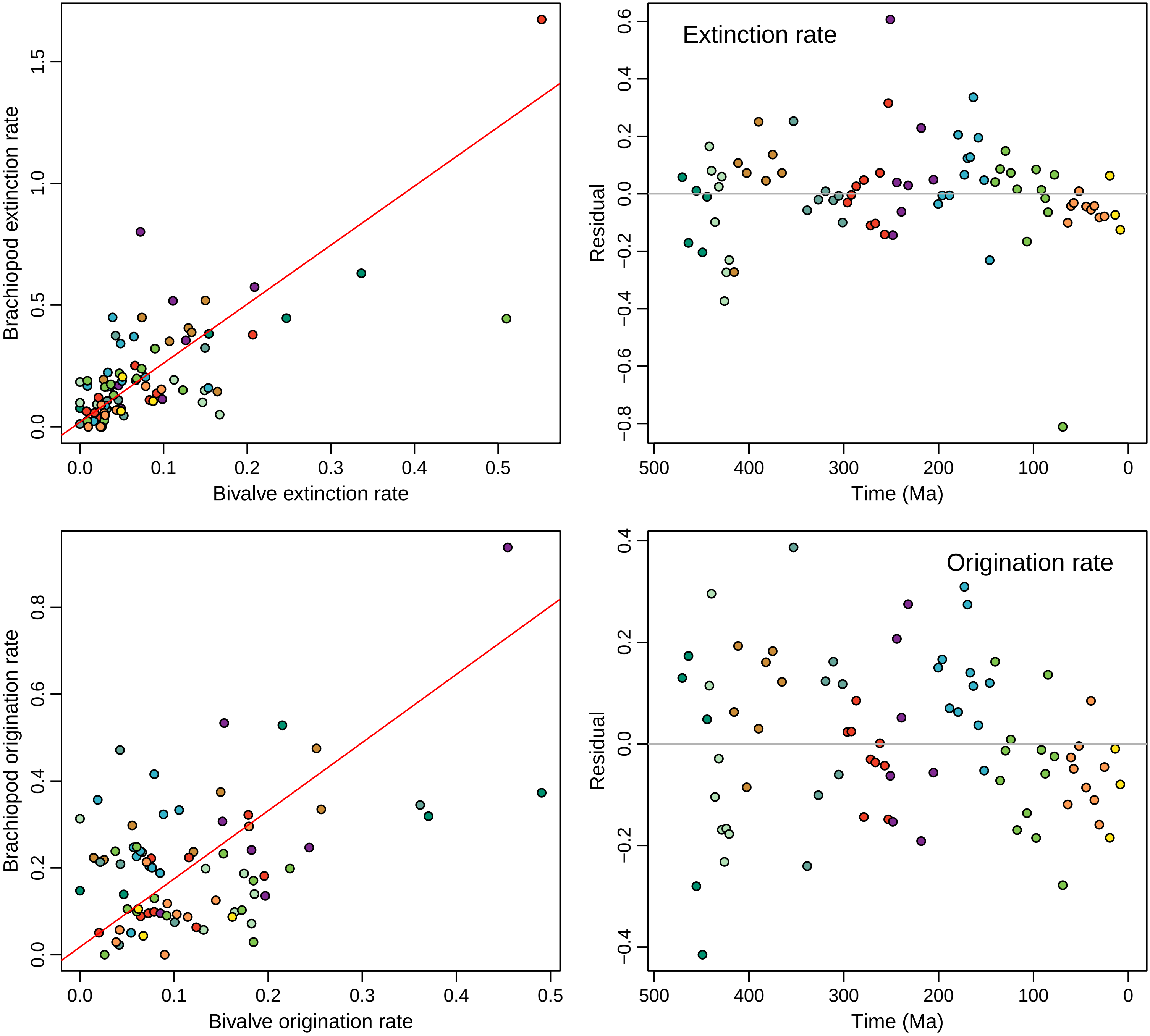

Supplementary Fig. 49. Correlations between per−capita rates of brachiopods and bivalves and the resulted residuals. The rates based on the raw data.

Supplementary Tables 1-10. Correlation parameters and shrinkage weights estimated by multivariate analyses of brachiopods and bivalves. Mean and 95% high posterior density interval (HPDI) of parameters are reported. Significant relationships (shrinkage weight $w > 0.5$) are shown in bold.

Supplementary Table 1. Permian-Jurassic Brachiopoda

	Gλ	Gλ_HPDI	wλ	Gμ	Gμ_HPDI	wμ
Bivalve diversity	**-2.42**	-2.96~-1.84	**0.82**	**-3.52**	-4.05~-3.06	**0.89**
Brachiopod diversity	0.22	-0.19~0.82	0.29	-0.33	-0.97~0.24	0.34
Predator diversity	**2.44**	1.63~3.24	**0.81**	**1.55**	0.63~2.54	**0.69**
Proportion of carbonate occs.	0.36	-0.02~0.72	0.36	-0.18	-0.56~0.14	0.28
Continental fragmentation	-0.02	-0.7~0.71	0.27	-0.49	-1.29~0.13	0.39
Sea level	**-1.04**	-1.66~-0.48	**0.59**	-0.12	-0.57~0.28	0.24
Carbon isotope	**1.17**	0.47~1.9	**0.63**	-0.55	-1.1~0.01	0.44
Strontium isotope	0.67	0.33~1.03	0.49	**-1.92**	-2.26~-1.57	**0.76**
Sulfur isotope	**1.63**	0.96~2.32	**0.7**	0.12	-0.36~0.79	0.28
Sea temperature	-0.24	-1~0.44	0.33	**2.05**	1.37~2.7	**0.76**

Supplementary Table 2. Asselian-Wordian Brachiopoda

	Gλ	Gλ_HPDI	wλ	Gμ	Gμ_HPDI	wμ
Bivalve diversity	-0.91	-3.22~1.02	0.46	0.75	-0.87~5.28	0.34
Brachiopod diversity	0.27	-1.29~2.87	0.32	-0.81	-5.32~0.92	0.33
Predator diversity	**-1.82**	-4.02~0.11	**0.61**	-1.24	-3.72~0.36	0.5
Proportion of carbonate occs.	0.45	-0.19~1.41	0.32	-0.35	-1.18~0.43	0.3
Continental fragmentation	0.85	-0.62~3.09	0.41	0.09	-1.22~1.28	0.28
Sea level	0.07	-1.36~1.29	0.29	-0.08	-1.55~1.14	0.27
Carbon isotope	-0.1	-0.89~0.49	0.21	0.61	-0.18~1.47	0.39
Strontium isotope	0.46	-0.62~2.47	0.34	-0.77	-3.8~1.01	0.39
Sulfur isotope	0.08	-0.7~1.1	0.23	0.19	-0.71~1.46	0.25
Sea temperature	**-1.72**	-3.83~0.15	**0.61**	-1.04	-3.17~0.29	0.48

Supplementary Table 3. Capitanian-Ladinian Brachiopoda

	Gλ	Gλ_HPDI	wλ	Gμ	Gμ_HPDI	wμ
Bivalve diversity	**-2.02**	-4.22~-0.19	**0.72**	**-1.5**	-3.12~0.06	**0.64**
Brachiopod diversity	0.08	-1.72~1.72	0.38	**-1.36**	-3.74~0.38	**0.57**
Predator diversity	0.27	-1.07~2.17	0.39	**1.36**	-0.21~3.09	**0.61**
Proportion of carbonate occs.	0.17	-0.97~1.45	0.36	**-1.21**	-2.2~0.06	**0.61**
Continental fragmentation	-0.4	-1.25~0.33	0.38	0.29	-0.38~1.19	0.33
Sea level	-0.72	-1.6~0.08	0.47	-0.02	-0.61~0.54	0.28
Carbon isotope	0.89	-0.16~2.4	0.5	-0.77	-1.61~0.03	0.49
Strontium isotope	**2.26**	0.64~3.87	**0.77**	**-3.16**	-4.77~-1.56	**0.86**
Sulfur isotope	1.02	-0.49~3.61	0.5	0.34	-0.54~2.07	0.36
Sea temperature	**-1.74**	-5.41~0.49	**0.58**	0.8	-0.36~2.19	0.5

Supplementary Table 4. Carnian-Toarcian Brachiopoda

	$G\lambda$	$G\lambda$_HPDI	$w\lambda$	$G\mu$	$G\mu$_HPDI	$w\mu$
Bivalve diversity	**-2.17**	-4.02~0.04	**0.72**	-0.42	-1.78~0.76	0.36
Brachiopod diversity	-0.46	-2.53~0.72	0.39	-0.33	-1.5~0.57	0.33
Predator diversity	**-1.11**	-2.4~0.02	**0.55**	**1.22**	-0.04~2.4	**0.58**
Proportion of carbonate occs.	-0.18	-0.98~0.44	0.28	-0.42	-1.21~0.21	0.34
Continental fragmentation	0.92	-0.71~3.5	0.47	**-1.16**	-3.3~0.33	**0.53**
Sea level	-0.56	-1.96~0.33	0.38	-0.25	-1.41~0.5	0.31
Carbon isotope	**2.2**	0.74~3.93	**0.76**	**1.63**	0.16~3.01	**0.67**
Strontium isotope	**-1.24**	-2.45~0.1	**0.59**	0.02	-0.63~1.04	0.26
Sulfur isotope	-0.68	-2.36~0.31	0.42	0.57	-0.49~2.05	0.41
Sea temperature	0.76	-0.38~1.99	0.45	0.02	-0.63~0.75	0.26

Supplementary Table 5. Aalenian-Tithonian Brachiopoda

	$G\lambda$	$G\lambda$_HPDI	$w\lambda$	$G\mu$	$G\mu$_HPDI	$w\mu$
Bivalve diversity	-0.71	-2.78~0.7	0.36	-0.08	-1.12~1.01	0.24
Brachiopod diversity	**1.7**	0.47~2.96	**0.67**	0.2	-0.38~0.93	0.22
Predator diversity	0.12	-1.37~1.48	0.26	-0.02	-1.11~1.09	0.23
Proportion of carbonate occs.	-0.42	-1.41~0.36	0.3	0.15	-0.64~1.07	0.24
Continental fragmentation	**-1.27**	-3.07~0.22	**0.52**	-0.66	-2.16~0.45	0.38
Sea level	0.02	-1~1.49	0.25	-0.3	-1.13~0.36	0.27
Carbon isotope	-0.22	-0.82~0.24	0.2	0.18	-0.44~0.81	0.21
Strontium isotope	**1.08**	-0.1~1.95	**0.52**	-0.59	-1.33~0.06	0.36
Sulfur isotope	0.3	-0.64~2.03	0.28	-0.38	-1.68~0.56	0.29
Sea temperature	-0.45	-1.8~0.38	0.31	-0.83	-1.91~0.13	0.45

Supplementary Table 6. Permian-Jurassic Bivalvia

	$G\lambda$	$G\lambda$_HPDI	$w\lambda$	$G\mu$	$G\mu$_HPDI	$w\mu$
Bivalve diversity	**-2.65**	-3.34~-2.06	**0.83**	**-2.72**	-3.49~-1.95	**0.84**
Brachiopod diversity	0.02	-0.78~0.83	0.29	**-0.93**	-2.14~0.12	**0.52**
Predator diversity	**1.12**	-0.04~2	**0.59**	-0.75	-2.17~0.57	0.46
Proportion of carbonate occs.	0.53	-0.14~1.2	0.42	-0.58	-1.32~0.11	0.42
Continental fragmentation	0.15	-0.46~1.06	0.3	**1.89**	0.85~2.95	**0.74**
Sea level	-0.06	-0.61~0.54	0.25	-0.26	-1.03~0.36	0.31
Carbon isotope	0.65	-0.28~1.91	0.45	-0.6	-1.55~0.21	0.43
Strontium isotope	0.48	0~0.89	0.39	-0.5	-1.05~0.07	0.41
Sulfur isotope	0.02	-0.69~0.66	0.27	**-2.98**	-3.86~-1.93	**0.85**
Sea temperature	**0.86**	-0.06~1.66	**0.51**	**2.92**	1.71~4.14	**0.85**

Supplementary Table 7. Asselian-Wordian Bivalvia

	Gλ	Gλ_HPDI	$w\lambda$	Gμ	Gμ_HPDI	$w\mu$
Bivalve diversity	-0.4	-2.5~0.88	0.29	-0.19	-2.31~1.77	0.28
Brachiopod diversity	-0.89	-3.43~0.49	0.4	-0.96	-3.67~0.93	0.39
Predator diversity	-0.08	-1.29~1.31	0.24	-0.84	-3.36~0.54	0.39
Proportion of carbonate occs.	0.05	-0.48~0.7	0.17	-0.04	-0.91~1	0.21
Continental fragmentation	-0.08	-1.4~0.74	0.2	-0.64	-2.84~0.55	0.33
Sea level	0.3	-0.73~2.05	0.25	-0.07	-1.86~1.24	0.23
Carbon isotope	-0.3	-1.64~0.45	0.25	-0.01	-0.98~1.07	0.19
Strontium isotope	0.19	-1.07~1.96	0.25	-0.89	-3.97~0.86	0.39
Sulfur isotope	0.03	-1.12~1.25	0.21	-0.5	-2.18~0.75	0.32
Sea temperature	-0.79	-2.69~0.44	0.39	-0.22	-2.13~0.96	0.24

Supplementary Table 8. Capitanian-Ladinian Bivalvia

	Gλ	Gλ_HPDI	$w\lambda$	Gμ	Gμ_HPDI	$w\mu$
Bivalve diversity	**-0.95**	-2.69~0.51	**0.51**	**-3.5**	-7.09~-0.45	**0.83**
Brachiopod diversity	**-1.49**	-4.73~0.85	**0.6**	**1.59**	-1.06~6.39	**0.53**
Predator diversity	0.4	-0.5~2.21	0.38	**1.2**	-0.77~4.4	**0.52**
Proportion of carbonate occs.	0.17	-0.79~1.26	0.35	-0.32	-1.43~0.44	0.36
Continental fragmentation	0.52	-0.56~1.7	0.41	-0.76	-2.62~0.51	0.45
Sea level	**-1.96**	-3.2~-0.62	**0.74**	-0.71	-1.63~0.22	0.46
Carbon isotope	0.68	-0.82~3.21	0.44	-0.78	-2.39~0.38	0.47
Strontium isotope	0.52	-0.57~2.12	0.4	0.38	-0.84~1.97	0.39
Sulfur isotope	-0.02	-1.25~1.41	0.37	**-2.72**	-4.9~-0.23	**0.8**
Sea temperature	0.69	-0.73~3.6	0.46	**1.57**	-0.53~4.45	**0.62**

Supplementary Table 9. Carnian-Toarcian Bivalvia

	Gλ	Gλ_HPDI	$w\lambda$	Gμ	Gμ_HPDI	$w\mu$
Bivalve diversity	-0.18	-2.08~1.2	0.37	-0.53	-2.42~0.78	0.44
Brachiopod diversity	**-1.58**	-2.9~0	**0.67**	-0.63	-2.11~0.39	0.43
Predator diversity	**-2.2**	-3.31~-1.11	**0.78**	-0.24	-1.5~0.75	0.32
Proportion of carbonate occs.	0.05	-0.55~0.61	0.27	0.07	-0.63~0.94	0.28
Continental fragmentation	-0.77	-2.72~0.7	0.46	**-2.08**	-4.37~0.14	**0.71**
Sea level	**-1.34**	-2.79~0.15	**0.6**	0.17	-0.75~1.25	0.31
Carbon isotope	**1.73**	0.18~3.31	**0.69**	0.4	-0.45~1.64	0.37
Strontium isotope	**-2.75**	-4.25~-1.45	**0.82**	-0.19	-1.24~0.83	0.32
Sulfur isotope	**-1.58**	-2.94~0.1	**0.67**	0.13	-0.82~1.28	0.32
Sea temperature	**1.5**	-0.08~2.74	**0.66**	0.04	-0.6~0.77	0.26

Supplementary Table 10. Aalenian-Tithonian Bivalvia

	Gλ	Gλ_HPDI	$w\lambda$	Gμ	Gμ_HPDI	$w\mu$
Bivalve diversity	-0.18	-1.31~0.68	0.18	-0.33	-1.98~0.6	0.25
Brachiopod diversity	0.28	-0.37~1.21	0.19	-0.01	-0.64~0.71	0.14
Predator diversity	0.14	-0.59~1.18	0.17	-0.77	-2.54~0.26	0.38
Proportion of carbonate occs.	-0.03	-0.78~0.57	0.14	-0.23	-1.37~0.61	0.19
Continental fragmentation	-0.93	-3~0.28	0.39	0	-0.85~0.85	0.15
Sea level	0.12	-0.51~0.98	0.16	-0.11	-1.07~0.39	0.15
Carbon isotope	-0.02	-0.62~0.77	0.14	0.07	-0.85~0.78	0.16
Strontium isotope	0.01	-0.46~0.56	0.13	-0.11	-0.75~0.36	0.15
Sulfur isotope	-0.58	-2.68~0.27	0.29	-0.09	-1.11~0.8	0.18
Sea temperature	0	-0.88~0.81	0.15	**-2.06**	-3.35~-0.67	**0.7**

Supplementary Tables 11-25. Correlation parameters and shrinkage weights estimated by multivariate analyses of epifaunal brachiopods, epifaunal bivalves and infaunal bivalves. Mean and 95% high posterior density intervals (HPDI) of parameters are reported. Significant relationships (shrinkage weight $w > 0.5$) are shown in bold. Note: Some replications of the Asselian-Wordian epifaunal brachiopod analysis did not reach convergence after 100 million generations (ESSs < 200). Interpretation of estimated parameter values requires caution.

Supplementary Table 11. Permian-Jurassic epifaunal brachiopods

	Gλ	Gλ_HPDI	$w\lambda$	Gμ	Gμ_HPDI	$w\mu$
Infaunal bivalve div.	**-3.84**	-5.27~-2.43	**0.9**	**-4.42**	-6.14~-2.75	**0.92**
Epifaunal bivalve div.	0.01	-0.76~0.64	0.29	-0.47	-1.32~0.24	0.4
Epifaunal brachiopod div.	-0.11	-0.67~0.43	0.28	**-1.02**	-1.77~-0.29	**0.59**
Predator diversity	**3.96**	2.59~5.09	**0.91**	**2.54**	1~4.02	**0.82**
Proportion of carbonate occs.	0.16	-0.2~0.64	0.27	-0.24	-0.7~0.13	0.31
Continental fragmentation	-0.37	-1.3~0.46	0.38	-0.57	-1.39~0.18	0.43
Sea level	**-0.89**	-1.62~-0.16	**0.55**	-0.11	-0.66~0.28	0.25
Carbon isotope	**1.36**	0.49~2.39	**0.66**	-0.62	-1.13~0.03	0.48
Strontium isotope	**0.72**	0.34~1.11	**0.52**	**-2.06**	-2.42~-1.69	**0.78**
Sulfur isotope	**2.04**	1.3~2.81	**0.78**	**0.77**	0.05~1.51	**0.52**
Sea temperature	**-1.08**	-1.99~-0.07	**0.6**	0.75	-0.17~1.61	0.5

Supplementary Table 12. Asselian-Wordian epifaunal brachiopods

	Gλ	Gλ_HPDI	$w\lambda$	Gμ	Gμ_HPDI	$w\mu$
Infaunal bivalve div.	-1.3	-6.35~0.67	0.44	0.94	-1.45~6.16	0.39
Epifaunal bivalve div.	0.43	-1.96~3.61	0.35	1	-1.27~5.28	0.42
Epifaunal brachiopod div.	0.31	-2.08~3.49	0.33	-1.76	-7.89~1.17	0.48
Predator diversity	**-1.41**	-3.58~0.29	**0.55**	**-1.26**	-3.54~0.43	**0.54**
Proportion of carbonate occs.	0.2	-0.57~1.16	0.27	-0.48	-1.36~0.22	0.36
Continental fragmentation	0.86	-0.55~3.37	0.4	0.29	-1.01~2.41	0.31
Sea level	0.27	-1.24~2.7	0.37	0.02	-2.09~2.5	0.32
Carbon isotope	-0.01	-0.72~0.73	0.23	0.62	-0.24~1.82	0.4
Strontium isotope	0.28	-0.97~2.33	0.35	-1.08	-5.08~0.93	0.44
Sulfur isotope	0.2	-0.67~1.41	0.26	0.36	-0.84~2.66	0.32
Sea temperature	**-1.67**	-3.7~0.15	**0.6**	**-1.27**	-3.8~0.24	**0.51**

Supplementary Table 13. Capitanian-Ladinian epifaunal brachiopods

	Gλ	Gλ_HPDI	*w*λ	Gμ	Gμ_HPDI	*w*μ
Infaunal bivalve div.	**-4.68**	-8.28~-0.92	**0.9**	**-1.51**	-4~0.88	**0.66**
Epifaunal bivalve div.	**0.62**	-1.96~3.65	**0.54**	**-0.9**	-5.09~2.71	**0.62**
Epifaunal brachiopod div.	-0.03	-2.29~2	0.47	**-1.29**	-5.24~1.63	**0.64**
Predator diversity	**2.45**	-0.07~4.74	**0.78**	**2.49**	0.1~4.69	**0.79**
Proportion of carbonate occs.	**1.08**	-0.06~2.03	**0.62**	**-0.77**	-2.19~0.33	**0.51**
Continental fragmentation	**-0.91**	-1.91~0.14	**0.57**	0.27	-0.94~1.51	0.43
Sea level	-0.58	-1.45~0.19	0.48	-0.02	-0.84~0.8	0.37
Carbon isotope	**1.22**	-0.15~2.54	**0.62**	**-0.63**	-1.27~0.04	**0.51**
Strontium isotope	**0.58**	-0.92~2.64	**0.52**	**-4.23**	-6.09~-2.65	**0.92**
Sulfur isotope	**4.86**	1.37~7.7	**0.91**	**1.73**	-0.13~3.94	**0.7**
Sea temperature	**-6.18**	-10.26~-2.26	**0.94**	**-0.64**	-2.75~1.2	**0.54**

Supplementary Table 14. Carnian-Toarcian epifaunal brachiopods

	Gλ	Gλ_HPDI	*w*λ	Gμ	Gμ_HPDI	*w*μ
Infaunal bivalve div.	**-2.28**	-4.04~0.02	**0.75**	-0.16	-2.03~1.89	0.37
Epifaunal bivalve div.	-0.63	-2.35~0.58	0.44	0.45	-0.65~2.58	0.38
Epifaunal brachiopod div.	-0.76	-2.15~0.27	0.46	-0.92	-2.7~0.26	0.49
Predator diversity	-0.42	-1.76~0.68	0.36	**1.05**	-0.25~2.54	**0.54**
Proportion of carbonate occs.	-0.33	-1.06~0.22	0.32	-0.63	-1.4~0.1	0.43
Continental fragmentation	0.44	-0.62~2.54	0.37	**-1.52**	-3.89~0.26	**0.6**
Sea level	-0.48	-1.93~0.63	0.41	-0.51	-1.76~0.4	0.39
Carbon isotope	**2.11**	-0.05~3.41	**0.74**	**1.52**	-0.02~2.9	**0.64**
Strontium isotope	**-1.58**	-3~-0.06	**0.65**	-0.24	-1.38~0.81	0.3
Sulfur isotope	-0.43	-1.78~0.54	0.34	0.56	-0.62~2.25	0.39
Sea temperature	**0.97**	-0.11~2.15	**0.51**	-0.04	-0.77~0.71	0.28

Supplementary Table 15. Aalenian-Tithonian epifaunal brachiopods

	Gλ	Gλ_HPDI	*w*λ	Gμ	Gμ_HPDI	*w*μ
Infaunal bivalve div.	-1.26	-3.4~0.31	0.5	-0.53	-2.46~0.54	0.33
Epifaunal bivalve div.	0.03	-1.31~1.25	0.26	0.38	-0.85~2.28	0.29
Epifaunal brachiopod div.	**1.75**	0.17~3.29	**0.67**	0.32	-0.38~1.54	0.28
Predator diversity	0.61	-1.35~3.36	0.35	-0.08	-1.65~1.24	0.26
Proportion of carbonate occs.	-0.8	-2.32~0.27	0.41	-0.12	-1.46~0.74	0.25
Continental fragmentation	**-1.23**	-3.01~0.29	**0.54**	-0.53	-1.81~0.45	0.35
Sea level	0.03	-1.52~1.42	0.28	-0.38	-1.34~0.39	0.31
Carbon isotope	-0.19	-0.96~0.32	0.23	0.15	-0.43~0.83	0.2
Strontium isotope	0.75	-0.15~1.85	0.44	-0.71	-1.49~0.08	0.42
Sulfur isotope	0.17	-0.77~1.5	0.27	-0.52	-2.21~0.59	0.35
Sea temperature	-0.33	-1.52~0.4	0.29	-0.8	-1.86~0.16	0.44

Supplementary Table 16. Permian-Jurassic epifaunal bivalves

	Gλ	Gλ_HPDI	wλ	Gμ	Gμ_HPDI	wμ
Infaunal bivalve div.	**-4.91**	-7.83~-2.5	**0.92**	**-2.96**	-5.12~-0.85	**0.82**
Epifaunal bivalve div.	0.35	-0.61~1.67	0.35	-0.32	-1.64~0.68	0.33
Epifaunal brachiopod div.	-0.3	-1.46~0.39	0.31	**-1.22**	-2.59~0.15	**0.58**
Predator diversity	**2.32**	0.36~4.43	**0.75**	-0.12	-1.65~1.41	0.34
Proportion of carbonate occs.	0.16	-0.38~0.83	0.25	-0.69	-1.5~0.11	0.43
Continental fragmentation	0.13	-0.72~1.16	0.28	**1.36**	0.12~2.53	**0.63**
Sea level	0.15	-0.53~0.86	0.28	-0.1	-0.93~0.65	0.27
Carbon isotope	0.15	-0.85~1.51	0.33	-0.57	-1.73~0.45	0.41
Strontium isotope	0.55	-0.02~1.11	0.42	-0.54	-1.23~0.07	0.39
Sulfur isotope	0.19	-0.45~1.05	0.27	**-2.24**	-3.62~-0.88	**0.78**
Sea temperature	-0.11	-1.13~0.93	0.29	**2.12**	-0.01~3.75	**0.73**

Supplementary Table 17. Asselian-Wordian epifaunal bivalves

	Gλ	Gλ_HPDI	wλ	Gμ	Gμ_HPDI	wμ
Infaunal bivalve div.	-0.29	-1.97~1.24	0.24	-0.35	-2.47~0.97	0.26
Epifaunal bivalve div.	-0.39	-2.14~0.8	0.27	-0.19	-2.46~1.01	0.25
Epifaunal brachiopod div.	-0.54	-2.66~0.75	0.3	-0.47	-3.05~0.97	0.27
Predator diversity	-0.03	-1.45~1.3	0.21	-0.54	-3.38~0.74	0.3
Proportion of carbonate occs.	-0.04	-0.72~0.45	0.14	-0.1	-1.23~0.77	0.18
Continental fragmentation	0	-0.89~0.81	0.16	-0.4	-2.31~0.78	0.26
Sea level	0.03	-1.05~1.06	0.18	-0.38	-2.79~1.43	0.28
Carbon isotope	-0.22	-1.13~0.53	0.19	-0.12	-1.4~0.85	0.18
Strontium isotope	0.2	-1.06~1.7	0.21	-1.18	-5.01~0.52	0.41
Sulfur isotope	0.12	-0.86~1.24	0.18	-0.66	-2.86~0.55	0.32
Sea temperature	-0.46	-2.11~0.58	0.28	-0.05	-1.81~1.16	0.22

Supplementary Table 18. Capitanian-Ladinian epifaunal bivalves

	Gλ	Gλ_HPDI	wλ	Gμ	Gμ_HPDI	wμ
Infaunal bivalve div.	-0.86	-2.82~0.71	0.43	-0.58	-3.61~1.99	0.44
Epifaunal bivalve div.	-0.16	-2.11~1.54	0.36	**-3.01**	-7.92~0.58	**0.71**
Epifaunal brachiopod div.	-1.05	-3.42~0.83	0.5	**1.74**	-0.92~6.8	**0.53**
Predator diversity	0.7	-0.39~2.24	0.41	1.16	-0.37~3.4	0.5
Proportion of carbonate occs.	0.36	-0.45~1.38	0.33	-0.66	-1.98~0.38	0.4
Continental fragmentation	0.22	-0.7~1.31	0.29	-0.62	-2.62~0.65	0.39
Sea level	**-1.34**	-2.62~0.04	**0.59**	-0.85	-2.21~0.11	0.46
Carbon isotope	-0.17	-2.14~1.47	0.38	-0.47	-2.15~0.65	0.35
Strontium isotope	0.21	-0.93~1.67	0.32	0.21	-1.05~1.73	0.31
Sulfur isotope	0.21	-1~1.75	0.3	-0.86	-2.97~0.77	0.47
Sea temperature	0.44	-0.9~2.87	0.38	0.21	-1.23~2.53	0.34

Supplementary Table 19. Carnian-Toarcian epifaunal bivalves

	Gλ	Gλ_HPDI	wλ	Gμ	Gμ_HPDI	wμ
Infaunal bivalve div.	**-4.16**	-6.25~-1.89	**0.89**	-0.55	-2.48~0.71	0.37
Epifaunal bivalve div.	1.01	-0.58~3.51	0.45	0.2	-0.93~1.88	0.29
Epifaunal brachiopod div.	-1.11	-3.46~0.33	0.48	**-1.3**	-3.49~0.27	**0.52**
Predator diversity	-0.16	-1.5~0.78	0.27	-0.65	-2.47~0.62	0.39
Proportion of carbonate occs.	0.08	-0.76~0.85	0.24	0.48	-0.37~1.96	0.34
Continental fragmentation	-0.33	-2.6~1.56	0.35	**-1.4**	-4.02~0.58	**0.55**
Sea level	-0.17	-1.44~0.86	0.27	0.21	-0.71~1.52	0.29
Carbon isotope	0.32	-0.75~1.8	0.32	0.37	-0.96~1.93	0.34
Strontium isotope	**-2.43**	-3.89~-0.7	**0.78**	-0.33	-1.6~0.55	0.29
Sulfur isotope	-0.23	-1.75~0.95	0.29	-0.15	-1.44~0.91	0.27
Sea temperature	0.95	-0.3~2.55	0.48	-0.14	-1.05~0.58	0.25

Supplementary Table 20. Aalenian-Tithonian epifaunal bivalves

	Gλ	Gλ_HPDI	wλ	Gμ	Gμ_HPDI	wμ
Infaunal bivalve div.	-0.36	-1.88~0.65	0.21	-0.08	-0.84~0.56	0.13
Epifaunal bivalve div.	-0.02	-1.02~0.79	0.15	-0.3	-1.62~0.55	0.21
Epifaunal brachiopod div.	0.17	-0.58~1.09	0.16	-0.05	-0.94~0.66	0.15
Predator diversity	0	-1.16~0.99	0.15	-0.24	-1.7~0.57	0.18
Proportion of carbonate occs.	-0.19	-1.36~0.52	0.16	-0.68	-2.68~0.45	0.3
Continental fragmentation	-0.53	-2.64~0.41	0.24	-0.14	-1.23~0.73	0.16
Sea level	-0.05	-0.7~0.59	0.12	-0.02	-0.67~0.75	0.14
Carbon isotope	0.13	-0.57~1.16	0.16	0.13	-0.71~1.29	0.16
Strontium isotope	-0.23	-1.39~0.51	0.18	0.05	-0.56~0.84	0.14
Sulfur isotope	-0.28	-1.79~0.55	0.19	-0.13	-1.61~0.94	0.17
Sea temperature	-0.11	-1.08~0.83	0.17	**-2.13**	-3.98~0.12	**0.69**

Supplementary Table 21. Permian-Jurassic infaunal bivalves

	Gλ	Gλ_HPDI	wλ	Gμ	Gμ_HPDI	wμ
Infaunal bivalve div.	**-3.84**	-6.38~-0.55	**0.86**	**-2.2**	-4.74~0.31	**0.69**
Epifaunal bivalve div.	-0.52	-2.04~0.49	0.39	**-1**	-2.71~0.36	**0.53**
Epifaunal brachiopod div.	-0.63	-1.96~0.28	0.44	-0.83	-2.56~0.4	0.48
Predator diversity	**2.83**	0.32~5.06	**0.81**	-0.09	-1.64~1.62	0.36
Proportion of carbonate occs.	0.52	-0.14~1.33	0.42	-0.7	-1.58~0.13	0.46
Continental fragmentation	-0.26	-1.48~0.67	0.34	**2.16**	0.67~3.89	**0.76**
Sea level	0.01	-0.71~0.83	0.29	-0.68	-1.93~0.37	0.46
Carbon isotope	0.62	-0.51~2.36	0.44	-0.3	-2.22~1.15	0.41
Strontium isotope	0.28	-0.27~0.91	0.31	-0.31	-1.16~0.24	0.34
Sulfur isotope	-0.08	-0.95~0.63	0.28	**-3.46**	-5.02~-1.57	**0.87**
Sea temperature	0.19	-0.78~1.19	0.31	**2.61**	0.56~4.4	**0.79**

Supplementary Table 22. Asselian-Wordian infaunal bivalves

	Gλ	Gλ_HPDI	$w\lambda$	Gμ	Gμ_HPDI	$w\mu$
Infaunal bivalve div.	-0.71	-3.86~1.03	0.39	-0.72	-3.96~0.89	0.35
Epifaunal bivalve div.	-0.41	-3.5~1.17	0.31	-0.18	-2.36~1.39	0.27
Epifaunal brachiopod div.	-1.11	-4.58~0.94	0.43	-0.67	-4.08~1.06	0.35
Predator diversity	-0.38	-2.46~0.96	0.26	-0.79	-4.21~0.6	0.36
Proportion of carbonate occs.	-0.01	-1~0.87	0.2	0.1	-0.95~1.4	0.23
Continental fragmentation	-0.28	-1.98~0.83	0.26	-0.78	-3.13~0.7	0.37
Sea level	0.3	-0.97~2.13	0.24	-0.02	-1.51~1.57	0.24
Carbon isotope	-0.1	-1.21~1.06	0.22	-0.07	-1.35~1.07	0.23
Strontium isotope	0.03	-1.22~2.13	0.25	-0.44	-2.93~1.29	0.31
Sulfur isotope	-0.02	-1.43~1.39	0.24	-0.29	-2.09~0.81	0.26
Sea temperature	-0.81	-3.93~0.55	0.36	-0.35	-2.6~1.3	0.27

Supplementary Table 23. Capitanian-Ladinian infaunal bivalves

	Gλ	Gλ_HPDI	$w\lambda$	Gμ	Gμ_HPDI	$w\mu$
Infaunal bivalve div.	-0.44	-3.43~1.25	0.41	**-3.5**	-6.56~0.14	**0.82**
Epifaunal bivalve div.	**-1.54**	-4.63~0.6	**0.58**	-0.21	-3.87~2.54	0.41
Epifaunal brachiopod div.	**-1.29**	-4.91~1.07	**0.52**	0.61	-1.09~3.55	0.39
Predator diversity	0.41	-0.98~2.29	0.38	0.6	-1.2~2.79	0.4
Proportion of carbonate occs.	0.19	-0.74~1.4	0.29	-0.13	-1.57~0.88	0.31
Continental fragmentation	-0.24	-1.61~0.93	0.32	-0.18	-1.38~1	0.3
Sea level	**-1.46**	-3.73~0.41	**0.56**	-0.71	-2.64~0.5	0.42
Carbon isotope	0.6	-0.78~2.65	0.39	0.01	-1.78~1.57	0.32
Strontium isotope	0.53	-0.89~2.1	0.38	0.28	-1.22~2.19	0.34
Sulfur isotope	-0.17	-2.15~1.13	0.33	**-2.64**	-5.75~0.1	**0.74**
Sea temperature	0.37	-1.46~3.18	0.35	0.95	-0.85~4.25	0.47

Supplementary Table 24. Carnian-Toarcian infaunal bivalves

	Gλ	Gλ_HPDI	$w\lambda$	Gμ	Gμ_HPDI	$w\mu$
Infaunal bivalve div.	**-1.86**	-4.8~0.31	**0.62**	-0.96	-3.96~0.7	0.46
Epifaunal bivalve div.	0.49	-0.73~2.56	0.35	-0.43	-2.55~0.96	0.35
Epifaunal brachiopod div.	-0.95	-2.95~0.31	0.44	-0.51	-2.23~0.76	0.38
Predator diversity	-0.27	-1.7~0.75	0.29	-0.15	-1.74~1.1	0.3
Proportion of carbonate occs.	0.03	-0.82~0.84	0.23	-0.52	-2.36~0.85	0.38
Continental fragmentation	**-1.5**	-5.74~0.57	**0.51**	**-2.08**	-5.5~0.37	**0.63**
Sea level	-0.56	-2.45~0.62	0.35	-0.22	-1.81~0.83	0.3
Carbon isotope	0.69	-0.85~2.69	0.41	-0.16	-1.94~1.37	0.29
Strontium isotope	**-2.16**	-4.12~0.16	**0.71**	-0.16	-1.52~0.95	0.28
Sulfur isotope	-0.12	-1.63~0.9	0.26	0.33	-0.86~2.14	0.32
Sea temperature	0.83	-0.38~2.38	0.43	0.15	-0.61~1.32	0.27

Supplementary Table 25. Aalenian-Tithonian infaunal bivalves

	Gλ	Gλ_HPDI	$w\lambda$	Gμ	Gμ_HPDI	$w\mu$
Infaunal bivalve div.	-0.18	-1.4~0.49	0.19	-0.24	-1.49~0.77	0.21
Epifaunal bivalve div.	-0.11	-1.37~0.94	0.19	-0.49	-2.41~0.72	0.29
Epifaunal brachiopod div.	0.16	-0.4~1.09	0.17	0.04	-0.52~0.75	0.14
Predator diversity	0.31	-0.84~2.12	0.23	-0.61	-2.58~0.44	0.33
Proportion of carbonate occs.	0.11	-0.49~0.89	0.17	-0.4	-2.27~0.53	0.26
Continental fragmentation	-0.98	-3.15~0.38	0.4	0.02	-1.25~0.91	0.18
Sea level	0.02	-0.61~0.81	0.14	-0.19	-1.09~0.48	0.18
Carbon isotope	-0.09	-1.14~0.54	0.16	-0.03	-1.06~1.2	0.18
Strontium isotope	0.04	-0.64~0.63	0.14	-0.43	-1.81~0.23	0.26
Sulfur isotope	-1.08	-3.43~0.44	0.42	-0.07	-1.57~0.92	0.21
Sea temperature	-0.02	-0.94~0.7	0.16	**-1.54**	-3.3~0.28	**0.58**
