# Supplementary material for: Bayesian analyses indicate bivalves did not drive the downfall of brachiopods following the Permian-Triassic mass extinction
Source: Nat Commun. 2023 Sep 9;14:5566. doi: 10.1038/s41467-023-41358-8 (PMC10492784; doi:10.1038/s41467-023-41358-8)
Supplement: Supplementary file 3 — Reporting Summary [file 41467_2023_41358_MOESM3_ESM.pdf]

nature portfolio

Corresponding author(s): Michael J. Benton; Zhong-Qiang Chen

Last updated by author(s): Aug 6, 2023

Reporting Summary

Nature Portfolio wishes to improve the reproducibility of the work that we publish. This form provides structure for consistency and transparency in reporting. For further information on Nature Portfolio policies, see our Editorial Policies and the Editorial Policy Checklist.

Statistics

For all statistical analyses, confirm that the following items are present in the figure legend, table legend, main text, or Methods section.

n/a	Confirmed	
x		The exact sample size (*n*) for each experimental group/condition, given as a discrete number and unit of measurement
x		A statement on whether measurements were taken from distinct samples or whether the same sample was measured repeatedly
	x	The statistical test(s) used AND whether they are one- or two-sided<br>*Only common tests should be described solely by name; describe more complex techniques in the Methods section.*
x		A description of all covariates tested
x		A description of any assumptions or corrections, such as tests of normality and adjustment for multiple comparisons
	x	A full description of the statistical parameters including central tendency (e.g. means) or other basic estimates (e.g. regression coefficient) AND variation (e.g. standard deviation) or associated estimates of uncertainty (e.g. confidence intervals)
	x	For null hypothesis testing, the test statistic (e.g. *F*, *t*, *r*) with confidence intervals, effect sizes, degrees of freedom and *P* value noted<br>*Give P values as exact values whenever suitable.*
	x	For Bayesian analysis, information on the choice of priors and Markov chain Monte Carlo settings
x		For hierarchical and complex designs, identification of the appropriate level for tests and full reporting of outcomes
	x	Estimates of effect sizes (e.g. Cohen's *d*, Pearson's *r*), indicating how they were calculated

Our web collection on statistics for biologists contains articles on many of the points above.

Software and code

Policy information about availability of computer code

Data collection	No software was used for data collection.
Data analysis	Most analyses were performed using R (v4.1). Bayesian analyses were conducted using PyRate (v3), an open source software package available on Github (http://github.com/dsilvestro/PyRate). Posterior samples were checked using Tracer (v.1.7.1), a software available on Github (http://github.com/beast-dev/tracer/releases/tag/v1.7.1). The maps of the PALEOMAP project were accessed using the GPlates Web Service (https://github.com/GPlates/gplates-web-service). Additionally, these R packages are were used in processing the data: fossilbrush (v1.0.3), icosa (v0.11.0), igraph (v1.5.0.1), fpc (2.2-10), divDyn (0.8.2).<br>All code used to analyse the data are deposited in Zenodo (https://doi.org/10.5281/zenodo.8216739).

For manuscripts utilizing custom algorithms or software that are central to the research but not yet described in published literature, software must be made available to editors and reviewers. We strongly encourage code deposition in a community repository (e.g. GitHub). See the Nature Portfolio guidelines for submitting code & software for further information.

Data

Policy information about availability of data

All manuscripts must include a data availability statement. This statement should provide the following information, where applicable:

- Accession codes, unique identifiers, or web links for publicly available datasets
- A description of any restrictions on data availability
- For clinical datasets or third party data, please ensure that the statement adheres to our policy

Most data were downloaded from Paleobiology Database (https://paleobiodb.org/). In addition, we added some Permian–Jurassic fossil occurrences collected from original references. The URLs used to download the data, all added, raw and revised data analysed in this study are available in Zenodo (https://doi.org/10.5281/zenodo.8216739).

Human research participants

Policy information about studies involving human research participants and Sex and Gender in Research.

Reporting on sex and gender	This is not a human research.
Population characteristics	This is not a human research.
Recruitment	This is not a human research.
Ethics oversight	This is not a human research.

Note that full information on the approval of the study protocol must also be provided in the manuscript.

Field-specific reporting

Please select the one below that is the best fit for your research. If you are not sure, read the appropriate sections before making your selection.

☐ Life sciences ☐ Behavioural & social sciences ☒ Ecological, evolutionary & environmental sciences

For a reference copy of the document with all sections, see nature.com/documents/nr-reporting-summary-flat.pdf

Ecological, evolutionary & environmental sciences study design

All studies must disclose on these points even when the disclosure is negative.

Study description	This study uses the Bayesian method to calculate diversity and diversification (including origination, extinction, and net diversification) rates of brachiopods and bivalves and analyse their relationship with biotic and abiotic factors. Firstly, we analysed the diversification dynamic of global data. Then, we analysed the rates of taxa from different geographic regions and taxa with different ecological lifestyles. Ten age randomised replicates were conducted for every analysis.
Research sample	The analysed data are global fossil occurrences data of brachiopods and bivalves. Most data were downloaded from Paleobiology Database (https://paleobiodb.org). We also added some Permian to Jurassic fossil occurrences data collected from primary references, which have not been included by the Paleobiology Database. All added and analysed data are provided in Zenodo. These occurrences are composed of taxonomic names, collection information, locations (i.e., coordinates), stratigraphic horizons, and ages.
Sampling strategy	No sampling strategy was applied for the analysis of global datasets because all fossil occurrences were included. For regional analyses, we used the sampling procedure of Flannery-Sutherland et al. (2023) to make sure the data analysed is spatially standardised. Before sampling, each fossil occurrence was given a paleocoordination. We defined several geographical regions based on the paleobiogeographical pattern of fossil occurrences. Next, spatial windows were constructed for these regions using the spacetimewind function. These windows are stable in size and can move over time to track the movement of the focal plate. Last, fossil occurrences in each spatial window were sampled and standardised using the spacetimestand function. We applied the minimum spanning tree length as the metric to standardise the occurrence data. In this procedure, fossil occurrences are binned into hexagonal grids, and a minimum spanning tree is reconstructed from the grid centres that contain fossil data. If the MST length is greater than the target value, the cells with the smallest amount of data are removed from the MST, until the threshold is reached. We calculated the MST length of raw data in each time bin, and the median MST length of all time bins was adopted as the threshold. The sampled occurrences were used to calculate the diversification rates of that region.
Data collection	Occurrences from Paleobiology Database were downloaded manually by Z.G. on November 23, 2022. We also added 1,522 and 4,538 occurrences of Permian–Jurassic brachiopods and bivalves. These added ones were collected by Z.G. from primary literature and recorded in a spreadsheet. The raw datasets including taxonomic names, stratigraphic horizons, collection information, ages, ecological lifestyles and coordinations. The URLs used to download the data and the source literature of added data are listed in Zenodo (https://doi.org/10.5281/zenodo.8216739).

Timing and spatial scale	The timing scale of our study is from Ordovician to Quaternary, and we did additional and detailed analyses for the Permian to Jurassic periods. The spatial scale is global. For regional analysis, four regions (northern Panthalassa, north-western Tethys, south-western Tethys, and eastern Tethys) were defined by spatial windows suggested by the palaeobiogeography of fossil occurrences. The data from Paleobiology Database was downloaded on November 23, 2022.
Data exclusions	We deleted some doubtful records according to recent taxonomic opinions, previously published and well-curated databases, and result of the pacmacro_ranges function in R fossilbruch package. We also revised the ages of the fossil occurrences according to recently published ages of their stratigraphic units. The code used to revise and clean the datasets are also provided in Zenodo (https://doi.org/10.5281/zenodo.8216739).
Reproducibility	All data and code needed to recreate the analyses are present in Zenodo (https://doi.org/10.5281/zenodo.8216739).
Randomization	Samples were divided into temporal bins based on their stratigraphical age.
Blinding	Blinding was not required because we used all data in the analyses. There is no experiments in which information may influence the result.

Did the study involve field work? ☐ Yes ☒ No

Reporting for specific materials, systems and methods

We require information from authors about some types of materials, experimental systems and methods used in many studies. Here, indicate whether each material, system or method listed is relevant to your study. If you are not sure if a list item applies to your research, read the appropriate section before selecting a response.

Materials & experimental systems

n/a	Involved in the study
☒	☐ Antibodies
☒	☐ Eukaryotic cell lines
☒	☐ Palaeontology and archaeology
☒	☐ Animals and other organisms
☒	☐ Clinical data
☒	☐ Dual use research of concern

Methods

n/a	Involved in the study
☒	☐ ChIP-seq
☒	☐ Flow cytometry
☒	☐ MRI-based neuroimaging
